# Characterization of wall-associated kinase/wall-associated kinase-like (WAK/WAKL) family in rose (*Rosa chinensis*) reveals the role of *RcWAK4* in Botrytis resistance

**DOI:** 10.1186/s12870-021-03307-9

**Published:** 2021-11-10

**Authors:** Xintong Liu, Zicheng Wang, Yu Tian, Shiya Zhang, Dandan Li, Wenqi Dong, Changqing Zhang, Zhao Zhang

**Affiliations:** Department of Ornamental Horticulture, Beijing Key Laboratory of Development and Quality Control of Ornamental Crops, China Agricultural University, Yuanmingyuan Xilu 2, Beijing, 100193 China

**Keywords:** *Rosa chinensis*, WAK, WAKL, *Botrytis cinerea*, grey mold disease, immune response

## Abstract

**Background:**

Wall-associated kinase (WAK)/WAK-like (WAKL) is one of the subfamily of receptor like kinases (RLK). Although previous studies reported that WAK/WAKL played an important role in plant cell elongation, response to biotic and abiotic stresses, there are no systematic studies on RcWAK/RcWAKL in rose.

**Results:**

In this study, we identified a total of 68 RcWAK/RcWAKL gene family members within rose (*Rosa chinensis*) genome. The RcWAKs contained the extracellular galacturonan-binding domain and calcium-binding epidermal growth factor (EGF)-like domain, as well as an intracellular kinase domains. The RcWAKLs are missing either calcium-binding EGF-like domain or the galacturonan-binding domain in their extracellular region. The phylogenetic analysis showed the RcWAK/RcWAKL gene family has been divided into five groups, and these *RcWAK/RcWAKL* genes were unevenly distributed on the 7 chromosomes of rose. 12 of *RcWAK/RcWAKL* genes were significantly up-regulated by *Botrytis cinerea*-inoculated rose petals, where *RcWAK4* was the most strongly expressed. Virus induced gene silencing of *RcWAK4* increased the rose petal sensitivity to *B. cinerea.* The results indicated *RcWAK4* is involved in the resistance of rose petal against *B. cinerea*.

**Conclusion:**

Our study provides useful information to further investigate the function of the RcWAK/RcWAKL gene family and breeding research for resistance to *B. cinerea* in rose.

**Supplementary Information:**

The online version contains supplementary material available at 10.1186/s12870-021-03307-9.

## Background

Plant defense strategies of necrotrophic pathogens such as *Botrytis cinerea* are various. Pathogen-derived microbial-associated molecular patterns (MAMPs) and host damage-associated molecular patterns (DAMPs) as two immune signalings active plant immunity [1]. The plant cell wall is the first barrier to plant defense responses [2], and also plays an important role in maintaining cell morphology, regulating plant growth and development, and responding to biotic and abiotic stresses [3]. Pattern recognition receptors (PRRs) are a class of receptor kinases localized in the cell membrane that recognize pathogen invasion and transmit danger signals in plants [4]. Wall-associated kinase (WAK)/ WAK-like (WAKL) is a typical category of PRRs classified to receptor-like protein kinases (RLKs) [5].

WAKs/WAKLs are transmembrane proteins with an intracellular Ser/Thr kinase domain and an extracellular structure, contacting extracellular and cytoplasmic signals. Their extracellular structure contains a galacturonan-binding domain and/or a calcium-binding epidermal growth factor (EGF)-like domain for signal perception [6]. WAKs/WAKLs play important roles in regulating plant growth, development, and responding to environmental stresses. Expression of the *AtWAK4* antisense gene in *Arabidopsis* results in impaired cell elongation and stunted lateral root development [7]. In addition, knocked out *CaWAKL20* significantly increased pepper heat tolerance, whereas overexpression of *CaWAKL20* in *Arabidopsis* reduced heat tolerance, indicating that *CaWAKL20* negatively regulated pepper heat stress response [8].

Meanwhile, WAKs/WAKLs play a vital role in plant defense response to fungal diseases. Oligogalactouronides (OGs), as the cell wall polysaccharide degradation productions, are recognized by AtWAK1. And overexpression *AtWAK1* could enhance resistance to *B. cinerea* in *Arabidopsis* [9, 10]. The high expression of *ZmWAK*, which was located in the head smut quantitative resistance locus (qHSP1), effectively suppressed the growth of *Sporisorium reilianum* in the mesocotyl [11]. Rice *OsWAK14*, *OsWAK91* and *OsWAK92* positively regulated the defense response to blast fungus (*Magnaporthe oryzae*), while *OsWAK112d* worked as a negative regulator of the defense response [12]. *SlWAK1* is involved in the regulation of PRR-mediated immune response through *FLS2/FLS3* complex in tomatoes [13].

Rose (*Rosa* sp.), as an important ornamental crop, accounts for more than 30% of the world's annual trade in cut flowers [14]. The long-distance transportation of cut rose flowers leads to water loss, senescence and postharvest diseases on cut flowers, resulting in serious economic losses. Gray mold disease, caused by *B. cinerea*, as one of the most serious postharvest fungal diseases of rose. The role WAKs/WAKLs family members in resistance against necrotrophic pathogens have been reported in *Arabidopsis* [9, 10]. However, whether WAKs/WAKLs are involved in rose resistance to *B. cinerea* is largely unknown.

Our previous study found that a large number of rose *WAK*/*WAKL* genes significantly up-regulated in response to *B. cinerea* inoculation [15]. In this study, we comprehensively characterized the of RcWAKs/RcWAKLs in rose genome, including their gene structural features, conserved domain, evolutionary relationships, and their expression pattern upon *B. cinerea* inoculation. This analysis suggested *RcWAK4* may play a role in the response to *B. cinerea* infection. We finally used virus-induced gene silencing (VIGS) to confirm that *RcWAK4* is involved in the rose defense against *B. cinerea*.

## Results

### Identification of RcWAK/ RcWAKL family members in rose

**Fig. 1:**
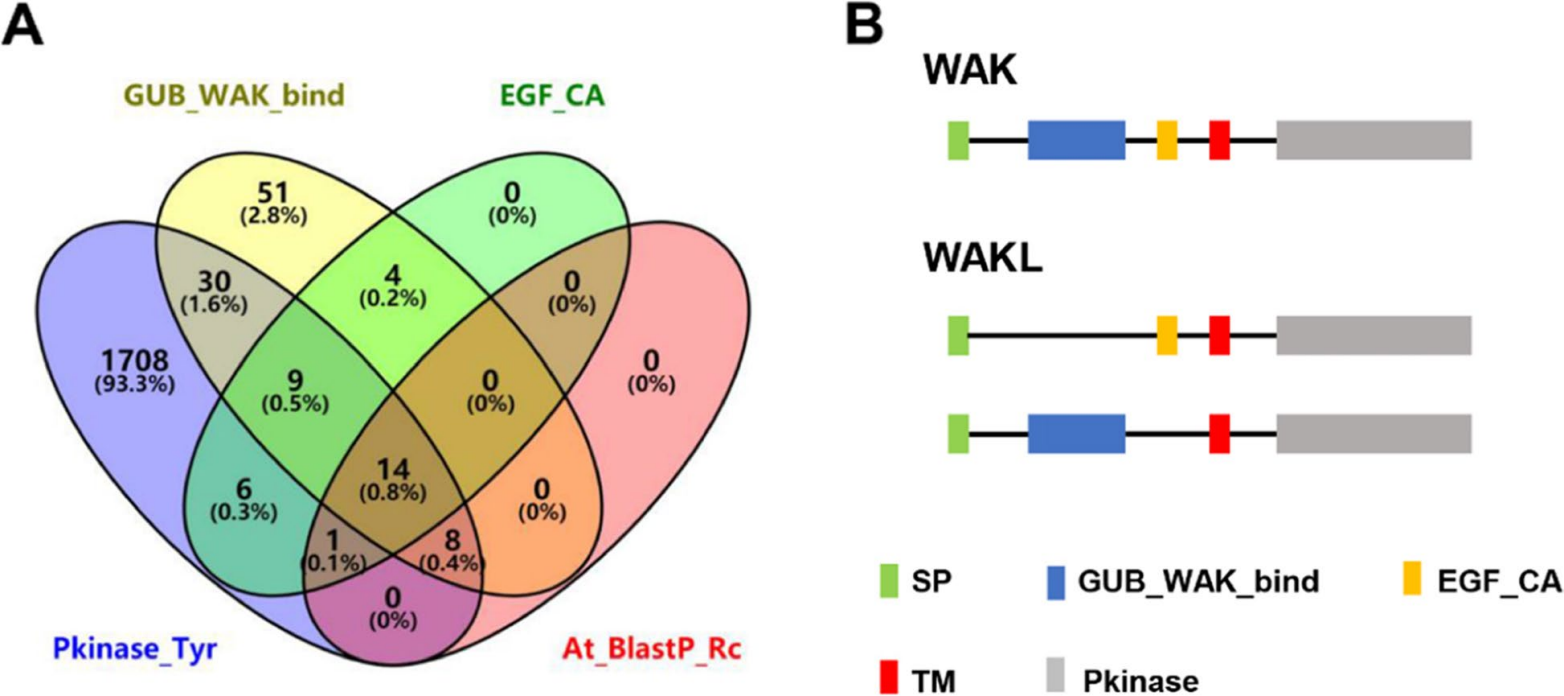
The predicted results of RcWAKs/RcWAKLs. **A** Venn diagram of the number of predicted RcWAKs/RcWAKLs or their conserved motifs by using Blastp or HMM search. At_BlastP_Rc represented the number of predicted RcWAKs/RcWAKLs by using Arabidopsis AtWAK/AtWAKL protein sequences to search for homologous proteins in *Rosa chinensis,* with E value<1e^-3^. GUB_WAK_bind, EGF_CA and PKinase_Ser/Thr represented number of proteins carring galacturonan-binding domain, calcium-binding EGF-like domain, or a kinase domain, respectively. **B** The Schematic representation of RcWAKs/RcWAKLs

To identify the WAK/WAKL gene family in *Rosa chinensis*, 27 *Arabidopsis* WAK/WAKL family members were used as a reference to perform BlastP (E value<0.001) in the rose genome database (*R. chinensis* Homozygous Genome v2.0; available at https://lipmbrowsers.toulouse.inra.fr/pub/RchiOBHm-V2/). A total of 23 homologous RcWAK/RcWAKL were obtained after BlastP. Then, the HMM files of the calcium-binding EGF-like domain (EGF_CA, PF07645.15), galacturonan-binding domain (GUB_WAK_bind, PF13947.6) and kinase domain (Pkinase_Tyr, PF07714.17) from the Pfam database (http://pfam.xfam.org/) were used for hmmsearch. The EGF_CA, GUB_WAK_bind and Pkinase_Tyr hmmsearch lead to the identification of 34, 116 and 1723 candidate proteins, respectively (Fig. 1). Finally, we have verified a total of 68 non-redundant RcWAK/RcWAKL family genes in rose genome, where 23 candidate genes contained EGF_CA, GUB_WAK_bind and Phkinase_Tyr conserved domains, and considered as RcWAK genes. The other candidate genes were RcWAKLs, of which 38 contained GUB_WAK_bind and Phkinase_Tyr domain, the remaining 7 contained EGF_CA and Pkinase_Tyr domain (Fig. 1).

The length of RcWAKs and RcWAKLs was extremely varied. The shortest is RcWAKL41, which encodes 369 amino acids. The longest is RcWAKL11, it encodes a protein of 891 amino acids. The average length of RcWAK/RcWAKL proteins was 678 amino acids. The accession number, chromosomal location, numbers of exon and intron, length of CDS and subgroup of RcWAK/RcWAKL gene family were listed in Table 1.

**Table 1:** Predicted members of RcWAK/RcWAKL family in ‘*Rosa chinensis*’

Gene	Accession number ^a^	Chr.^b^	Position ^c^	Intron	Exon	CDS (bp)	Amino Acids	Group
RcWAK1	RchiOBHm_Chr1g0329511	1	19.17431	2	3	2328	775	I
RcWAK2	RchiOBHm_Chr1g0331391	1	21.25817	1	2	2346	781	II
RcWAK3	RchiOBHm_Chr1g0336911	1	28.55835	2	3	2364	787	I
RcWAK4	RchiOBHm_Chr2g0152591	2	69.97138	2	3	2190	729	III
RcWAK5	RchiOBHm_Chr2g0152901	2	70.29155	2	3	2223	740	III
RcWAK6	RchiOBHm_Chr2g0175101	2	87.45908	3	4	1794	597	II
RcWAK7	RchiOBHm_Chr5g0016551	5	11.37102	2	3	2157	718	I
RcWAK8	RchiOBHm_Chr5g0016611	5	11.43604	3	4	2280	759	I
RcWAK9	RchiOBHm_Chr5g0016871	5	11.56994	2	3	1848	615	I
RcWAK10	RchiOBHm_Chr5g0016931	5	11.65443	2	3	2331	776	I
RcWAK11	RchiOBHm_Chr5g0017041	5	11.72528	2	3	2094	697	I
RcWAK12	RchiOBHm_Chr5g0017091	5	11.76752	1	2	1545	514	I
RcWAK13	RchiOBHm_Chr5g0017291	5	11.98662	2	3	2337	778	I
RcWAK14	RchiOBHm_Chr5g0017321	5	12.00918	3	4	2490	829	I
RcWAK15	RchiOBHm_Chr5g0019001	5	13.45881	2	3	2325	774	I
RcWAK16	RchiOBHm_Chr5g0028941	5	22.72841	2	3	2325	774	I
RcWAK17	RchiOBHm_Chr5g0046061	5	42.10957	2	3	2286	761	III
RcWAK18	RchiOBHm_Chr5g0047341	5	43.74824	3	4	2244	747	III
RcWAK19	RchiOBHm_Chr5g0052141	5	53.75484	2	3	2256	751	I
RcWAK20	RchiOBHm_Chr5g0054801	5	57.50674	2	3	2304	767	I
RcWAK21	RchiOBHm_Chr6g0259291	6	14.53278	2	3	2352	783	I
RcWAK22	RchiOBHm_Chr6g0306911	6	65.42927	2	3	2262	753	II
RcWAK23	RchiOBHm_Chr7g0210751	7	28.16683	1	2	2352	783	III
RcWAKL1	RchiOBHm_Chr1g0328221	1	17.58247	1	2	1938	645	V
RcWAKL2	RchiOBHm_Chr1g0328281	1	17.62497	1	2	1851	616	V
RcWAKL3	RchiOBHm_Chr1g0328501	1	17.90567	5	6	1788	595	V
RcWAKL4	RchiOBHm_Chr1g0328701	1	18.10089	6	7	1464	487	V
RcWAKL5	RchiOBHm_Chr1g0333331	1	25.34515	4	5	2013	670	V
RcWAKL6	RchiOBHm_Chr1g0333351	1	25.40108	2	3	1893	630	V
RcWAKL7	RchiOBHm_Chr1g0333441	1	25.56581	2	3	1968	655	V
RcWAKL8	RchiOBHm_Chr1g0333481	1	25.62574	1	2	2175	724	V
RcWAKL9	RchiOBHm_Chr1g0333571	1	25.70152	2	3	1968	655	V
RcWAKL10	RchiOBHm_Chr1g0333611	1	25.73956	2	3	1332	443	V
RcWAKL11	RchiOBHm_Chr1g0361321	1	53.32277	11	12	2676	891	IV
RcWAKL12	RchiOBHm_Chr1g0368501	1	58.6087	2	3	1884	627	IV
RcWAKL13	RchiOBHm_Chr2g0117481	2	29.64339	2	3	2007	668	III
RcWAKL14	RchiOBHm_Chr2g0150651	2	68.33199	2	3	1137	378	III
RcWAKL15	RchiOBHm_Chr2g0150741	2	68.39579	2	3	2208	735	III
RcWAKL16	RchiOBHm_Chr2g0150781	2	68.45249	2	3	2262	753	III
RcWAKL17	RchiOBHm_Chr2g0152581	2	69.95926	3	4	2178	725	III
RcWAKL18	RchiOBHm_Chr2g0152641	2	70.0389	2	3	2217	738	III
RcWAKL19	RchiOBHm_Chr3g0449381	3	1.182279	2	3	1875	624	V
RcWAKL20	RchiOBHm_Chr3g0471461	3	17.30996	0	1	1950	649	IV
RcWAKL21	RchiOBHm_Chr3g0476711	3	22.69575	2	3	1509	502	I
RcWAKL22	RchiOBHm_Chr4g0405811	4	26.57302	1	2	1881	626	V
RcWAKL23	RchiOBHm_Chr4g0434711	4	58.40836	1	2	1899	632	V
RcWAKL24	RchiOBHm_Chr4g0445241	4	65.73948	0	1	1920	639	IV
RcWAKL25	RchiOBHm_Chr5g0016561	5	11.37718	3	4	2109	702	I
RcWAKL26	RchiOBHm_Chr5g0016581	5	11.39569	2	3	2247	748	I
RcWAKL27	RchiOBHm_Chr5g0016601	5	11.43082	2	3	2208	735	I
RcWAKL28	RchiOBHm_Chr5g0016641	5	11.46383	2	3	1821	606	I
RcWAKL29	RchiOBHm_Chr5g0016881	5	11.57806	2	3	2262	753	I
RcWAKL30	RchiOBHm_Chr5g0017021	5	11.71215	3	4	1566	521	I
RcWAKL31	RchiOBHm_Chr5g0017141	5	11.87352	2	3	2268	755	I
RcWAKL32	RchiOBHm_Chr5g0017281	5	11.98236	2	3	2253	750	I
RcWAKL33	RchiOBHm_Chr5g0017331	5	12.01933	2	3	2310	769	I
RcWAKL34	RchiOBHm_Chr5g0017401	5	12.06204	1	2	2148	715	I
RcWAKL35	RchiOBHm_Chr5g0019071	5	13.55579	2	3	1296	431	I
RcWAKL36	RchiOBHm_Chr5g0047581	5	44.05517	3	4	2238	745	V
RcWAKL37	RchiOBHm_Chr6g0273041	6	33.64626	4	5	2082	693	IV
RcWAKL38	RchiOBHm_Chr6g0292991	6	55.81773	3	4	1554	517	II
RcWAKL39	RchiOBHm_Chr7g0185911	7	5.968027	1	2	1860	619	V
RcWAKL40	RchiOBHm_Chr7g0185931	7	5.988849	2	3	1956	651	V
RcWAKL41	RchiOBHm_Chr7g0185941	7	5.993237	2	3	1110	369	V
RcWAKL42	RchiOBHm_Chr7g0185971	7	6.0123	3	4	1989	662	IV
RcWAKL43	RchiOBHm_Chr7g0186001	7	6.026964	3	4	1974	657	IV
RcWAKL44	RchiOBHm_Chr7g0209921	7	27.47616	3	4	1872	623	V
RcWAKL45	RchiOBHm_Chr7g0221871	7	42.97117	2	3	2328	775	III

^a^Available at https://lipm-browsers.toulouse.inra.fr/pub/RchiOBHm-V2/

^b^Chromosome

^c^Starting position on chromosome (Mb)

### Chromosomal locations of rose *RcWAK/RcWAKL* genes

**Fig. 2:**
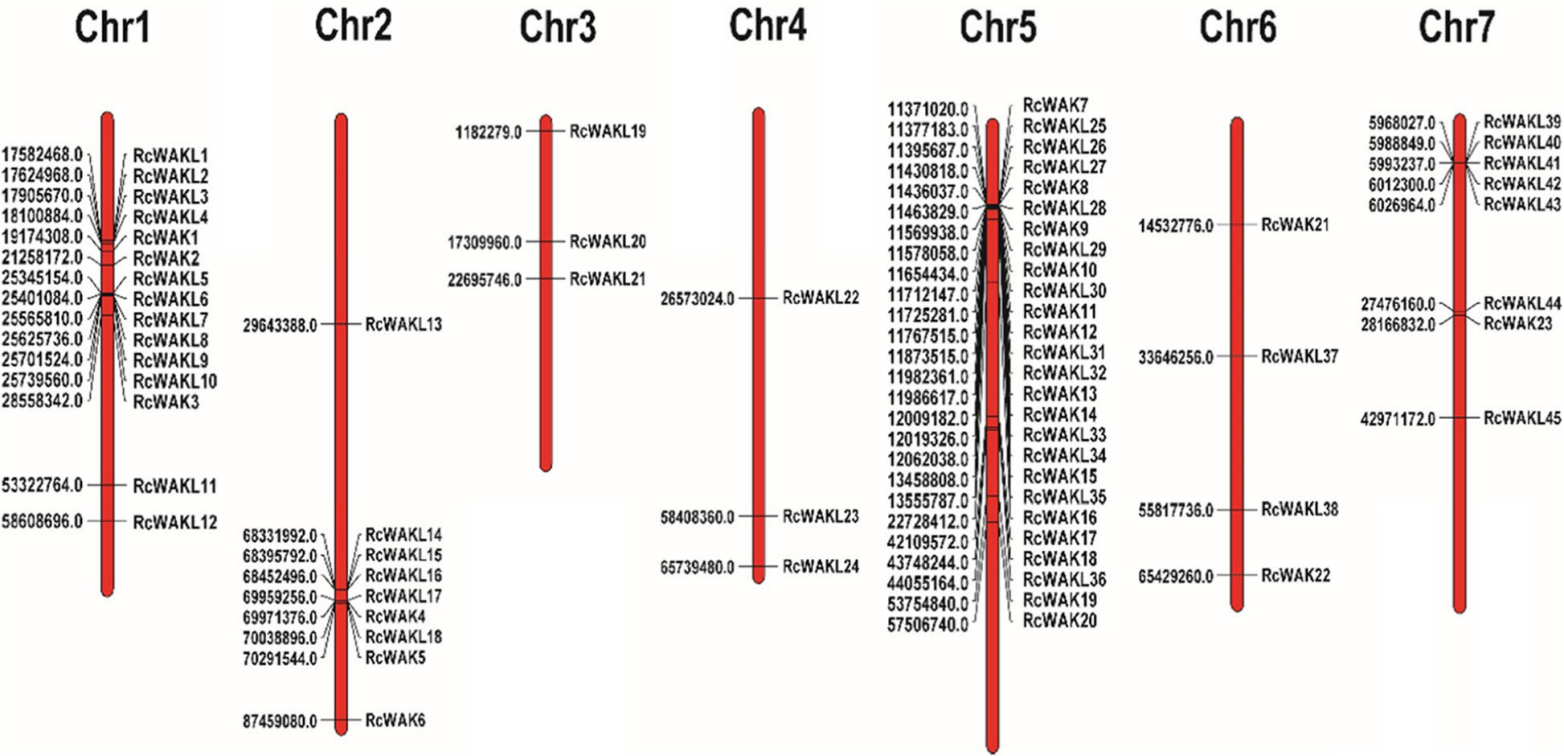
The localization of *RcWAK/RcWAKL* family members on ‘*Rose chinensis*’ chromosome. Numbers represented the gene starting position on chromosome

*RcWAKs/RcWAKLs* were mapped to seven chromosomes of the *R. chinensis* genome using Mapchat2.2. *RcWAK1*-*RcWAK23* and *RcWAKL1*-*RcWAKL45* were named according to their order on the chromosomes (Fig. 2). *RcWAK/RcWAKL* family genes were unevenly distributed on the seven chromosomes of rose (Table 1; Fig. 2). The high density of *RcWAKs/RcWAKLs* location was observed in several specific regions, such as six *RcWAKLs* (*RcWAKL5*-*RcWAKL10*) distributed at 25.34-25.74 Mb on chromosome 1 and fifteen *RcWAKs/RcWAKLs* distributed at 11.37-11.98 Mb on chromosome 5. In contrast, chromosomes 3, 4 and 6 contained only 3, 3 and 4 of *RcWAKs/RcWAKLs*, respectively. *RcWAK* members were located on chromosomes 1, 2, 5, 6 and 7, while *RcWAKLs* were located on all seven chromosomes. The unbalanced distribution of *RcWAK/RcWAKL* genes indicated genetic variation during the evolutionary process.

### Phylogenetic analysis and structure analysis of rose RcWAK/RcWAKL genes

**Fig. 3:**
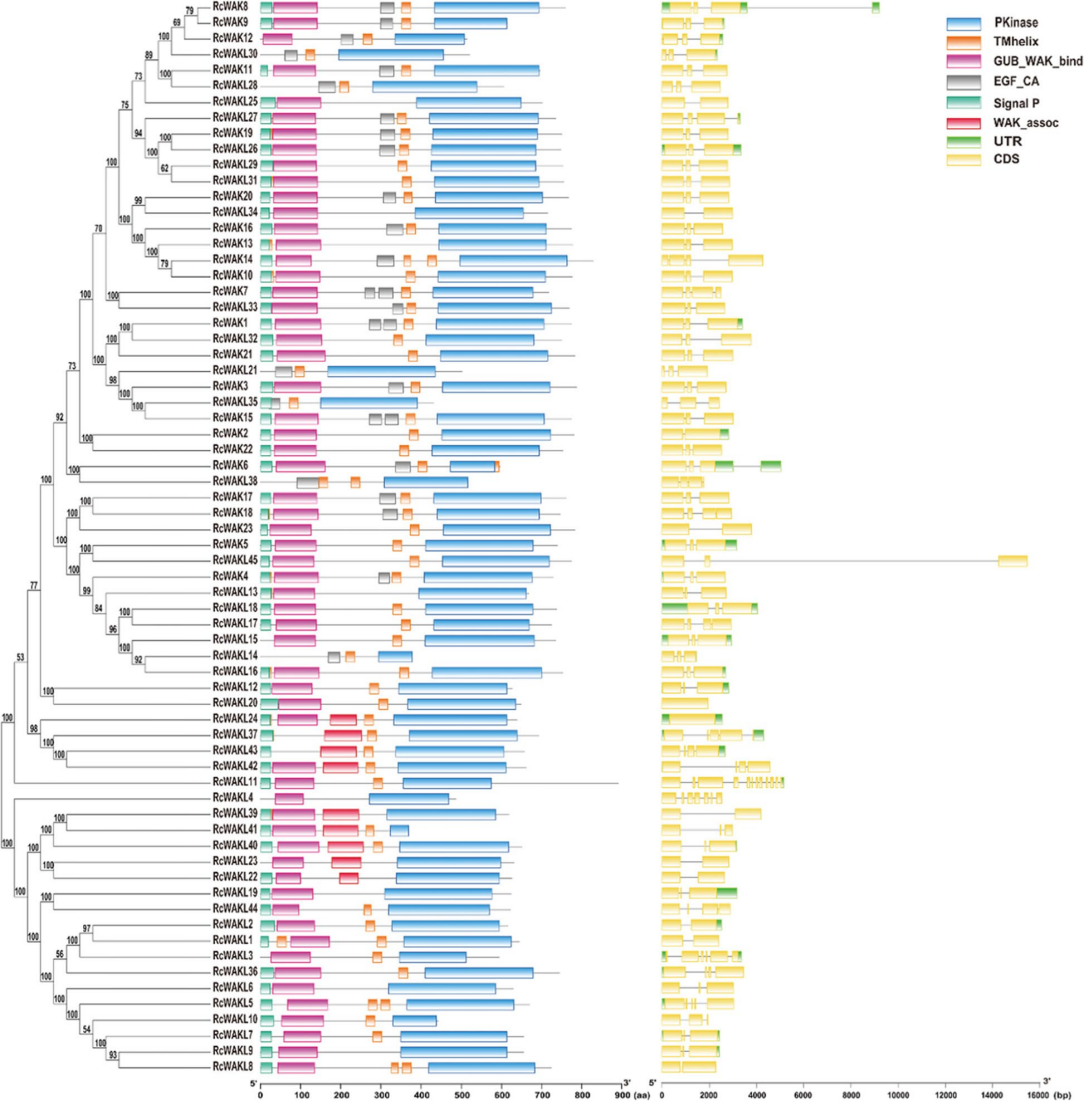
Phylogenetic analysis of RcWAK/RcWAKL family. A complete alignment was used to construct the tree with NJ method. The bootstrap value were symbolled on the branch node. RcWAK/RcWAKL gene conserved domain were analyzed by conserved domain database (CDD). Exon, intron and UTR were represented by yellow box, dark line and green box, respectively

A phylogenetic analysis of RcWAKs/RcWAKLs was performed by using neighbor-joining method (Fig. 3). The subsequent intron-exon structure analysis and conserved domain analysis of RcWAKs/RcWAKLs were consistent with the results of the phylogenetic analysis (Fig. 3). *RcWAKs/RcWAKLs* contained mostly 2-3 introns, while *RcWAKL20* and *RcWAKL24* had no introns and *RcWAKL11* contained 11 introns. Most of the genes in the same evolutionary branch exhibited similar exon-intron structures, such as *RcWAKL21*, *RcWAKL35*, *RcWAK15*, *RcWAK3*, *RcWAK21*, *RcWAK1* and *RcWAKL32* all contained 2 introns (Table 1; Fig. 3). However, there were a few exceptions, for example, *RcWAKL43*, *RcWAKL42*, *RcWAKL37* and *RcWAK24*. More than that, the intron lengths of *RcWAK/RcWAKL* family members were spanning a wide range, from tens to tens of thousands of nucleotides. *RcWAKL45* contained the longest intron (12,233bp), while *RcWAKL38* had the shortest intron (59bp).

In addition, we further analyzed the conserved domains of RcWAK/RcWAKL protein sequences by conserved domains database (CDD), and the results were similar to the previous results with Pfam database. All RcWAKs contained the highly conserved EGF_CA, GUB_WAK_bind and Pkinase, while RcWAKLs lacked the EGF_CA or GUB_WAK_bind domain. Meanwhile, the transmembrane (TM) structures and the signaling peptides (SP) were determined based on TMHMM Server (http://www.cbs.dtu.dk/services/TMHMM/) and SignalP (http://www.cbs.dtu.dk/services/SignalP/). Several RcWAKs/RcWAKLs were missing TM or SP in structure prediction. RcWAKL4 and RcWAKL23 missed both two structures (Fig. 3). Overall, RcWAKs/RcWAKLs are highly conserved in amino acid sequences from phylogenetic analysis, although some of RcWAKs/RcWAKLs lack one or two structures. RcWAKs/RcWAKLs from different evolutionary branches exist in diversity.

**Fig. 4:**
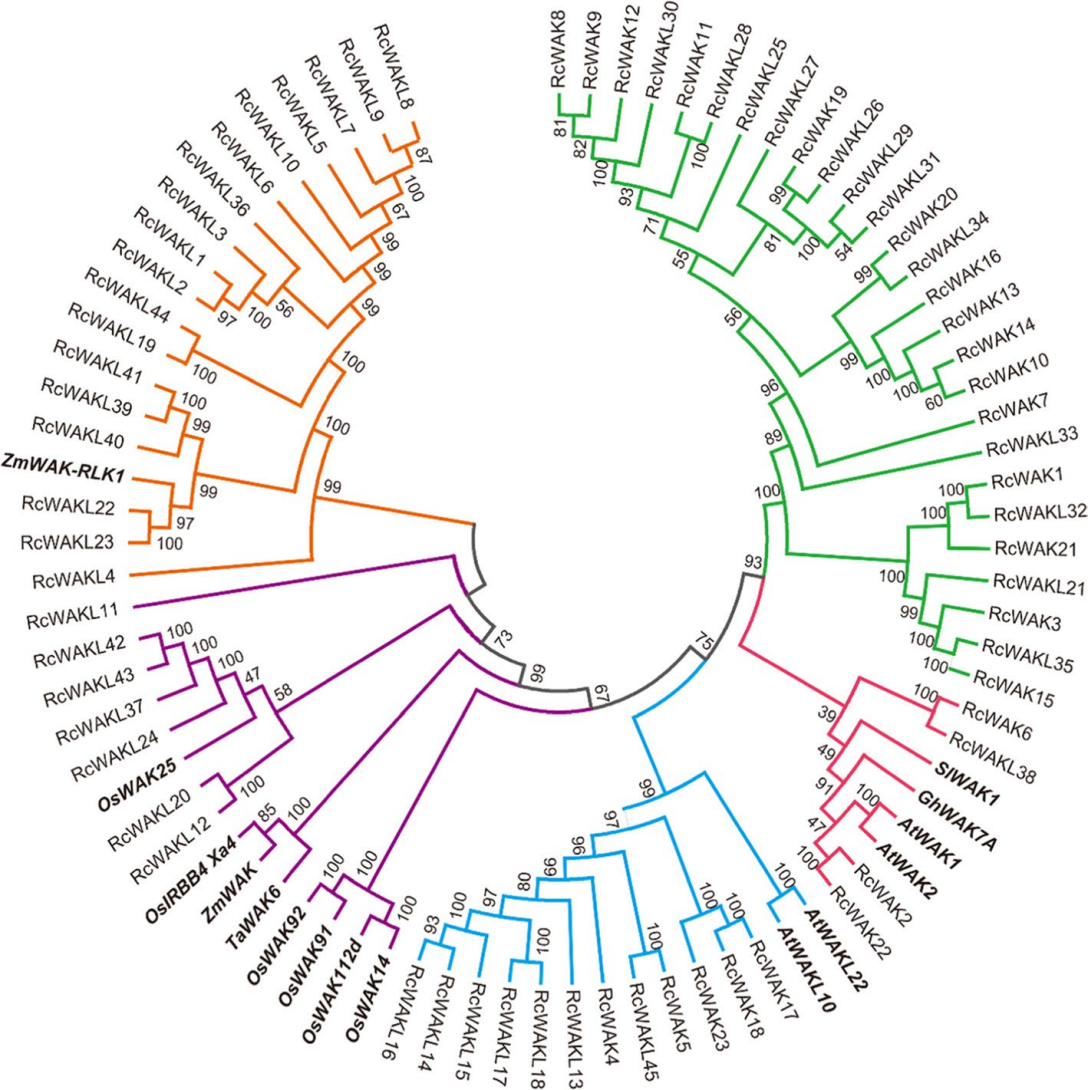
Phylogenetic analysis of the RcWAKs/RcWAKLs with defense-related WAKs/WAKLs from other plant species. Complete alignments of the rose and and the defense-related WAKs/WAKLs from other plant species, including Arabidopsis, cotton (*Gossypium hirsutum*), rice (*Oryza sativa*), tomato (*Solanum lycopersicum*), maize (*Zea mays*), and wheat (*Triticum aestivum*), were used to construct a phylogenetic tree using the Neighbor-Joining method. The bootstrap values are indicated on the nodes of the branches. The WAKs/WAKLs reported to be involved in plant disease resistance are marked in bold. Group I-V were labled in green, red, blue, purple and orange, respectively

In recent years, an increasing number of studies have confirmed that WAK/WAKL family genes played a critical role in defense response. We compiled a total of 15 plants defense response-related WAK/WAKL family genes, including *Arabidopsis*, rice (*Oryza sativa*), tomato (*Solanum lycopersicum*), cotton (*Gossypium hirsutum*)*,* maize (*Zea mays* L.), and wheat (*Triticum aestivum*) (Supplemental Table S2). To evaluate the evolutionary relationship between RcWAK/RcWAKL and the defense-related WAK/WAKL genes reported in different species, a phylogenetic tree was established using the neighbor-joining method (Fig. 4). The results showed that the proteins of the WAK/WAKL family were divided into five groups, which were labeled with different colors in Fig. 4. The groups II, III, IV and V contained WAK/WAKL from other species which were involved in plant resistance. We have also performed a comparative analysis of conserved domains in different groups. The result showed all of the five groups contained GUB_WAK_bind domain logo (PF13947) (Supplemental Fig. S1) and Pkinase_Tyr (PF07714.17) logo (Supplemental Fig. S3). However, the group V consisting of RcWAKLs was lack of EGF_CA logo, which was constituted with six cysteine (C) skeleton (Supplemental Fig. S2).

### Expression patterns of *RcWAK/RcWAKL* genes in rose petals induced by *B. cinerea*

Studies have shown that WAK/WAKL family members played a vital role in plant defense response to fungal pathogens. The *WAKs/WAKLs* which were up-regulated upon pathogen infection may involve in plant immunity. Our previous study established an RNA-seq analysis of *B. cinerea* inoculated rose petals at 30 hours post inoculation (hpi) and 48 hpi. On rose petals, *B. cinerea* conidia germinate at 24 hpi, and the early response to infection is occurred at 30 hpi, with petals having no visible lesion formation at this time point. 48 hpi corresponds to a later response, with the primary lesions starting to expand [15].

**Fig. 5:**
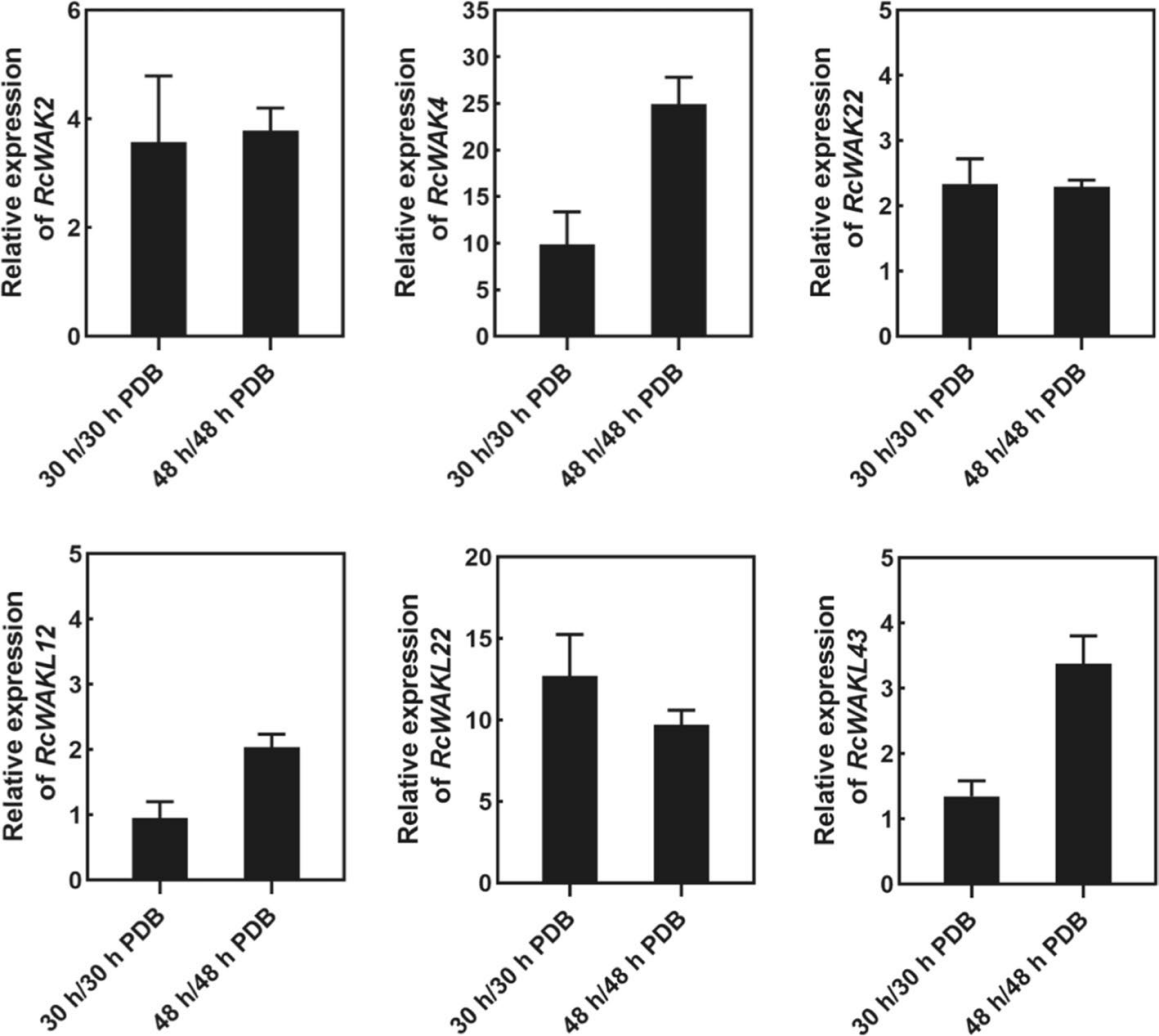
Validation of expression of selected *RcWAK/RcWAKL* genes using qPCR at 30 and 48 hours post inoculation. *RcUBI2* was used as a reference gene. The primers used for each transcript were listed in Table S1. PDB, potato dextrose broth. ‘T’ represented the standard deviation

Twelve of *RcWAK/RcWAKL* genes were significantly up-regulated expression in RNA-seq data (Table 2). Among them, *RcWAK2*, *RcWAK4*, *RcWAK8*, *RcWAK14*, *RcWAK16*, *RcWAKL6*, *RcWAKL22* and *RcWAKL43* were induced in rose petals at 30 hpi, while *RcWAK22*, *RcWAKL12*, *RcWAKL23*, and *RcWAKL43* were also induced at 48 hpi. To further clarify the expression profiles from the transcriptome data, *RcWAK2*, *RcWAK4*, *RcWAK22*, *RcWAKL12*, *RcWAKL22* and *RcWAKL43* expression patterns were confirmed by RT-qPCR (Fig. 5).

**Table 2:** Expresion of the RcWAK/RcWAKL genes under *B.cinerea* infection^a^

Gene	Accession number	Group	log_2_Ratio 30hpi	log_2_Ratio 48hpi
*RcWAK2*	RchiOBHm_Chr1g0331391	II	1.75	2.96
*RcWAK4*	RchiOBHm_Chr2g0152591	III	7.04	-
*RcWAK8*	RchiOBHm_Chr5g0016611	I	2.19	3.13
*RcWAK14*	RchiOBHm_Chr5g0017321	I	3.41	5.11
*RcWAK16*	RchiOBHm_Chr5g0028941	I	1.40	5.64
*RcWAK22*	RchiOBHm_Chr6g0306911	II	-	1.66
*RcWAKL6*	RchiOBHm_Chr1g0333351	V	1.56	4.32
*RcWAKL12*	RchiOBHm_Chr1g0368501	IV	-	1.53
*RcWAKL22*	RchiOBHm_Chr4g0405811	V	4.65	3.94
*RcWAKL23*	RchiOBHm_Chr4g0434711	V	-	3.69
*RcWAKL39*	RchiOBHm_Chr7g0185911	V	-	2.33
*RcWAKL43*	RchiOBHm_Chr7g0186001	IV	2.07	2.92

^a^The log2 transformed expression profiles were obtained from the RNA-seq dataset

### *RcWAK4* participated in rose resistance to *B. cinerea*

To further illustrate the potential role of *RcWAK/RcWAKL* in rose petals against gray mold, we knocked down the expression of *RcWAK4* using VIGS. *RcWAK4* was the most significantly up-regulated *RcWAK*s in RNA-seq data (Table 2) and our qPCR also confirmed *RcWAK4* was the most strongly induced *RcWAKs* upon *B. cinerea* inoculation (Fig. 5). Meanwhile, RcWAK4 belonged to the group III of RcWAKs/RcWAKLs and was close to AtWAKL10 and AtWAKL22, which were known to involve in plant disease resistance. Thus, *RcWAK4* was considered an important candidate gene for rose resistance to *B. cinerea*.

**Fig. 6:**
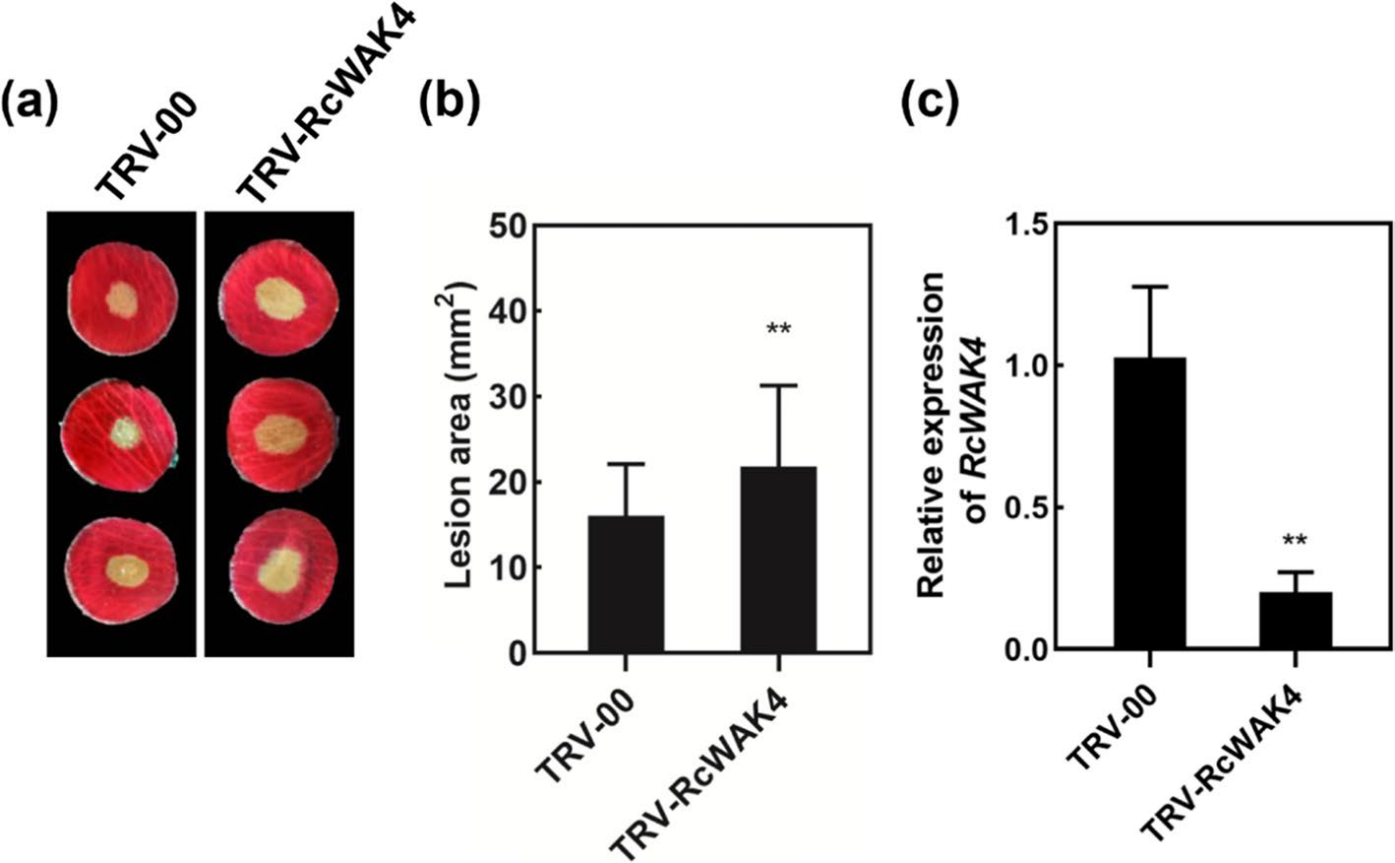
Functional analysis of rose wall-associated kinase *RcWAK4*. **a** Decreased rose petal resistance to *B. cinerea* upon silencing of *RcWAK4*, shown at 60 hours post inoculation. **b** Statistical analysis of lesion area in rose petals. The value represented the mean of lesion area from at least 48 petal discs, Error bar=standard deviation, **P < 0.01. **c** Quantification of *RcWAK4* expression in RcWAK4-silenced and control plants

To clarify whether *RcWAK4* was involved in rose petal defense response, we knocked down the expression of *RcWAK4* in petals. To this end, we cloned the coding sequence fragment 309 bp of *RcWAK4* into the *TRV2* vector to generate TRV2-RcWAK4. Mixed agrobacterium cells carried TRV2-RcWAK4 and TRV1 in a 1:1 ratio and then vacuum infiltrated into rose petal discs for *RcWAK4* silencing. The silenced petals were subsequently inoculated with *B. cinerea*. The *RcWAK4*-silencing petals exhibited a significant increase in lesion area compared with control petals inoculated with an empty TRV vector (TRV-00) (Fig. 6a and b). Finally, the silencing efficiency was confirmed by qPCR (Fig. 6c). These results suggested that *RcWAK4* played an important role in rose petal resistance to *B. cinerea*.

## Discussion

Plant resistance responses are beginning from the PRRs located on cell membrane recognizing to pathogenic signals, such as microbial-associated molecular patterns/damage-associated molecular patterns (MAMPs/DAMPs) [16, 17]. WAK/WAKL is a vital class of PRRs for necrotrophic pathogens (e.g., *B. cinerea*). Genome-wide identification of WAKs/WAKLs has been successively reported in *Arabidopsis* [18], rice (*Oryza sativa*) [19], cotton (*Gossypium hirsutum*) [20] and barley (*Hordeum vulgare*) [5]. However, there is no systematic analysis of the RcWAK/RcWAKL gene family, and the functions of the RcWAKs/RcWAKLs were largely unclear. In this study, we used ‘*R. chinensis*’ genome as a reference to perform a genome-wide analysis of RcWAK/RcWAKL gene family. We predicted the potential function of the RcWAK/RcWAKL gene family in rose through chromosome localization, exon-intron structural analysis, conserved domain, phylogenetic analysis, and gene expression pattern under *B. cinerea* infection.

The rose RcWAK/RcWAKL family contained 68 members (Table 1), while in *Arabidopsis*, rice, and barley were identified 26, 125, and 91 WAK/WAKL family members, respectively. The large disparity in the number of WAK/WAKL family members among different species may indicate that WAK/WAKL genes underwent different degrees of expansion during the evolution of plants. However, we have identified a total of 23 RcWAKs, they have a highly conservative domain with AtWAK1-AtWAK5 [18] and GhWAK1-GhWAK29 [20], including signaling peptide, EGF_CA, transmembrane and Pkinase (Fig. 3).

In phylogenetic analysis (Fig. 4), RcWAKs/RcWAKLs were divided into five groups. Evolutionary relationship analysis of barley also divided 91 HvWAK/HvWAKL into five groups [5]. The RcWAKL11, which was located in the group V, was evolutionarily distant from the other members in the same group (RcWAKL42, RcWAKL43, RcWAKL37, RcWAKL24, RcWAKL20 and RcWAKL12), probably due to more exons in the *RcWAKL11* coding sequence. *RcWAK* members were distributed on chromosomes 1, 2, 5, 6 and 7, and evolutionary analysis showed that none of the RcWAK members were divided in group V (Figs. 2 and 4). Nevertheless, *RcWAKLs* were distributed on all seven chromosomes, but none of RcWAKLs appeared in group III in the evolutionary analysis. All four groups contained plant defenses associated WAKs/WAKLs except group I. While twelve of *RcWAKs/RcWAKLs* significantly up-regulated in the RNA-seq data (Table 2) induced by *B. cinerea* were distributed in all groups. The results implied that there was no group specificity on the role of WAK/WAKL in plant defense responses.

The expression pattern of *RcWAKs/RcWAKLs* induced by *B. cinerea* provided abundant candidate genes for its possible involvement in rose defense response to gray mold. The differential expression of *RcWAK4* was more than 7-fold at the early stage after *B. cinerea* infection (Table 2), and the qPCR results were also consistent (Fig. 5). Transient silencing of *RcWAK4* in rose petals exhibited increased susceptibility to *B. cinerea* (Fig. 6). In phylogenetic analysis, AtWAKL10 and AtWAKL22 were in the same group with RcWAK4 (Fig. 4). AtWAKL10 as a candidate guanylyl cyclase (GC), involved in *Arabidopsis* defense response [21]. Together, these results suggested that *RcWAK4* as an important RcWAKs/RcWAKLs involved in the response of rose against *B. cinerea*.

## Conclusions

A genome-wide characterization of RcWAK/RcWAKL family was performed in this study, mainly including chromosome localization, gene structure analysis, phylogenetic relationships, gene expression analysis induced by *B. cinerea*. We have identified a total of 68 non-redundant RcWAK/RcWAKL family members in the whole genome of *R. chinensis. RcWAK4* was confirmed to be involved in rose petal resistance to gray mold by VIGS. These results provide a piece of essential information for further study RcWAK/RcWAKL family members in rose and may facilitate the further breeding of disease resistance roses.

## Methods

### Identification of the WAK/WAKL genes in rose genome

The complete rose (*Rosa chinensis* ‘Old blush’) genome was used as a reference and the data were downloaded from https://lipm-browsers.toulouse.inra.fr/pub/RchiOBHm-V2/ [22]. Firstly, we performed a BlastP homology search in rose protein library based on *Arabidopsis* WAK/WAKL family members. Secondly, based on the definition of WAK/WAKL, the HMM files of EGF_CA (PF07645.15), GUB_WAK_bind (PF13947.6) and PKinase_Ser/Thr (PF07714.17) were download from Pfam database (http://pfam.xfam.org/) for hmmsearch with E-value <1e^-3^. Finally, combining the predictions with SignalP (http://www.cbs.dtu.dk/services/SignalP/), TMHMM Server (http://www.cbs.dtu.dk/services/TMHMM/) and Conserved domain database (https://www.ncbi.nlm.nih.gov/cdd/?term=), the candidate RcWAKs/RcWAKLs containing both SP, EGF_CA or GUB_WAK_bind, transmembrane structures and PKinase structural domains were selected. Gene chromosome location distribution was mapped by Mapchat2.2 [23].

### Gene structure and phylogenetic analyses

Extracted coding sequences region of *RcWAK/RcWAKL* genes, and the gene intron-exon structure was mapped using TBtools [24] with the genome-wide gene information file as reference. The conserved domain information of RcWAK/RcWAKL genes were obtained from Pfam database and Conserved domain database (CDD, https://www.ncbi.nlm.nih.gov/Structure/cdd/wrpsb.cgi). Full sequence alignment of RcWAK/RcWAKL was performed using the ClustalW method. Phylogenetic analysis of the comparison results was constructed using Neighbor-Joining of MEGA6.0 [25] with a bootstrap of 1000 replicates.

The WAK/WAKL from other species, containing *Arabidopsis*, rice (*Oryza sativa*), tomato (*Solanum lycopersicum*), cotton (*Gossypium hirsutum*)*,* maize (*Zea mays* L.), and wheat (*Triticum aestivum*), which were related to plant defense response were used to phylogenetic analysis with RcWAKs/RcWAKLs. The protein sequences were collected from Genbank. All the WAKs/WAKLs were aligned by ClustalW before phylogenetic analysis with NJ of MEGA6.0 [24] (bootstrap test=1000 replicates).

The comparative analysis of conserved domains between different groups of RcWAKs/RcWAKLs. The group menbers of RcWAKs/RcWAKLs were aligned by ClustalW before performing sequences logo on WebLogo3 (http://weblogo.threeplusone.com/).

### Expression data of *RcWAKs/RcWAKLs* in rose petal induced by *B. cinerea*

The RNA-seq data of rose petals infected by *B. cinerea* were obtained from the National Center for Biotechnology Information (NCBI) database with the accession number PRJNA414570 [15]. There no special permissions are necessary to collect these data from NCBI. Clean data were mapped to ‘*Rosa chinensis*’ genome, and reads per kb per million reads (RPKM) were used to value gene expression levels, and log2 (RPKM treatment/ RPKM control) was used to calculate the differential expression levels of genes.

The real time quantitative PCR were performed to confirm the RNA-seq results. To this end, cDNA synthesis were according to the RT Super Mix kit (Vazyme). qPCR was performed with TB Green Premix qRT-PCR kit (TAKARA) based on One Plus real-time PCR system (Applied Biosystems). *RcUBI2* was used as the housekeeping gene, and the primers used in the experiment were shown in Supplemental Table S1.

### Virus induced gene silencing (VIGS) and *B. cinerea* inoculation assays

The CDS sequence of *RcWAK4* was used as a reference sequence to design specific primers VIGS-RcWAK4-F/R. The target was amplified using rose petal cDNA as a template to obtain 309 bp fragment. The fragment is constructed into the tobacco rattle virus (TRV) vector [26].

The VIGS and *B.* cinerea inoculation was performed as previously described [27]. Briefly, petals were detached from the outer whorls of the rose, which was at the opening stage 2 [28]. A 15-mm disk was punched from the center of each petal. Constructs expressing *TRV1* and recombinant *TRV2* cultured by *Agrobacterium tumefaciens* were mixed in a 1:1 ratio and vacuum-infiltrated into the petal disks. Six days after the TRV infection, the petal disks were inoculated with 2 μL *B. cinerea* with the concentration of 1x10^5^ spores/mL. The VIGS was repeated at least three times using at least 48 discs. After the *B. cinerea* inoculation, the lesion area was measured by ImageJ, and a Student’s *t*-test was conducted to identify any significant differences. All the constructs and plant materials used in this study are freely available from the corresponding author, for research purposes. There no special permissions are necessary to collect item.

## Supplementary Information

**Additional file 1: Supplemental Table S1.** List of primers used in this study

**Additional file 2: Supplemental Table S2.** Plant *WAK*/*WAKL* genes involved in disease resistance [29–35]

**Additional file 3: Supplemental Figure S1.** Comparative analysis of GUB_WAK domain between different groups of RcWAKs/RcWAKLs

**Additional file 4: Supplemental Figure S2.** Comparative analysis of EGF_CA domain between different groups of RcWAKs/RcWAKLs

**Additional file 5: Supplemental Figure S3.** Comparative analysis of Pkinase_Try domain between different groups of RcWAKs/RcWAKLs

**Additional file 6: Supplemental Data.** WAK/WAKL protein sequences

## Data Availability

The protein sequences used and/or analyzed during the current study are available in ‘*Rosa chinensis*’ genome (https://lipm-browsers.toulouse.inra.fr/pub/RchiOBHm-V2/) and Genebank (https://www.ncbi.nlm.nih.gov/genbank/). The RNA-seq data used in the current study is available in NCBI database with the accession number PRJNA414570. The plant materials are available from the corresponding author on reasonable request.

## Abbreviations

hpi: hours post inoculation
NJ: neighbor-joining
RPKM: number of reads per kb per million reads
HMM: Hidden Markov Model
CDD: Conserved Domains Database
VIGS: virus-induced gene silencing
