# Supplementary material for: Characterization of wall-associated kinase/wall-associated kinase-like (WAK/WAKL) family in rose (Rosa chinensis) reveals the role of RcWAK4 in Botrytis resistance
Source: BMC Plant Biol. 2021 Nov 10;21:526. doi: 10.1186/s12870-021-03307-9 (PMC8582219; doi:10.1186/s12870-021-03307-9)
Supplement: Supplementary file 1 — Additional file 1: Supplemental Table S1. List of primers used in this study [file 12870_2021_3307_MOESM1_ESM.docx]

**Supplemental Table S1. List of primers used in this study**

| **Primer name** | **Primer sequence（5’-3’）** |
| --- | --- |
| qPCR-RcWAK2 | F：CACATTACCAGTTGATGATGGGAGA |
|  | R：GCCTAGCAAGACAATCACAGAGACG |
| qPCR-RcWAK4 | F：CGTCCTACCAGCCAACTTGAGCGG |
|  | R：GGAGAGCATCATCCAGGTGATTCC |
| qPCR-RcWAK22 | F：CAAGCAAGTTGAAGGCAAAATGATA |
|  | R：GCATCGGTATCCAGACCCATTTTCG |
| qPCR-RcWAKL12 | F：CAGCCATCATTGCCATTGTTCTTTA |
|  | R：GGATTGCTTCTCGCTCTTTGGTCAG |
| qPCR-RcWAKL22 | F：AACCACACTCTTCACTTGTCACACGAT |
|  | R：CCATTTGGCGAGGTAAAGTTTTCTAAA |
| qPCR-RcWAKL43 | F：TACAACTGTTCCCAAGAACCTGTGA |
|  | R：ATGGTAGCAAAAGAATGAAGTGTGG |
| qPCR-RcUBI2 | F：GCCCTGGTGCGTTCCCAACTG |
|  | R：CCTGCGTGTCTGTCCGCATTG |
| VIGS-RcWAK4 | F：CAACTCTTCAAATACGTGGAACAGC |
|  | R：TCTTTATGACTTTGTGTAACCCCCA |
