# Supplementary material for: Characterization of wall-associated kinase/wall-associated kinase-like (WAK/WAKL) family in rose (Rosa chinensis) reveals the role of RcWAK4 in Botrytis resistance
Source: BMC Plant Biol. 2021 Nov 10;21:526. doi: 10.1186/s12870-021-03307-9 (PMC8582219; doi:10.1186/s12870-021-03307-9)
Supplement: Supplementary file 2 — Additional file 2: Supplemental Table S2. Plant WAK/WAKL genes involved in disease resistance [29–35] [file 12870_2021_3307_MOESM2_ESM.docx]

**Supplemental Table S2 Plant WAK/WAKL genes involved in disease resistance**

| **Gene name** | **gene ID** | **Species** | **Pathogens** | **References** |
| --- | --- | --- | --- | --- |
| AtWAK1 | AT1G21250 | *Arabidopsis thaliana* | *Botrytis cinerea* | [8] |
| AtWAK2 | AT1G21270 | *Arabidopsis thaliana* | no typical one | [26] |
| AtWAKL10 | NP_178086.1 | *Arabidopsis thaliana* | *Pseudomonas syringae/Botrytis cinerea* | [27] |
| AtWAKL22 | AT1G79670 | *Arabidopsis thaliana* | *Fusarium oxyporum* | [28] |
| GhWAK7A | Gh_A02G0727 | *Gossypium* spp | *Verticillium dahlia/Fusarium oxysporum* | [29] |
| OsWAK14 | Os02g42150 | *Oryza sativa* | *Magnaporthe oryzae* | [10] |
| OsWAK91 | Os09g38850 | *Oryza sativa* | *Magnaporthe oryzae* | [10] |
| OsWAK92 | Os09g38910 | *Oryza sativa* | *Magnaporthe oryzae* | [10] |
| OsWAK112d | Os10g10130 | *Oryza sativa* | *Magnaporthe oryzae* | [10] |
| OsWAK25 | LOC_Os03g12470 | *Oryza sativa* | *Xanthomonas oryzae* pv. *oryzae* /*Magnaporthe oryzae* | [30] |
| OsIRBB4_Xa4 | AQQ72921.1 | *Oryza sativa* | *Xanthomonas oryzae* pv. *oryzae* | [31] |
| SlWAK1 | Solyc09g014720 | *Solanum lycopersicum* | *Pseudomonas syringae* | [32] |
| TaWAK6 | ALJ11037.1 | [*Triticum aestivum*](https://www.ncbi.nlm.nih.gov/Taxonomy/Browser/wwwtax.cgi?id=4565) | *Puccinia triticina* | [33] |
| ZmWAK | KM974808 | *Zea mays* L. | *Sporisorium reilianum* | [9] |
| ZmWAK-RLK1 | [XP_020398351.1](https://www.ncbi.nlm.nih.gov/protein/XP_020398351.1?report=genbank&log$=protalign&blast_rank=1&RID=851WGU1V013) | *Zea mays* L. | *Exserohilum turcicum* | [34] |
