# Supplementary figures and images for: Characterization of wall-associated kinase/wall-associated kinase-like (WAK/WAKL) family in rose (Rosa chinensis) reveals the role of RcWAK4 in Botrytis resistance
Source: BMC Plant Biol. 2021 Nov 10;21:526. doi: 10.1186/s12870-021-03307-9 (PMC8582219; doi:10.1186/s12870-021-03307-9)

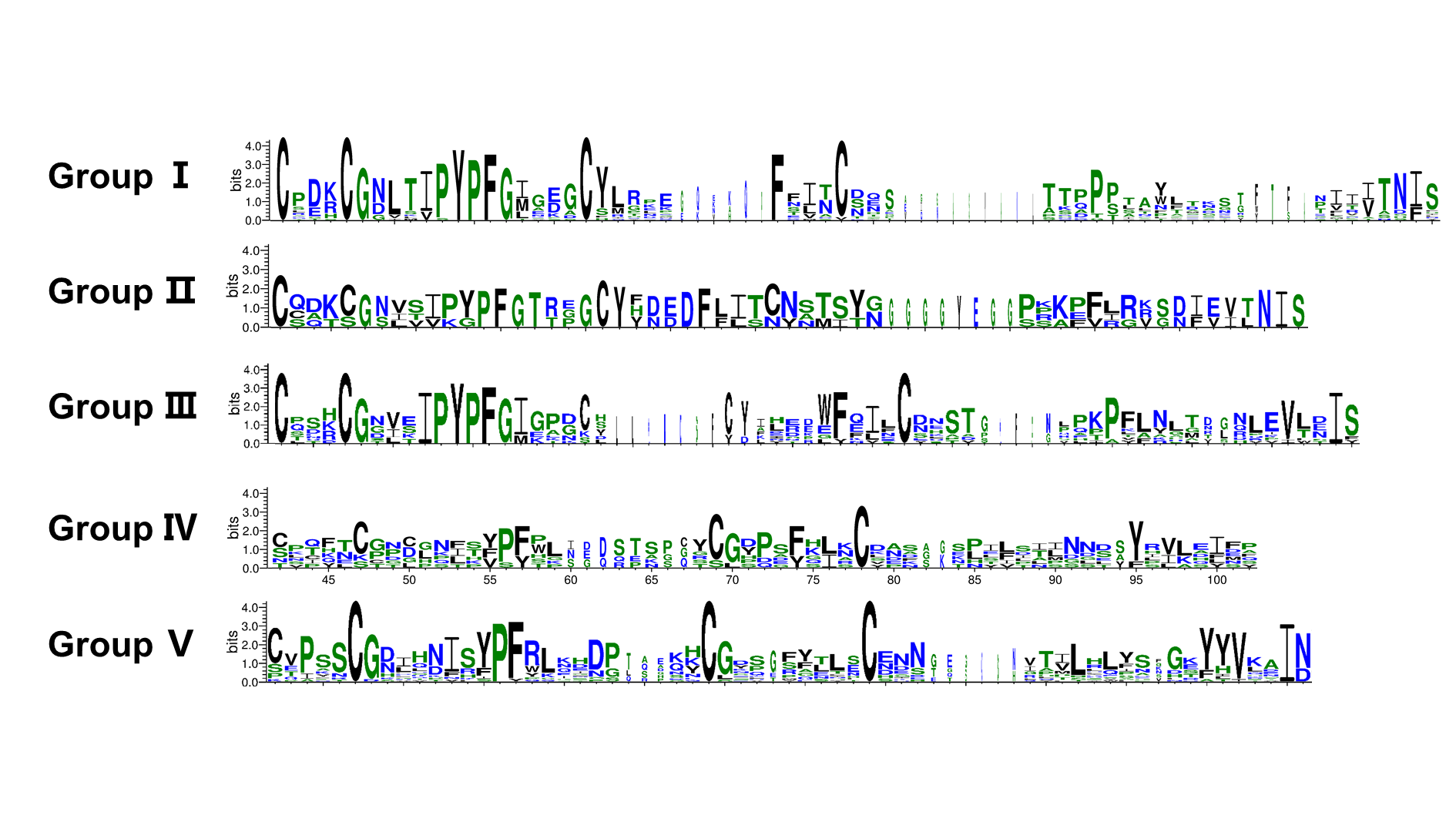

Supplement: Supplementary file 3 — Additional file 3: Supplemental Figure S1. Comparative analysis of GUB_WAK domain between different groups of RcWAKs/RcWAKLs [file 12870_2021_3307_MOESM3_ESM.tif]

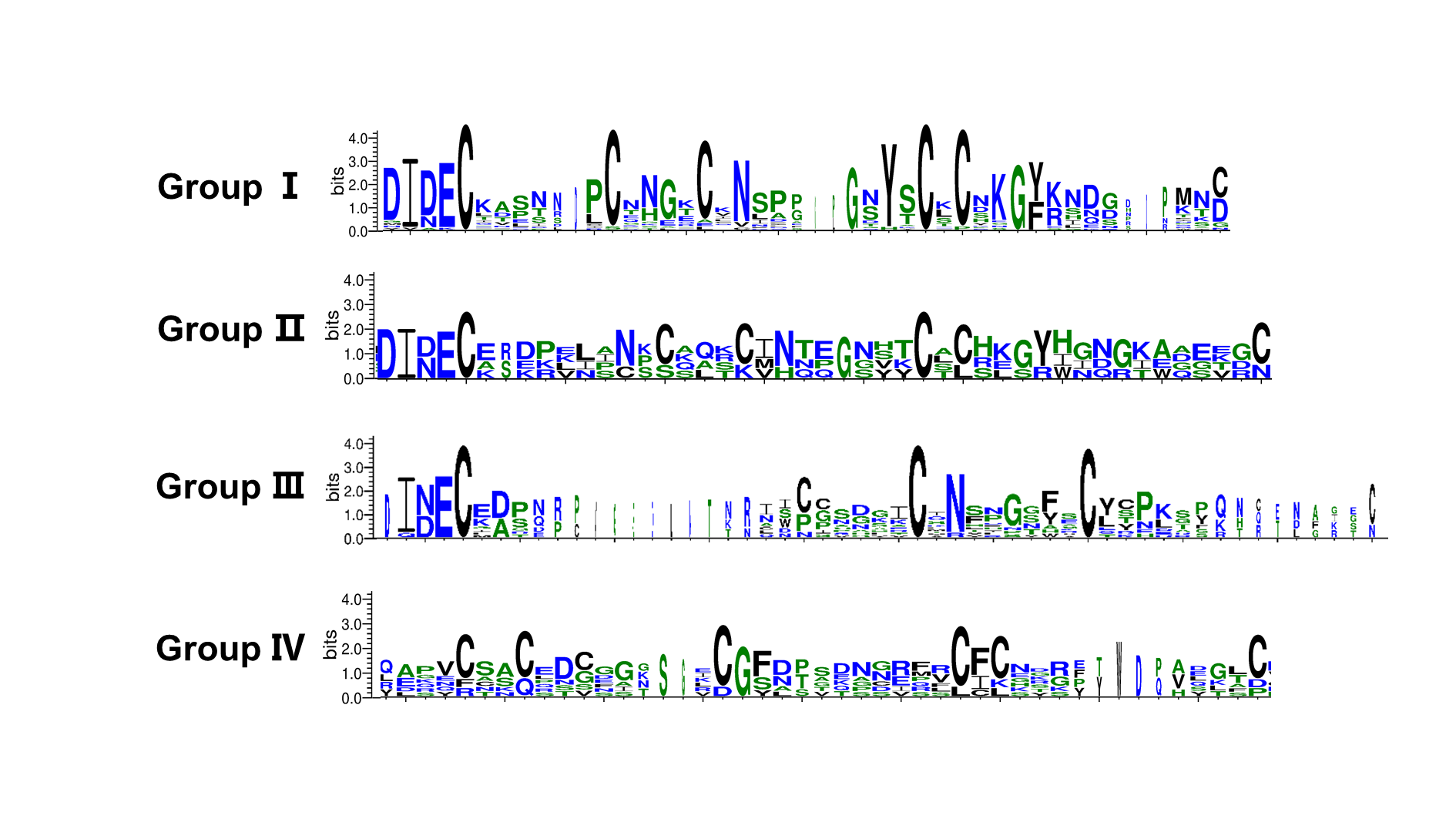

Supplement: Supplementary file 4 — Additional file 4: Supplemental Figure S2. Comparative analysis of EGF_CA domain between different groups of RcWAKs/RcWAKLs [file 12870_2021_3307_MOESM4_ESM.tif]

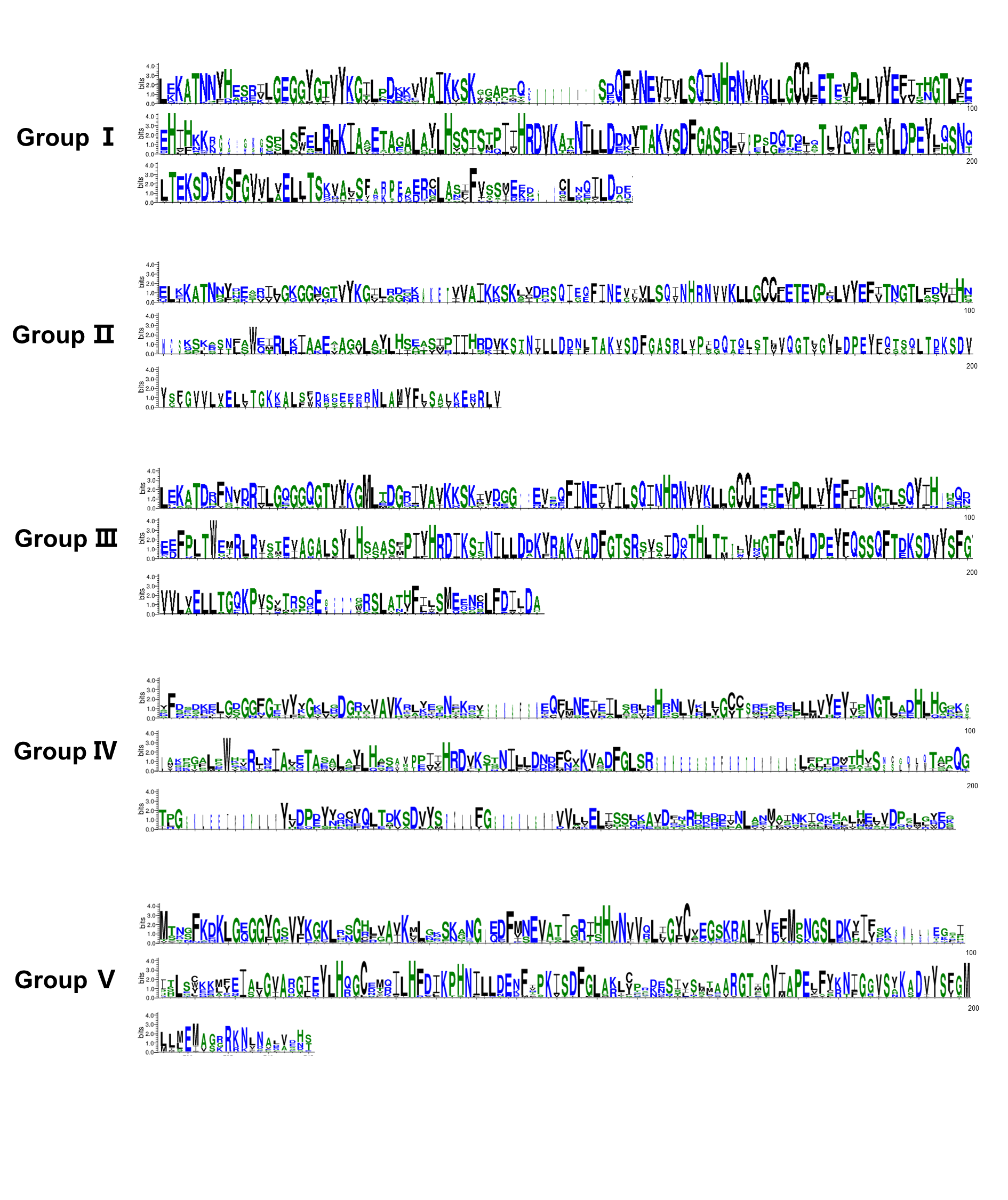

Supplement: Supplementary file 5 — Additional file 5: Supplemental Figure S3. Comparative analysis of Pkinase_Try domain between different groups of RcWAKs/RcWAKLs [file 12870_2021_3307_MOESM5_ESM.tif]
