# Supplementary material for: Characterization of wall-associated kinase/wall-associated kinase-like (WAK/WAKL) family in rose (Rosa chinensis) reveals the role of RcWAK4 in Botrytis resistance
Source: BMC Plant Biol. 2021 Nov 10;21:526. doi: 10.1186/s12870-021-03307-9 (PMC8582219; doi:10.1186/s12870-021-03307-9)
Supplement: Supplementary file 6 — Additional file 6: Supplemental Data. WAK/WAKL protein sequences [file 12870_2021_3307_MOESM6_ESM.docx]

>RcWAK1

MALVVHGKMLLMKRSLVLLAAATTLLADKTPSPPQAKPNCPDRCGNLSIPYPFGIGEGCY

LPLKGKKKQFELACNYSTKPPSLTWPTDRSASISGFHLAEGELEVMNSISKDCYNKSGFN

VEEYFSLSWSPPFSLSGRKNSFVAIGCDTSAAFLGFRGQKQIMTGCMSVCLDINTVDLNS

CTGVGCCQTKIPDELNNLTVRLDSYFNHSYVREFNPCSYAFVVKDGYFNFSGTRSFKELK

NMEQVPLIGNWQIGNETCDVAKKNAVSYACKANSVCVNRSKSPWPAGYYCQCSPGYEGNP

YIGCRDIDECLAEINPCGNGTCKNIPGSYSCKCKKGFKSDGPFKCIARPTANNSALKISL

GICIGFFVLLVVIFGLYREYKRRQFNEMKKKYFEANGGPKLQLQLKQLASQKEPLAVQIF

TEEELKKATKNYDENEKLGEGGYGIVYKGVLDKRVVAIKISKMTAQVESDQFINEVIVLS

QISHRNVVRLLGCCLETKTPMLVYEYVGSGTLYDHIHKKKGQLLSFAQRLKIAAETATAL

SYLHHSTTTQIVHRDVKATNILLDEKLMAKVSDFGASKLIPDDKTQLSTLVQGTLGYLDP

EYLQSNTLTEKSDVYSFGVVLVELLTSENVIRFDKTEAERNLANVFVSTIEKKGLRALGP

ILDDEIVKDGNWEIIEKVANLAQRCLSVKGDERPTMKEIERELDEIRKTSAKQPDGKKLY

ASSSKETDPSLESHSDAYNVEISDEGDGGSTGIISSTEYDASMQNQAQKSSSYGR

>RcWAK2

MSATIKKIFLLRLVLVAIEVLLVSSQTVDITREGCQDKCGNVSIPYPFGTREGCYYDEDF

LITCNSTSYNPSKPFLRVSDIEVTNISIDGKLYITQDIARACFTKSGAPYYGNYNPYLSL

KKFYISDTDNKFTGVGCDTYAEIKGYQGISLMYTGGCFSICQSTASISNYSCSGIGCCQT

PIAKGMTYFTVGVGSYNNQTEVWNFNPCSYAFLVQEGKFNFTSDMLSDLRNVSQFPVVLD

WSIGNETCSQIKNNACKGNAKCLDVKNGLGYRCKCNEGYEGNPYLNGCTDINECKDPVIA

NKCKQRCINTEGNYTCACHKGYHNNGTADSVDCIADHQTIFVQIILGKFNSNTLLHLVTK

NTRTTNAWMCVGNVGIGVGLISLLICISWLYLVYKRWKLMKLKEKFFIQNGGLMLKQQLS

ERQGGASNQTAKIFTEDQLKKATNHFSEARIVGKGGFGTVYKGIIADERAKETVVAIKKS

KLVDRSQIEQFINEVVVLSQINHRNVVKLLGCCFETEVPLLVYEFVTNGTLFDYIHNKSK

ESNFAWEIRLRIAAETAGVLSHLHSEASIPIIHRDVKSTNILLDDNLTAKVSDFGASRLV

PSDQAQLSTVVQGTVGYLDPEYLQTSQLTDKSDVYSFGVVLVELLTGKKALSVDKPEKER

NLAMYFLSALREDRLVEVIDGSVINGANVEQLKEVSNLAKRCLKVEGKERPTMKEVAMEL

EALRRMVMHPWVSNDESNAEENEHLLDEITLETFSHGGGGDTSSGYDTMRNPFTLPVDDG

R

>RcWAK3

MVCLHGRMLLLQLSLAAVLLAYAATTMLAAAQAPPECNESCGGVSIPYPFGLSDGCYLYV

PGQVAQPFKITCDNSTSQPSLKFLDSYYFPTNITNISVGESELQVMVTTSRNCYSNNSYN

SSLYRDSVLDLPPSFTISDKNKVFSVGCNKVSMFKGFLLKVTDPEQPFAVGFSGVSLCQD

EFGKRFPETCTGFGCSGNPIPSGLQNITVAVWTVGNGLGATDQWGLSYPCSYGFVVDERN

FTFAGNQSFNELNGNRQELPVLANWAIGNDNCEAAKKNNETAFACKGVNSKCVDRAGGYF

CQCETGYAGNPYLLNDCLDINECKNLTLCSDHATCINSIGSYTCKCDKGYRNDDNDRNSC

VQIKETSSKNDKEMQISLGVCLSFLVMLIITFWIYCGMKRRKFKQLKEKYFKDNGGFLLQ

QKLENFEGPQAAKIFTREELKKATNNFDDSRKIGEGGYGFVYRGTLPDKKEVAIKVSKSN

APMTQSNQFINEVIVLSQINHRNVVRLLGCCLETQTPLLVYEFISNGTLYEHIHKKNGKG

PLSFGLRMKIAAETAGSLAYLHYSTSMQIVHRDVKATNILLDDNFTAKVSDFGASKLVPE

DQNQLSTLVQGTLGYLDPEYLQSNTLTEKSDVYSFGVVLVELITSQVAISYKKPEAERNL

ANFFVTSVEENRLDQILDLEIIKEGSFEIAEQVAHLAKRCLSLKGGDRPTMKEVAMELEG

ILQVMAKHPEGKPDASPKETDYLLAMSPANAYVVDVRGDEGEVITSIDYDQSMQIQAQIM

KPDDGGR

>RcWAK4

MVPVLFLLHVITVFLWSMSTIAAQPVASEALPIAKPNCTQDCGDVKIPYPFGIEAGCYID

DWFKIFCYKPTGKFAGQPTAYLNPANLNLVVFNISLEAGTIQVTTNFTFSSLGCVDQRTA

VLPANLSGSPFLYSEKNKFTVIGCGGITWMMLSNGSKGGGCLATCDGSDPAQREFLDGCT

GINSCCQTNIPPNLTAFNYSYQAIDTTYPGNDSCKKAFLVNPDWVGYSSGGMVSIKQLAI

PMLLDWRLSNYSTLQIRGTADWANNSGTTCTNASLCSCSSGFQGNPYLPNGCQDINECED

SDPCPGASICINVRGSFRCQSPDQTVKLAIIVVGSVLGALLILICAWGLHKVIKKRINTK

RKEKFFKQNGGLLLEKQSSGDVNVEKIKLFNSEELEKATDNYNANRILGQGGHGTVYKGM

LADGSTVAIKKSKIVDEGEVEQFINEIVILSQIIHRNVVKLMGCCLETEVPLLVYEFIPN

GTLSQYIHHQNEEFPLTWRVRLRVSIEVAGALSYLHSATSFPIYHRDIKTSNILLDDKFR

AKVADFGTSRTVTIDKTHLTMSQVQGTFGYLDPEYFQSSQFTDKSDVYSFGVVLVELLTG

QKPVSQTRPEEWRSLTNYFLLSMEENRLLDILDARVMNEGGKEEIMAVAQLARRCLNMKG

KKRPTMKEVAVQLEGIFQLSAKDSDVHTNSGEVEYVLNGWHVGSTSARYNTIHIGSGSPS

DVEPLIYVS

>RcWAK5

MQSLVHHMISIMVLCLWSLSTTPAVAAESGTALPIAKPNCQEKCGNVSIPYPFGIGPDKD

CYLEEWFEIDCNQSTRHKPFLRLTQQEVLSISIKDGTLQVNSPVTFFCNVTGSSQPANLT

GSPFVYSQRQNRFTAATCGFVSLVSSDQSVVGGCRSICDKQNTGRYSDGCDIGTNCCQTS

IPPYLRVVTASVMVDGPANMTGRNDSGKPDCSDYAFLVDKDWFETASNYSAIRNMLHVPV

ALEWSVINDTNSSSAFQGNKKCDFDTSLALNRSVLSCRCPQGFDGNPYLLQPCQDIDECI

DGTKRCWPGSKCMNLPGSFKCEDEKDTIKLALAGLGSSLGLLLLLIAAWWVHKLVKKRKS

IKRKEMSFKRNGGLLLERQLSSGEVNLEKIKLFKSKELEKSTDNFHVDRILGKGGQGTVY

KGMLTDGRIVAVKRSTKVDEGRLSQFINEVVILSQINHRNVVKLLGCCLETEVPLLVYEF

IPNGSLSQYIHEQHEDFPLTWELRLRIATEIAGALSYLHGAASIPIFHRDIKSTNILLDE

KYRAKIADFGTSRSVAIDQTHLTTLVYGTFGYLDPEYFQSSQFTDKSDVYSFGVVLVELL

SGQKPISVTRSQEEGRSLATHFIILMQDDRLFEVVDARVLNEASKEDILLVANLARRCLH

LNGRNRPTMREVTAELEGIQMSQKTTNGEQNYEEVDIVRYDSIEPWDVASTSTGTVTGTR

SGVAAGPSSSPSQGIALLSL

>RcWAK6

MSMRIKLVLVVVVIILLEHYNHGEKAAADTNPNQLELVKPGCSATCGSLVVPYPFGTTPG

CYFNDDFFISCNNTITGGGGGYEGGPKAFFRGRSDFVILNISLERQEMRVSRSISKSCLE

VDKTGNVTSYSNEDNNPGVIAPKAFRISSRRNKFTTVGCNLFGLIQTVDQTISSSPGSKK

YSSETSALRGYIDACVSTCTLRIENVKNGTCHPHGGCCQTPIAIGDINHYTTSLDTISED

FRSVKLCGYAFVTEEEEFKFSSLDLIKSLDNRKAAPLVLDWAVRNHLNCAEARSRKDYAC

KAKYSDCLNSTNGPGYICKCRKGYQGNPYLLHGCQDINECASEKLNPCSSSATCINNPGG

VTCTCSEGYIGDGKEAGTGCTHQFSRQLKLLVIAFGICMGILVLVTVGFWIYLVEKRRRL

IKLKRMFFEQNGGILLQQHLNLSRHKPSIEIMKIFSSDELIKATNNYDKSNILGQGGNGT

VYKGVLRGNKIVAIKRSKACDRSQIGEFINEGIVLSQVNHRNVVKLLGCCLETEVPVLVY

EFITNGTLASHLHPNQSQPLSITLSWQMRLKIAAEIAGALAYLIIHIYMRIIISKIL

>RcWAK7

MAFLQKMLILQLSVFSVILTTATPAVGQALPGCPDRCGDLLIPYPFGIAEGCHLGEEFFI

NCTTEVIGTSPTPYLAGTEIPVSNIFLDQGELQIMQFVARDCYDSQGYLDKKLSKVRILS

LNPPFTISGTKNKFFAVGCDTYAIFEGYRGHERYITGCMSFCESLGSVNESCSGIGCCQT

SIPTGLQNRTVKMNSYYNHAFIWDFNPCSYSFIVEDGQFEFSSKSFQELDRTSRLPMVLN

WQIGNETCDEAQKKQGYACKGNSTCVNPINLSGYFCQCLPGYEGNPYLPDGCHDINECKN

SNICSEGACVNFPGTYACVCPKGFEGDGRKAGTGCRKDNPMNPHQTSRLLIISLGMSVAF

LFIMVGSSWTYLGVKKRKFIQLREKYFKENGGLLLQQKLANHEGAVQTTKIFTAEELEKA

TNNYHEDRVVGEGGFGTVYKGILADGKVVAIKKSKISAPSQSEQFVNEVIVLSQVNHRNV

VRLLGCCFETPVPLLVYEFITNGTLFEHIHNNKGEKSPLPWELRLKIAAEIAGALAYLHS

SISMPIIHRDVKATNVLIDDNYTAKVSDFGASRLVPLDQTQITTLVQGTLGYLDPEYFHS

NQLTEKSDVYSFGVVLVELLTSKLALSFTRPEAERNLACFFERPTMKEVSMELEGMRISA

KHPWGKTGFSPEDTEHLLGSPSFSHVRSDCGPNSATISAASVYESMRMEMLMAYDNGR

>RcWAK8

MATFQGILLIIQFSFVIIGVLIVSTTAAAPQALPNCPDKCGNLTVPYPFGMAEGCYRGNA

FFVNCSQATDPPVAYLTKNGTVIITNISFAAGELQISTFIARDCYSSSALRTRRNSPVLR

LPSPYTISDTKNKFIAVGCDTYAIFKGFRGTEQSITGCMSVCNSLESVDQNSCSGVGCCQ

TNIPSGLHNRTVTLDSYSNHSGIWDFNPCSFAFIVEETQFKFSGNTSFQQLNSTTRLPMI

LNWAIGDEPDPCDVAQKRQDFVCKQNSKCVNPTNRNGYICQCLEGYEGNPYHPDGCQDID

ECKDPNLCNHGECKNLAGSYTCLCSKGYKLDSMNDKSCIKENPKNNSKMTLLLIISLSVS

IDLLVLFVGISWICCGIKRRQYTKLKEKYFKENGGLLLLQQLASHGGTVETTKIFSTEEL

EKGTNNYHESRILGEGGYGTVYKGILPDDKVVAIKKSKGGAPTQSDQFVNEVIVLSQINH

RNVVKLLGCCLETEVPLLVYEFITHGTLYEHIHKKRSSLSFELRMKIAVETAEALAYLHS

STSNPIIHRDVKAENILLDDAYTAKVSDFGASRLIPSGQTEIQTLVLGTFGYLDPEYLQS

NQLTEKSDVYSFGVVLVELLTSKMALSKDSCLTSIFLSSMDEDCLNQILDDDIVNEGNIE

TVKNVASLAKRCLRVKGEERPTMKEVAMELEGMRITAKHPWGTNVGFCPEENEYLLGSLD

SDAYVLDVRGGGGSSSGLTTGTGYDSMQIQMLMPYGDGR

>RcWAK9

MAALQGILLIIQLSFVIIRVLIVSTTAAAPQALPNCPDKCGNLTVPYPFGMAEGCYQGND

FFVNCSQATDPPIAYLTKNGTVIITNISLAEGELQISDFIARYCYSTSALRTRRNSPVLR

LPSPYIISDTKNKFIAVGCDTYAIFKGFQGTEQSITGCMSVCNSLKSVDQNSCSGVGCGQ

TNIPSGLHNRTMTLDSYSNHSGIWDFNPCSFAFIVEETQFKFSGNTSFQQLNSTTRLPMV

LNWAIGDEPDPCDVAKKRQDFVCQNYSKCVNPTNRNGYICQCLDGYEGNPYHPNGCHDID

ECKDPNLCNHEECKNLAGSYYCSCSKGYKIDSMNDKSCIKENPKNKSKMTLLLIISLSVS

IGLLVLFVGISWICCGIKRRKYTKLKEKYFKENGGLLLLQQLASHGGTVETTKIFSTEEL

EKATNNYHERRILGEGGYGTVYKGILPDDKVVAIKKSKGGAPTQSDQFVNEVIVLSQINH

RNVVKLLGCCLETEVPLLVYEFITHGTLYEHIHKKRSSLSFELRMKIAVETAEALAYLHS

STSNPIIHRDVKAENILLDDAYTAKISDFGASRLIPSGQTEIQTLVLGTFGYLDPEYLQS

NQLTEKCDVYSFGVS

>RcWAK10

MASLHDRILVTLLSLLMLVAATTTLLAAQTLVPPPQAKPGCREKCGDLTIPYPFGLGEAC

SMRPEFTITCDQSTTATPSANFTNSTFTFPITITNFSLVDGELQVMQDIARSCYDEEGNH

TKRRSSLKLPPSYTISTKNSFFTLGCNVLAVYQQGVDVDYQIGVNVALCMDILGKELTEA

CIGVGCAQTSIPSGLQNISIGVASLNGYNASDPWESKYKCSYAFIVEEGNFTFAPKTSFE

QLNTTNQQLPVAVNWAIGDESCAVAKNLTDYSCKENTICVDRSTITNETAAGYICQCLPG

YEGNPYLGCRDIDECTTSSRDPCPNGKCVNSPPGNYSCKCNKGFRNQDDMTCIPYSWSNN

TSLKISLGVSIGFLVLVVLISWMYWEMNKRRLIKLKKKYFKENGGLTLQQQLASHGGAVE

TTKIFTAEELEKATNNYHTDEILGEGAYGTVYKGILPNKKAVAIKKSKIGAPTQSEQFVN

EVIVLSQINHRNVVKLLGCCFETEVPLLVYEYITHGTLFEHIFNKGKGSPLSWELRLKIA

SETAGALAYLHSSTSTPIIHRDVKATNILLDDNYTAKVSDFGASRFIPLDQTELATLVQG

TLGYLDPEYFHSNQLTEKSDVYSFGVVLAELLTSRVALSFARPDAERCLASFFVSSIEKD

CLIEILDADIVNEGNIDTAEQVAYLAKRCLGIRGEERPSMKEVAMELEGMQNTAKHPWRN

NANLCPEETEYLLGSPINPEAYIVNVKGDGDDGSSGTTSGYDSMQIQMLMPYDDAR

>RcWAK11

MQFILVAVLVLFTFAAAAEEAVAAEAVLPQAKPGCTDRCGNLTIPYPFGIEPDCYMTENF

SVSCNTSTKPPTATWEDSPIVINNIYLAEGEMQIMHYVAYDCYDSQGPTESDKPLLTVPP

FTVSHTRNKFIAVGCDTYALFRGYRADEERNIMTGCMSMCDNLDSVEQSCSGVGCCQTDI

PRGLENCTLTLASYYNHTYILEFNRCSYAFFVEKDEFTFSPNTSFKDLSNTEKLPMILNW

EIGEGPCDEAQKKDDYACKANSKCVNRTISDELSGYICQCQPGYQGNPYLPDGCQDIDEC

KVSNPCTNGRCENSPGSYSCLCDKGYRSNGTNNQSCIKEVPKNQPKTTLLLLLIVSLSVT

GGFLVLFVGISWICCGIKKRKFMKLKEKYFKENGGLLLLQKLANNGGSVETTKIFTTEEL

EKATNNYHESRVLGEGGYGTVYKGVLLDNKVVAIKKSKGGTSIQSEQLVNEVVVLSQINH

RNVVKLLGCCLETAVPLLVYEFVTHGTLYEHIHRKRSLLSFELRMKIAVETAGALAYLHS

SISTPIIHRDVKAANILLDDNYTAKVSDFGASRLVPSGQNEIQTLVLGTFGYLDPEYLQS

NQLTEKSDVYSFGVVLVELLTSKVAFSNDRCLASIFVSSMDEDCLKQILDDAIVNEGNIE

TVENVASLAKRCLRVKGEERPTMKEVAMELEGMTITA

>RcWAK12

MDVSQPTDPPIAYLTKNGTVIITNISLAEGELQITDFIARDCYSASALRTRRNSPVLRLP

SPYTICDTKNNFIAVGCDTHAIFKGFRGTEQSITGCMSVCNSQESVDQNSCSGVGCCQTN

IPSGLHNRTVTLDSYSNHSGIWDFNPCSFAFIVEETLFKFSGNTSFQQLNSTTRLPMVLN

WAIGDEPDPCDVAEKRQDLNIDECKDPNLCNHGECKNFAGSYSCLCSKGYKIDSMNDKSC

IKENPKNKSKMTLLLIISLSVSIGLLVLFVGISWICLGIKRRQYTKLKEKYFKENGGLLL

LQQLASHAGTVDTTKIFSTEELEKATNSYHESRILGEGGYGTVYKGILPDDKVVAIKKSK

GGAPTQSDQFVNEVIVLSQINHRNVVKLLGCCLETEVPLLVYEFITHGTLYEHIHYKRSS

LSFELRMKIAVETAEALAYLHSSTSNPIIHRDVKAENILLDDAYTAKVSDFGASRLVPSG

QTEIQTLVLGTFGYLDPEYLQSNQVTLNICNRTN

>RcWAK13

MASLHERILVTLLSLLMLVAATTTLLAAAQTLVPSPQAKPGCLEKCGGLTIPYPFGLGEA

CSMRPEFTLTCDESTTAPPSANFTNSTFTFPITITNFSLVDGELQVMQEIARNCYDEEGY

NTNSSSSLELPPSYTISTKNNFFTLGCNVLAVYLQGVDVGYQTGVNVALCMDILGEELTE

SCIGVGCAQTSIPSGLQNISIGLASLNGYNANDPWESNYKCSYAFIVEEGNFTFAPKTSF

QQLNTTIQQLPVAVNWAIGDEPCAVAKNLTDYSCKENTICVDRSTISTETAAGYICRCLP

GYEGNPYLGCRDIDECTTSSRDPCTNGKCVNSPPGNYSCKCNKGFRNQDDMTCIPYSWLN

NTSLKISLGKCQYRLLGFSGRNFVDVLGNNKRRLIKLKKKYFKENGGLTLQQQLASHEGA

VETTKIFTAEELKKATNNYHADEILGEGAYGTVYKGILPDKKVVAIKKSKIGAPIQSEQF

VNEVIVLSQINHRNVVKLLGCCFETEVPLLVYEYITHGTLFEHIFNKGKGSPLSWELRLK

IASETAGALAYLHSSTSTPIIHRDVKATNILLDANYTAKVSDFGASRFIPLDQTELATLV

QGTLGYLDPEYFHSNQLTEKSDVYSFGVVLAELLTSRVALSFARPDAERCLASFFVSSIE

KDCLIEILDADIVNEGNIDTAEQVAYLAKRCLRIKGEERPSMKEVAMELEGMQNTAKHPW

GKNANSCPEETEYLLGSPINPEAYIVNVRGDGDEGSSGTTSGYDSMQIQMLMPYDDAR

>RcWAK14

MASLHERILVTLLSLLMLVAATTTLLAAAQTLVPSPQAKPGCREKCGGLTIPYPFGLGEA

CSMRPEFTITCDESTTAPHQQTLQTEIARNCYDEEGYSTNSSSSLELPPSYTISTKNNFF

TLGCNVLAVYLQGVDVGYQTGVNVALCTDILGKELTESCIGVGCAQTSIPSGLQNISIAV

ASLNDGYNASDPWVSNYKCSYAFIVEEGNFTFAPKTSFQQLNTTIQQLPVAVNWAIGDEP

CEVAKNLTDYSCKENTICVDRSTITNETAAGYICRCLPGYEGNPYLGCRDIDECATSSSD

PCTNGKCVNSPGNYSCKCNKGFSNQDHRTCISYSWSNNTSLKISLVALKMKSRPFGSGLH

QEAFFYYFFIFINLKRGKKGNIKDQIFMNFSAHTWQVMSECTKCEPSTLHLRYFIFQLCA

GVSISFLVLVVLISWIYWEMNKRRLIKLKKKYFKENGGLTLQQQLASHGGAVETAKIFTA

EELEKATNNYHADEILGEGAYGTVYKGTLPDKKVVAIKKSKIGAPTQSEQFVNEVIVLSQ

INHRNVVKLLGCCFETEVPLLVYEYITHGTLFEHIFNKGKGSPLSLELRLKIASETAGAL

AYLHSSTSTPIIHRDVKATNILLDDNYTAKVSDFGASRFIPLDQTELATLVQGTLGYLDP

EYFHSNQLTEKSDVYSFGVVLAELLTSRVALSFARPDAERCLASFFVSSVEKDCLIEILD

ADIVNEGNIDTAEQVAYLAKRCLGIRGEERPSMKEVAMELEGMQNTAKHPWRNNANLCPE

ETEYLLGSPINSEAYIVNVKGDGDEGSSGTTSGYDSMQIQMLMSYDDAR

>RcWAK15

MVLYRRMVISQLISLVVVAIAAVAIADQALPPPQALPECLDHCGNLTVPYPFGIGDGCYL

TKKFSLTCNESSQPPTLLWADGGITTTNISVAEGELQILANIARDCYDQTGHQGMYNFPW

LWVSPPFTVSGTRNKFIAVGYDTYAVFRGYRHSPNDKARHVTGCMSVSDILDNTTTAANE

PCKGIGCCQTSIPDGLKNYTIEISSYSNHIFVWNFNPCSYAFIVEEGKFTFNPNTIFQEL

QNNELLPLILNWEIADGSCAEAQKRQDYACKAHSKCVNRTIDIKTKPSGYYCQCLPGYQG

NPYLPDGCQDIDECIASTNPCNNGTCTNSDGDYTCTCNKGFRNENPKSCIQNTPISKNKS

LKVSLGVSLSFLGALLVTFWIYCGLKRRKFKILKEKYFKHNGGYLLQQKLENFDGPQAAK

IFTREELKKATNNFHDSLKIGEGGYGFVYKGTLSNGNEVAIKVSKSSAPMTQSNQFINEV

IVLSQINHRNVVKLLGCCLETQTPLLVYEFVSNGTLYEHIHKKNGKGPLSFGLRMKIATE

TAGSLAYLHYSTSMQIVHRDVKAANILLDENFTAKVSDFGASKLVPEDQNQLSTLVQGTI

GYLDPEYLQSNTLTEKSDVYSFGVVLVELITSQVAISYQKPEAERSLAKFFVTSVEENRL

DQILDHEIIKEGSFETTEQVAHLAKSCLSLNGSDRPTMKEVAMKLEGVLQVMAKHPWGKA

DASPKETDYLQLASHSKAYVVDVRGDEGEIITSIDYDRSIQIQPLMMKPYDGGR

>RcWAK16

MAFLHWRMQMMKLSLVILLAAVTTRAAADQALHGCAEKCGDITIPYPFGIGDGCYLRPEF

NITCNGPTTPTSTNFANSTYTFPMAIANFSISEGELQVMQAVALDCYDNKGVKIANSTST

LELPPSYTISAKNKFFLVGCNNAAIYEGLLQFPDPDEGSDFQAAGFTVTLCVNELGKELH

ESCDGFGCAQTSILSGLQNITVSLDALDADNRTDSWSLKYLCSYAFIVDEGNFTFSPERS

FQQLNTTRQQLPVLINWAIGGEPCEVAKKNHSTYACKENTTCVDRSTITNGTVGYLCHCL

PGYHGNPYLGCQDIDECQASTNPCQNGKCINSPPGNYSCQCNGGYKNKDPITCVEYPKAK

NTSLKISLGLIVGFLVLVVSMSWIYWKMNKRRLIKLKDKYFKENGGLTLQQRRLATHGGG

VETTKIFTAEELKKATNNYNAGEILGEGGYGTVYKGILPDKRVVAIKKSKLGAPTQSDQF

VNEVTILSQINHRNVVKLLGCCFETEVPLLVYEYITGGTLSEHVFKKRQRSVLSWELRLK

IASETAGALAYLHSSTSTPIIHRDVKSTNILLDDNYTAKVSDFGASRFIPIDQTQVATLV

QGTLGYLDPEYCHSNQLTEKSDVYSFGVVLAELLTTKVAVSFARPEEERNLASLFISSME

KECLNQILDAEILNERNMETIRQVANLAKRCLRIKGEERPLMKEVAMELEGMRIEAKHPW

KCGSSCSEGTEYLLGSSNSKTNVADTKGEGVSSGTTSGYASMQIPMLMSHEHGR

>RcWAK17

MALRLSVVAVLLLLSAASTTTTTTAAPAQALTGCSDKCGNLTIPYPFGMKTDCLLRKEFF

INCSHTTQPPTAYLMYSDLIVTNIYLDEGELQILNDVGRACYDEEGGYQTDRSIKPQLLL

QPPYTISNTKNKFYAVGCDTSAIVNAFRGKEEFITGCMSICNSLGSVEKSCSGVGCCQIN

IPSGLYNLTVELNSFKNHTRIWNFNPCSYGFIAEEGLFNFSPSSFEDLNYSALLPMVANW

AIGNEIDTCETAQKRLDYACNANSNCVNRPIDGSVGYLCQCLPGYEGNPYHPDGCQDINE

CMALNPCQNGECLNSPSGYSCLCHKGYKHNGISCIKDDSERRFKIVLLRFVSLVGGSTLG

LIFLSIAAWWLVQVIKKRKNKKRKEKFFKQNGGLVLEQQSSSGDQANVQKIKLFNSRELE

KATDKFSIDRILGQGGQGTVYKGMLMDGQIVAVKKSKIVNGGEVQQFINEIVILSQINHR

NVVKLLGCCLETEIPLLVYEFLPNGTLSQYINGQDEEFPLTWGTRLRVSTEVARALSYLH

SAASIPIYHRDIKSTNILLDAKYRAKIADFGTSRSISIDQTHLTTLVHGTFGYLDPEYFQ

SSQFTEKSDVYSFGVVLVELLTGQKPVSMMRSQESRSLATHFLLSMEQNRLFDILDAQVM

KDGSKEEIIAVANLAQRCLNLNGRKRPTMKEVAVELEGVQLAVKDAVHVQQNLVEEGDVG

TDEITTEAYCDVVSTSIGPFTDGGTTGSLSDEQPLLFYNTQ

>RcWAK18

MALQVSLVAVLLLLLALSAASTTTTRKEPTQALPGCPNKCGNLTIPYPFGMKKDCYLREE

FFINCSHATQPPVAYLGRGNLKVTDISVDDGELRILGYVAKKCYNALQGRYFTSLDGELH

RIWLGLPYTISHSKNKFYAVGCDASAVVKGFRGEEELITGCMSICNSFGSVDNHFCSGVG

CCETYIPSGLKNMTLKLNSYQNNLRIIDFNPCSYAFIVEEGQFNFSRASFKDLNNTIELP

MVLNWAVGNDTDPCDEARKRKDFACKANSLCVNWEMAINESVGYLCKCSPGYEGNPYHPD

GCQDIDECKASISPCNHGTCINSLGNYSCLCPKAYKNRTNAKGCLKLEINQPARKLFLIT

LGTGSSFGLIFLAIAAWWLVQVIKKRENKIRKEKFFKQNGGLVLEQQLSSGDQANVEKIK

LFNSRELEKATDHFSIDRILGQGGQGTVYKGMLMGGQIVAIKKSKMVNGGDREVRQFINE

IVILSQINHRNVVKLLGCCLETEIPLLVYEFLPNGTLSQYIHDQDDEFPLTWGTRAIATS

IPIYHRDIKSTNILLDAKYRAKIADFGTSRSISIDQTHLTTVVHGTFGYLDPEYFQSSQF

TEKSDVYSFGVVLAELLTGQKPVSMTRSKESRSLATHFLLSMEQNRLFDILDPQVMKDGI

KEEIIAVAYLAQKCIDLNGRKRPTMKEVAAALEGVRLAVKDADHVQQNLVEKRDVGTHEI

ITQAYCDIVSTSAGPFTDSGTAGSSSI

>RcWAK19

MALRLSLVAVLLLLSAASTTTTTAAPALAKSGCPDKCGNLTIPYPFGMEEGCYLRKEFSI

NCSNTTQPPTAYFAKDSNIVVTNISLDEGEVRILNFIASDCYEAPGNTTSGFFSEITLNL

PYTISDTKNRFIVVGCDTYAFLRGYRVEEGDQFSTGCMSICNSLDSVDKNSCSGVGCCQT

NTPSGLYNTRVTVNSFYNYSDVWKFNPCSYAFIAEQGQFNFSPASFEQLNGTDQVPIVLN

WAIGNDNDPCDEAQKREDFACKAHSKCVNRPINGSIGYLCQCLPGYEGNPYHPDGCQDID

ECEVPNLCQHGKCLNSQGNYSCSCHKGYKHDGMNDKICIKKVSKMEVLLIVSLGVSVGFL

VSFIGIAWLWWANKKRQFIKLKKKYFKENGGLLLQQQLASHGGTIDTTKIFSAEELQKAT

NNYHESRVLGKGGYGIVYKGILPDNQVIAIKKSKVCAGSQSEQFVNEVIILSQINHRNVV

KLLGCCLETEVPILVYEFISHGTLHEHIHKKRSSLAFELRMKIAAEAAGALAHLHSSIST

PIIHRDVKAANILLDNKYTAKVSDFGASRLVSSGEAGIQTVVLGTIGYLDPEYLHSNQLT

EKSDVYSFGVLLAELLTSKVAVSKDKFLASIFVASMETDCLNQVLDDEIVNEGNIEMVKY

VAKLARRCLRVKGEERPTMREVAMELGGMIMTKHPWGSADHYFPEETEHLLGSPNTSKDY

FVNIDGGGGPGTATGSSMQIDMSMSSYADGR

>RcWAK20

MAVHGRMLVMQLIIAVAIAAAAADRTPPRQARPNCPDHCGNLTIPYPFGIGKGCFLRNGF

NLTCNETAKPPTTLWGDGSFTVTNILLAEGELEVMNNISHDCYDKQGNQTKDMYNFPSLR

LQPPFTISGTRNKFIAVGCDNWAIFKGYRHYPHDEDRYITGCITVCNNLDSVVNGSCSGI

GCCQTAIPSGLKNGTVKLDSYSQHTAVWDNNPCSYAFIVEEGRFTFNPKTSFEELNNTKK

LPVIINWAIHEGSCALAQNSSGYACKANSSCVNRTVDYQTEPTGYYCQCLPGFEGNPYLP

DGCQDIDECMAPSSPCNNGICQNSPGGYSCLCNKGFKNQDSKICIKNTPVSKNIPLKISM

GLSIGFFILVVLIWWTYCGMNKRRLVRLKEKYFEENGGLTLQQHLASHGGSIETVEIFTA

EELQKATNNYHADEILGEGGYATVYKGIVHDKNVVAIKKPKIGTPTQSDQFVNEIIVLSQ

INHRNVVKLLGCCFETEVPLLVYEYITHGTLFEHVFNKGKGSSLSWELRLKIASETAGAL

AYLHSATSTPIIHRDVKTTNILLDDNYTAKVSDFGASRFIPLDQTQLSTLVQGTLGYLDP

EYFHSNQLTEKSDVYSFGVVLAELLTSRVALSFARPDKERCLASFFVSSIENECLIQILD

ADIVNEENIETVEQVAILAKRCLMIKSEERPTMKDVAMELEGMRIMAKHPWKNGNSGFEE

TEYLLGSPNSAAYVMDIGGGGAGGSSVTNTGYDSMQIQMLMPYDDGR

>RcWAK21

MALVDVGMFVMQVSLAIVAASITTTSTQVAADQASLLQPQAKPNCPDRCGDLTIPYPFGI

GDGCYRQLPEQNHQFSITCDQSTKPPSAYWTSAKRWTSAKVRVTNFNISEGELQIMSYIA

KDCYDAQGSRTYRTTPLLRVDPPFTISSRKNKFFAVGCDTYAIFRGFRGEEELVTGCMSV

CTTLGGVDWESCTGVGCCQTQVPHGLKNRTVRLSSYNGHKEIWSFNPCSYAFIVQDGLFT

FNTTSFELLNNIQELPMVLNWDMGNVPCEVAGKRSMDYACKANSKCANRRNFTGGNGYYC

QCLPGYEGNPYLGCQDIDECSAETNPCENGTCLNSPAGNYSCKCHKGFRSDGPWKCVPSP

KVNNTSLKILLGISISVFVLMTGIFGIYCGFKRRQFERLKAKYFDANGGPKLLEKLSTQK

ESLETKAQIFTAEVLKKATNSYHESEKLGEGGYGTVYKGILKDERVVAIKKSKNTAQIES

DQFVNEVVVLSQINHKNVVRLIGCCLETQMPLLVYEFVGNGTLYEHIHRINGREPLSWAF

RLKIAAETASALSYLHHSTSIPIIHRDVKATNILLDEKFTAKVSDFGASRLVPEDPNQLP

TIVQGTMGYLDPEYLQSNILTDKSDVYSFGVVLVELLTSKKVVSFDRSEEERNLANVFVS

AAKKGMLNPILDDEIVKDGNLEIIEKVANLAKNCLSLKGDDRPSMKDVERELDGILLQTL

AIEPGVKSHSSAKEIDHSLESSSSAFVVKVGGEDEGDNTTNVTSYDVSMENQAQMLKPFD

DGR

>RcWAK22

MSMRIKKMFLLQLVLAAIEVLLVSSQTVDITREGCQDKCGNVSIPYPFGTRGGCYHDEDF

LITCNSTSYNPPKPFLRKGNIEVTNISIDGKLNIMQYIARDCYDSSGFRIKKNSASTRLS

KFIISNTDNKFIAVGCDTYAGIQGFQGKIMKYTGGCISTCDSIDYVANDSCSVIGCCQTS

IAKGVSHFDVFLHSYQNHTDVWSFNPCSFAFIVEESKFNFSRAMLMDLKDVTVLPAVLDW

SIGNENCKQVEGKMIAGSACQGNSTCVDVENGSGYRCKCKDGYQGNPYLSNGCQDIDECE

DPKLSNCAQKCVNTEGNHYCSCRKGYHGNGIAGEEGCIANRTLVVQITVGIAVGLISLLT

CCSWLYLGYRRWKLMKLKERFFRQNGGLMLQQQLSERQGGATNQTAKIFTAEELEKATNN

YHETRIVGKGGYGTVYKGILSDGRVVAIKKSKLVDQSQIEQFINEVLVLSQINHRNVVKL

LGCCFETEVPRLVYEFVTNGTLFDYIHNKSKASNFAWETRLRIAAEAAGVLSYLHSAASV

PIIHRDVKSTNILLDETLTAKVSDFGASRLVPLDQTELSTMVQGTLGYLDPEYFQTSQLT

DKSDVYSFGVVLVELLTGKKALSFDRQEEDRNLAMYFLSALKENRLVQVVDECVLNGAND

KQLKEVSNLAKRCLKVKGEERPTMMEVASELEGLRRMAMHHPWVNNDESNAQENESLLGE

ISLETFSHGGGGDTSSGYDTMRNHIILPVNDGR

>RcWAK23

MLVIQVISLLVGATAVAADQAQPGCLDRCGSVKIPYPFGIGEGCSLREGFNLSCDESAEI

PKFAAGVPVTNISLAEGELHLTTLKAYDCYDKNGIMTEHHSPQFAFPPSFTISSTRNNFT

AAGCDTLAFLQGNGPDGDTYQTGCTSICNNLKSAVNGSCSSIGCCQTSIPIGLKNRTFTL

TSRSNHTKVWGFNRCSYAFVVQVDKFTFNPNTSFLELYNTERLPTILNWEIQDGPCAEAQ

KRPDYACKENSKCVNRTINDQTEPSGYYCQCLPGFEGNPYLPNGCQGALFPSLLYLCQFL

QLEIKAQNPQISSPDSLSDIDECKASQIDPCNNGRCTNSHGNYSCLCNKGFRNQDARTCI

KDNIDNQSARKLFFILMGTGSGVGLLLLVIVVWWLRNVIKKRKDRIRKEKFYKQNGGLLL

EQQLSSGEVNVEKIKLFSSKELEKATDRFNVDRILGEGGQGTVYKGMLTDGRIVAVKKSK

LANGGEVEQFINEIVILSQINHRNVVKLLGCCLETEVPLLIYEFLPNGTISQYIHNHQDE

EFPFTWETRLRVSTEVAGALFYLHSAASYPIYHRDIKSTNILLDDKYRAKVADFGTSRSV

SVDQTHLTTLVHGTFGYLDPEYFQSSQFTDKSDVYSFGVVLVELLTGQKPVSVTRPQESR

SLATYFLISMEQNCLFDIIDAQVMKDGGKEEITKVANIARRCLNLNGKKRPTMKEVAVEL

EEVRSTIKSSDHVGQIKLAEEGDFRAHEITTKAYWDVVSTSTGPFTDGSTGSSCDVQPLL

FSN

>RcWAKL1

MPVLLVLLLLGFCTEACHAKEKSLIKSFPVKHRRLTSFTMNIAASIPSFPARFILILLVV

LGLCTVASRAKDDHHCEPSSCGHIRNISYPFRLNDDPKKCGKSRYNLSCENNLAVLHLYS

GRYYVLAINYGNYTIRVVDANIRKDNCSSLPRYSLASYNFSYKDPYSITYSSWVDRFELS

KPIIFFTCETRPVNSYLYMDTAPCINAMSGHSFVITESLNASNLWDSCRIELMVLTSSSI

GITDVNRSYIDIHNKLLYGFELSWLESYRSSGIKYWSGESCYVDTTNKVNCVVLWTAPKA

VFGVPCVVVLLILKLKRRHLSMYNVIEEFLQSHNNLMPIRYSYSNIKKMTKGFKEKLGEG

GYGSVYKGKLRSGHLVAVKMLGNSKTNGQDFMNEVATIGRIHHVNVVRLIGYCADGSKRA

LVYEYMSKGSLDKHIFPKEGFISLSCKEAFEISLGVARGIDYLHEGCDMKILHFDIKPHN

ILLDEKFVPKISDFGLARLCPSDESSLILTAVRGTFGYIAPEFFYKNIGGVSNKADIYSF

GMLLMEIAGKRRNLNALAPNTSQIYFPSWVYDQFNEEKEVEIEDATDDEKKLTKKMLMVA

LWCIQMKPSDRPNSMNKVVEMLEGEVEFIQMPPKPFLYPQDMPVG

>RcWAKL2

MYLPMINSISLQGRLPMPALLVLLLLVLCTAACHAKDDPHHCEPSSCGNIHNISYPFQLN

DDPNKCGKSMYNLSCEKNVTVLHLYSGKYYVLAINYGNYTIRVVDANIRKDNCSSIPRNS

LASYNFSEEDPYSIDMLKGQGHIYWGDAHRFELSKPIIFMTCDTRPVSSYLYMDTAPCIN

AQSGHPFVLTGGLNASDLWDSCRIELMVLTSSSIGTKDINSSYIDIHNELLYGFELSWYY

SYRGSRTYWECYVDTDTNTVDCVLGATAIKAIFGIPCVVVLLILKLKRRHLSMFNVIEEF

LQSHNNLMPIRYSYSNIKKMTKGFKDKLGEGGYGSVYKGKLRSGHLVAVKMLGNSKANGQ

DFMNEVATIGRIHHVNVVRLIGYCAEGSKRALIYEYMSKGSLDKHIFPKEGLISLSCKEA

FEISLGVARGIDYLHQGCDMKILHFDIKPHNILLDEKFVPKISDFGLARLCRSDESSLIL

TAVRGTFGYIAPEFFYKNIGGVSNKADIYSFGMLLMEIAGKRRNLNALAANTSQIYFPSW

VYDQFNEEKEVEIEDATDEEKKITKKMLMVALWCIQMKPSDRPNSMNKVVEMLEGEVEFI

QMAPKPFLYPHDMPVG

>RcWAKL3

MLVCVLHDRRPTQDTREACHAKDDHHCEPSSCGDIHNISFPFRLNDDPKKCGDQRYSLSC

ENNVTVLHWYSGKYYVLAINYGNYSIRVVDANVRKGNCSSIPRNSLASYNFSYYDDPYST

SQYRKYQNRTREVIQLSKPIIFMTCETRPVNSSLYVETAPCVNATSGHSFVITQTLNASD

LWDSCRIQLMVLISSSIGTKDKNSSYIDIHNELLYGFELSWVQSYGRSGDYCSIEGDTNE

VYCSGLFYTILEGDKYIVVFYDGPLFNTILGVSARCILAAVVLWTPTKVIFGIPCVVVLL

ILKLKRRHLSMYNVIEEFLQSHNNLMPIRYSYSNIKKMTKGFKDKLGEGGYGSVYKGKLQ

SGHLVAVKMLGNSKANGQDFMNEVATIGRIHHVNVVRLIGYCAEGSKRALIYEYMSKGSL

DKHIFPKEGLISLSCKEAFEISLGVARGIDYLHQGCDMKILHFDIKPHNILLDEKFVPKI

SDFGLARLCPLDDSSLTLTAARGTIGYIAPELFYKNIGALWCIQMKPSDRPNSMNKVVKM

LEGEVEYIQMPPKPFLYPQDMPVVDDEDDSERTLSSSLPLKNEAQESISLIQNPN

>RcWAKL4

MCYMTFVRESLRPNHCSLVLNFKNYIFAESTARSCGSCGTNLIPYPLSTGPKCGDVTYYS

FHCNISTGQLSFEAPSGTYHVTSINADTQTFVIQANDADECRDKKFLKLIQSSPYNVTNM

CNADPTRFSPDLSFKGGYEVEVAWKSPLEPPCSSSTDCKDWPRSICDAALDGKNRCLCPA

NSKWDSRSLNCTQEVGHRKQTGEQGKMTLALIIAKAGQIFKSVQHDSERKVKNLIESGRF

KDDDTEGIDVPSFDLESILVATTYFSNANKLGQGGFGPVYKGKLPGGEEISVKRLSSCSG

QGLEEFKNEVLLIAILQHRNLVRLLGYCAEGDEKMLIYEANKSLDSFIFDSRLRVIHRVL

KTSNILQGEEMNPKISDFGLARIFGGNETSENTNRVVGTYGYMSPEYALDGLFLAWHLWK

EQKALYLLEQTLGHSCNKDEYFKCVNVGLLCVQEDPGDWPTMSQVVFMLGSETATIPTPK

QPAFIVN

>RcWAKL5

MGKINLVAWVSIAALFLVIALLSGHSCNAKDEGSKSTTSSSGNIHTRSFLGVFVPKARGR

DNQKCVPSSCGHIDNISYPFRLEHDPKHCGDSSYTLECNNNVTILHLYSGEYYVKEINYR

NSTIRVVDPGLEKNNCASLPRFPLAQSNFSSLSFTYGPVSTPLTFLKCQNPVHSSLYVDT

ASCIKSEAYGYVKVGITTTSDLEDGCSIDSTTMITTFARTKRNVSYKEIHKELVYGFQLT

YEMTYIFVDCRRQGQWSSAPGRCFPHSITGFFQLLWEFIYYYIVLPLYHFDRFLLEMISI

VPVVFTVKFILGAPFVIALLIYKWRRRHSSTNNSIEDFLHSDNFMPIRYTYSNIKKMSNG

FKDKLGEGGYGSVFKAKLRSGRLGAIKLLGKPNANGEDFMSEVATIGRIHHINVVQLVGY

CVEGSKRALVYDFMSNGSLDKCIYSKEGFNTLSCKKMYEIAVGVAHGIEYLHRGCEVQIL

HFDIKPHNILLDENFIPKISDFGLAKLYPRDNSMVSSMAARGTMGYIAPELFYKNIGGVS

YKADVYSFGMLLMEMASKRKNVNALVENSSQIYFPSWVYDQYNEGKDFEIGDATTEEKQI

IKKMIITALWCIQMKPSDRPSMKRAIAMLEGDVECLQMPLKPSLCPEPMTVGNLIPTSSD

VELTCSLSAR

>RcWAKL6

MGISGISWSLALVVVSLFFQTCNANTKDDSPKCTSSCGNIHNISYPFRLKHDPKHCSSRF

TLLCENNITILDQSAVGRFYVQAINYENQTIRVVDPDLFQNNNCSSIPHNRLPFLSGRAM

YSYALFSTPIFFLKCNNPVKSFMYVDTAPCINTSAGTASSLASPESPPNTYGYVKVGDME

VGDLNEGCSTEWMASAMLNSFNLYNSSYKYIHNALVYGFELHYTISEPCEQWSTNAKCFP

RSTKGYKWLPSNSTGLLFLVKFILGTPFVTVILIKKWRTRHLSSYRTIEDFLHNGSNFVP

IRYSFSEVKKMSNNFKNKLGEGGYGSVFKGVLRSGRFGAIKMMANSKSSGQDFVSEVATI

GRIHHVNVVQLIGYCVEGSKRALVYDFMPNGSLDKYIFSNAETIPLSVKKMYEISLGVAQ

GIEYLHQGCEMQILHFDIKPHNILLDENFIPKVSDFGLAKLYSTDDSIVSLTAARGTMGY

IAPELFYKNIGGVSYKADVYSFGMLLMEMASKRKNFKELAGHESQTYFPSWVYDQCEKGR

DLEMGDATDEEKKIIKRMVVTALWCIQMNPSHRPSMRKVREMLEGDVELLQMPPNPFTCS

EEIQIPLADVQNNIDPTCSNAELTCTLSAR

>RcWAKL7

MGLSGIRSLSLCGWVSIVAFLVLGLSGHNCKSTASSSGKIHRTSVLGSWESNPRDRYTCV

PSSCGDIHNVSYPFRLQHDPKHCGHSSYTLECDNNVTMLRLYSGKYYVKEINYRRRRIRV

VDPGLEKNNCASLPLFPLADYQFHYEDPYETFLNNQVTWLKCANPVNSSRYINTAPCIKS

EGYGYAIRHMSLQDLEIGCSIVWMGFYAAMDEDIGHADSYQDIHNNLLYGFNLEYTNAYD

IPADCFGNGWHSHGRCYPRSIRGLFQLGWTVFRGYNDYIFWGTIATASVVGVKLIFGAPF

VIALLIYKWRRRYWSTYNTIEDFLHSDNFMPIRYSYSNIKKMSDNFKDKLGKGGYGSVFK

CKLRSGHFGAIKLLGESNANGEDFMSEIATIGRIHHVNVVQLVGYCVEGSKRALVYDFMP

NGSLDKYIYSKEGSITLNYKKLYEIAVGVAQGIEYLHQGCEMQILHFDIKPHNILLDENF

TPKISDFGLAKLYPANNSIVSSMAARGTMGYIAPELFYKNIGGVSYKADVYSFGMLLMEM

ASRRKNLNAFVDHSSQLYFPSWVHDQYNDGKDLEIGDATAEEKKLIKKMIVTALWCIQMK

PSDRPSMKRVTQILVGDAEYLEMPPKPSLCPQPMPVRNLIPGCSNVELTCTLSAR

>RcWAKL8

MGIGGIRSLSLCGWVSIVAFFVLGLSTASSSGKIHTTSVLGDKGTCVPSSCGDFQNISFP

FRLKHDPKHCGGQEFFTLECHNNITVLRLFSHEYYVKAIDYDNRTIRVVDPGLEKNNCTS

LPRFPLAPSNFSYDSIGETVWPYSYLVTFLKCANPVNSSLYVNTAPCIKSKGYGYAILDM

SPQDVEIGCRIDWMGMITSMHGKDRNISYQDIHNELVYGFVLQWYDFSVCPGNHWHYIDR

LHTYAPCYRRSISGFFNLVLTVLIGKGHFFKSYFGKLTKLFYPLICEPNFTSKRWLCLVL

HVALHFQNLIYSRAPNEENIVKRENIYVAFVVQLNLTLVTNFADRGHLYLDLIVAASVVG

VKLIFGGPFVIALLIYKWRRRHWSTYNTIEDFLHSDNFMPIRYSYSNIKKMSDGFKDKLG

EGGYGSVFKGKLRSGHFGAIKLLGKSNANGEDFMNEVATIGRIHHVNVVQLVGYCVEGSK

RALVYDFMSNGSLDKYIYSKEGSITLSYRKMYEIAVGVAQGIEYLHQGCEMQILHFDIKP

HNILLDENFIPKISDFGLAKLYPVNNSIVSSMAARGTLGYIAPELFYKNIGGVSYKADVY

SFGMLLMEMASRRKNLNADVDHSSQIHFPSWVHDQYTDGKDLEIGDATTEEKKLIKKMIV

TALWCIQMKPSDRPSMKRVTQMLVGDTEYLEMPPKPSLCPQPMPASNLIPICSNVELTCT

LSAR

>RcWAKL9

MGISGIRSLSLCGWVSIVALFVLGLSTASSSGKIHTTSVLGDNRTCVPSSCGDVHNIRYP

FRQQNDPQHCGDQRFTLECHNNITVLRQISHEYYVKVIDYDNSLIRVVDPGLEKNNCSSL

PRFPLAPSDFSDGYTYSLNDSIPLTFLKCANPVNSSLYVNTAPCIKSKGHGYAILGHTSL

EDLENGCSIDWIWKGMNRMITSTDGKDRNISYQDIHNELMKGFELHYSLPNYYLPIHYPQ

RNYCHGQWRYRNKTCYPRSIPGLFQLVRTALTDHGAFVRIIAVGVLDCVVGVKLIYGGPF

VIGLLIYKWRRRHWSAYNTIEDFLHGDNFMPIRYSYSDIKKMFGNFKDKLGEGGYGSVFK

GKLRSGHFAAIKLLGKSNANGEDFMSEVATIGRIHHVNVVQLVGYCVEGSKRALVYDFMS

NGSLDKYIYSKEGSITLTYKKMYEIAVGVAQGIEYLHQGCEMQILHFDIKPHNILLDENF

IPKISDFGLAKLYPANNSIISSVAARGTMGYIAPELFYKNIGGVSYKADVYSFGMLLMEM

ASRRKNLNALVDHSSQLYFPSWVHDQYSDGKDLEIGDATAEEKKLIKKMIVTALWCIQMK

PSDRPSMKRVTQILVGDVEYLEMPPKPSLCPQPMPVSNLIPACSNVELTCTLSAR

>RcWAKL10

MGKRSFSLLCARVSIVSFIVLGLSGNTTTCNAASSSSNIHKSTIPGDNDKCVPSSCGDLQ

NISYPFRLEHDPSYCGESSFTLNCDNNVTMLHLYSGKYYVKAIDYDNTTIRLVDPGLEKN

NCTSLPRFPLGKYNFSLMDTYQPLNISAPPAVTFLNCAKPMNSSLYVDAAQCVKSEAYSY

VMLGGTKVADLEPGCSIVWMTMISVSNPFWGKGKDQNVQDIHNQLVYGFELQYHDIVSSR

AQWCLDEHTCFTRSIEEDDQFCIAIIANVVMVKLIFGGPFVIALLIHKWRRRHRSTYNTI

EDFLHRDNFMPIRYSYSNIKKMSNGFKDKLGEGGYGSVFKGKLQSGHFGAIKLLGKSNAN

GEDFMSEITTIGRIHHVNVVQLVGYCVEGSKRALVYDFMPNGSLDKYIYSKQGSITLSYK

KMYEIAVGVAEGIEYLHQVLECY

>RcWAKL11

MLPLSSLASFLLLTFFKFLAISHSLQEQHQLPRPNLCHEKCGDLQISFPFHLNKSCSSLS

DAFHLSCVNSTNIFLNIGSESYRVLEFFSDGLLVDFPGSSSYCRQYNDLNSFDFLGNDHF

GLSADNVIGLYDCEDSSLCKTECETIDLPGCDGNESQGSPACCYPLSDYSLWHLGDKFSV

FSKFGCRGFSSWVVQRGSNLGKRGVKLEWAVPRNSSKGVCATNGYIINATSIQAGVRCAC

QDGFIGDGFATGEGCIMSCIKERREAYGADCFKKRHGSKKLLIIVGVLAPLFIIASLIAL

LYLLKRPVKPGTFDPAQKVHFHSTISFRKASRTRLFTYHELEEATKAFEEDQKLVSGNNG

TIFSGVLGDGSHIAVHKIDCENEKDLIQVLSQIEALSAILHRSITRFLGCCIDLAYTPLL

VHEYPANGTLEDHLHQTGGQHVALDWYKRLNIAAETASVLAFLQYEISPPIFHCDLKSGY

IFIDNDFCSKLCGFGLLVSRHEEGSRFQRTDVYALGVVLLEMIAGSNCLDLQISLQKIRG

GKLEEIVDPLLYYHEQPSYHREQIETVADLAMRCLLFGGDGKLGMYDVAKELVHIRRESS

DGGSKRGPALEETFSNSSLLQMISMSPDSAYRWECLRIFDQLPLFSQPLSFFTYYKYFDP

NSASLGTYSSSLSSFCLYSQIALAIKMTDEKKPTPKLNERILSSLSRKSVAAHPWHDLEI

GPSAPQIFNVVVEISQGSKVKYELDKKTGLIKVDRILYSSVVYPHNYGFIPRTLCEDNDP

LDVLVLMQEPVIPGAFLRAKAIGLMPMIDQGEKDDKIIAVCADDPAYNHYSDIGELPPHR

LTEIRRFFEDYKKNEHKEVAVDEFLPCSTAAEAIQYSMDLYAEYIMLTLRR

>RcWAKL12

MAVTPNLRLLTASLLLLSCAWTSLSVQLCPPCGNTTVPYPLSTSPTCGDQSYKIRCDAAA

GSLLFDTLNNSYPIASISPSAQRLVVRPSSFVSSNSCVTSDIVHQGIQLNDSLPFNVTSS

NTILYLNCTDTLLRSPLNCTSSSLCHTYVNNTNAVGSCQRAPICCTFRAGGSSTSYMIRV

RDSGCSAYTSFVNLDPVLPVNRWPEPGVEIQWVAPREPVCTAQSDCDGKSVCGSDPVQNG

VRRCFCNSGFTWDPVSGFCVDDSVGSKDRTGLIAGLTSGIGASLLAAIIAIVLYKRHRRI

KEAQDRLTKEREAILNANGGRAAKVFTGKEIKKATNSFSRDLLLGAGGYGEVYKGTLEDG

TVVAVKIAKIGNTKGTDQVLNEVRILCQVNHRSLVHLLGCCVELEQPIMVYEYIENGTLL

EHFQARKGVGRTPLSWTQRLQIAHDTAEGLAYLHFSAVPPIYHRDVKSSNILLDNKLNAK

VADFGLSRLAHTDMSHISTCAQGTLGYLDPEYYRNYQLTDKSDVYSFGVVLLELMSSQKA

IDFNRDPDDVNLAVYVQRLMAEEKLMDAIDPMLKEGATALELDTMKALGFLALGCLEERR

QNRPSMKEVVEEIEYITSIATAKVNDD

>RcWAKL13

MATSQLAALYITVFLLSMSTSTPTLAVSVPMTKPGCSTHCGTVEIPYPFGIEPDCYINEW

FQILCESSTGLLKPLLNSMHLEVWDISVAGTIRVTNPITFSNCSNSLVNPVQTFTLEGSP

FLFSQKNRFTSISCNEIALLTSSDGYPIGGCLSICDNGSSLTPSSTGRILNHDSCSGTNC

CQSTIPLDLYAFNTSFQKLNDVKIGSACNHAFPVDQDWFMSNSINISPAGEIFDSIPVVL

DWNLYNYSPPALSEDDHFVKSFHCERIQNKMQPQNISTRCFCSKGFQGNPYFFLGCQQDE

CGARSHICQRSFPSSVSYTGLGLLIFLIVVARWLNRVIKKRKHNKSKAMFFKQNGGLLLQ

QQSATGEVHVEKIKLFTSKELLKATNEFNVNRVLGHGSQGTVYKGMLEDGKIVAVKKSKI

VDGGEVGEFINEIVILSQINHRNVVKLLGCCLETEVPLLVYEFILNGSLSQYIHHQNDFR

LTWEMRLRVSMQVAGALSYLHSSTCLPIYHRDIKSGNILLDDKFNAKIADFGTSRSISID

KTHLTTLVKGTFGYLDPGYFQSSQFTDKSDVYSFGVVLVELLTGQKPVFLTPSGEWSSLA

THFIESMEENSLFDIIDARVRVMKDGGKEEIIAVANLAKRCLDLNGKKRPTMKEVAATLE

EILLLVTS

>RcWAKL14

MPNHGQTPNLYPTPFWLFSDRNMFTAVSCGRLASLTYFGGTKMAVGLSICQGYSSILVNN

NCRGINCCQTPLPSDNGGNFTTSFGPLIINGTSERTCKYAFLVDPEWFASNSTNTPAIAQ

LDDVPNPHSARLDYWRITGGIDNCEFESGPCSCLEGYQGNPYLRGGCQDINECEDPNRPN

ICGSGKICYNFDGTFECYTPLSPQPRPLQPIKLSIIVLCSVFGLLLHIIGAWWLHKVIRK

RKNIKRKQKFFRLNGGLLLEQQLSSGEVNVEKIKLFNAKELEKATDRFNVDRILGEGGQG

TVCKGMLTDGRIVAVKKSKIVDGGEVNQFINEIVILSQTNHRNVVKLLGCCLDSEVPLLF

YEFLPNGTLSQYIHSRES

>RcWAKL15

MEVQLLLLDISLMTLWSLSCSAILSVSSSPLLAKPNCQSHCGDVEIPYPFGIGPNCSHGP

WFQILCDNSTNLPKPFLNLTDGRPEVLEISLAGTLKIKNPITFSDCPNKPNHRQTPNLFP

TPFWFSDRNKFTSVSCGRLAWLVSQSTAGTMTSKSALGTEPAVCLSVCDYPSSILVNNSC

GGIDCCQTSLRSSIGTTFSISFGPLILNDTSERTCKYAFLVDPEWFTSNSTNTSAIAEMD

DVPMVLDWTTAGSIDNSTEENRNCTSSVGPCSCLEGYQGNPYLRGGCQDINECEDPQRCG

SDICSNFNGSFYCYKPLSPQPRPLQPIKLGIIVLCSVFGLLFHIIGAWWLHKVIKKRKNI

KRKQKFFSLNGGLLLEQQLSSGEVNVEKIKLFNAKELEKATDRFNVNRVLGQGGQGTVYK

GMLTDGKIVAVKKSKVVDGGEVNQFINEIVILSQINHRNVVKLLGCCLESEVPLLVYEFL

PNGTLSQYIHSRDEDFPLTWEMRLRVGTEVAGALSYLHSSASMPIYHRDIKSSNILLDDK

YRAKVADFGTSRTVSIDKTHLTMTHVNGTFGYLDPEYFQSSQFTDKSDVYSFGVVLAELL

TGQKPVSVTRSQEGKQDGRSLATHFIVSMEESCLFDILDAEVMKGGKKEEIVAVANLAKR

CLNMKGKKRPTMKEIAAELEGILMSSKSSDVEHIAEVERVQTEIAEVWDVSTSTGSHMDT

GSSLYVQPLLSFRTR

>RcWAKL16

MEVQLLLLEITLMTLLSLSCSAILGVASSPSLAKRDCQSRCGGIEIPYPFGIGPNCHLLK

NDSFYDKYTRLFQILCDNSTNPPKPFLNLTDGRREILEISLAGTLKIKNPITFSDCPSKP

NRSRIPNLSPTPFWFSDRNMFTAVSCGRLASLTYLGGGATEMAVCLSICDYSSILVNNSC

RGINCCQTPLPFNIGANFTTSFGPLIINGTNGTSGRTILNGTSGRTCKYAFLVDPEWFTS

NSTNIYDIAELDDVPVVLNWRILGGIDNSTKDNVNRNCESAAGPCSCLEGYQGNPYLRGG

CQDINECEDPNRPNICGSDKICSNFNGTFVCYTPLSPQPRPLQPIKLGIIVLCSVFGLLF

HIIGAWWLHKVIKKRKNIKRKQKFFRQNGGLLLEQQLSSGEVNVEKIKLFNAKELEKATD

RFNVHRILGQGGQGTVYKGMLTDGRNVAVKKSKAVDGGEVSQFINEIVILSQINHRNVVK

LLGCCLESEVPLLVYEFLPNGTLSQYIHSRDEDFPLTWEMRLRVGTEVAGALSYLHSSAS

MPIYHRDIKSSNILLDDKYRAKVADFGTSRTVSIDKTHLTMTHVNGTFGYLDPEYFQSSQ

FTDKSDVYSFGVVLAELLTGQKPVSVTRSQEGKQDGRSLATHFIVSMEESCLFDILDAEV

MKGGKKEEIVAVANLAKRCLNMKGKKRPAMKEIAVKLEEILMSSKSSDVKHIAKVEHVQT

EIAEVWDVSTSTGSHMDTGSSLDGQPLLSFKSR

>RcWAKL17

MVAQLLFHITLLLWSMSSTEVLVVASEAPIVATEAPIAKPNCPSHCGGIEIPYPFGIGAG

CYIHDDWFQVFCDNSTGSPTPFLNGTNLEVLDISVEGTLKVRNPITFSNCTDKPNRQAVN

LEGSPFMFSQKNMFTAVGCGVMATITSNSNGVTISAGCRSECYDYTVSKNSSSHNTCNGV

DCCQTVIPPSLSAFNTSFQQANDNYTNSSCNYAFLVDRDWFESFAKNSTNISATSDMDQV

PVELKWNLYHSTTDVFGTFKETNVSVRYADDLDGYCETYNDSSSSYESLRLECSCGGGST

GNPYLPQGCIDINECEDPENRASCSAGLVCVNYNNGWGCTYPETSKSRVKVIFIGIGSGF

GLIVLLIGAWWLYKLWKKRKNIKLKKKFFKQNGGLLLEQQLSSGEVNVEKIKLFNSKELE

KATDRFNVDRILGQGGQGTVYKGMLADGRIVAVKKSKIVDGGETEVPLLVYEFIPKGTIY

RYLHEQNEEFPLTWEMRLRISAEIAGALSYLHSAAAFPIYHRDIKSTNILLDDKYRAKVA

DFGTSRSVSIDQTHLTTFVHGTFGYLDPEYFQSSQFTDKSDVYSFGVVLVELLTGEKPVS

LTRSQEARGLVSYFNLSLESNNLFDIIDARVKVEGVTGDILTVANLAKRCLDMNGKRRPT

MKEVAMELEVIHKSVKTSDIQQNHEEVEYVRNEVTCPWDVASTSTGSALDGGTASSIDSI

PLMSN

>RcWAKL18

MDVVQFLLHTTLFLWCVSINTTTVAAPPQQILIAKPNCPSHCGNISIPYPFGIGPGCYAH

NWFEILCNESVSPPKPFFNRTTLNLEVLEISIAGTLKVRNPITVSNNCSDKPIRQGAYLW

GSPFVFSQKNRFTVVGCGVMAGLNCSSDGLTISATCLSQCEIASNASSTCNGLDCSQVSI

PSFLSTFDTSFISTTNPCNYAFLADQDWFQSFTNNLTNISARISDMDYVPAVLEWGLYHS

ILDVFGTSSIASDRSVNCLEYNDASSTYSSSRLECFCAMGFEGNPYLIEGCQDINECLAP

NRQCPVDYACKNHPGYAECYYPKRKSAVKLAFIVIGSVLGLLFLLSVAWWLHKAIKKRKN

IKQMEKFFKQNGGLLLEQQLLSGEVNVDKIKLFNSKELDKATDHFNVDRILGQGGQGTVY

KGMLEDGRIVAVKQSKKLVGGEVGNFINEIVILSQINHRNVVKLLGCCLETEVPLLVYEF

ILNGTLSQYIHHHNEEFPLTWEMRLRVSMEIAGAISYLHSSASMPIYHRDIKSSNILLDD

KYRAKVADFGTARSIAIDKTHLTTRVQGTFGYLDPEYFQSSQFTDKSDVYSFGVVLAELL

TGQKPISLTTSDEWRSLANLFILSMEGNCLFDILDTRVRSDGREEEIVAVANLAKRCLNL

NGRKRPTMKEVAVELEGIQLSVKAHSDAQQNFSEIGYDRTNGMTEAWDVCSTSTGLSCMD

STTGSSLDAQPLLSFKYE

>RcWAKL19

MPKSILITCVLICFAIYHDDAKAEQDCPPASCGDLHDIRYPFRLKSNGTSANNCLNSPAE

LSCEQNRTILKLFDGTYYVLGIDYELETIRVVDPGLLRDTCPFRPNHRLTTYDFTTSPYY

PDYASVTVVFYDCRQKVESPDYIKINSSNSSSSWSSTTYSYIMLGDSVSHLLPSCNITSM

TLSMLHESGNDLSFSFIRDELRKGFLLQWGGLVLPSIPPSSTVLYCYSRFHPLYCFGRFL

KEGFSLTVRMALCINVLVLFLLYKLSWKKILKDDDKDVEEFLDAYKNLMPSRYSYSEIKT

VTNSFKDKLGQGGFGSVYKGELSNGHLVAVKMLNDSKSKGQDFINEVATIGRIHHVNVVQ

LIGFCSEGSKRALVYEFMPNGSLDKYILCDAENNPTLSWDRMHEIALGVARAIEYLHQGC

DIQILHFDIKPHNILLDENFNSKISDFGLARFYPRDQNTISVTAARGTRGYIAPEMYYRT

IGGVSYKADVYSFGMLLMEIAGRRKNLNPDVEHSSQIYFPSWIYEQLEKGKSLEIEDACE

YDTKIAKKMIMAALWCIQLMPADRPSMTKVLEMLEGEEVLQMPPKPLVCPQQMPTEGLPD

DSDDTDFEEISAMPQSATESTSMV

>RcWAKL20

MYHKLFSLLPFASCTMVTKGNTIPSLLCSCLFILICLDHLCSSQKTCPDCGSLKVPYPLS

TNPGRCGDPDYSLKCDPFSKKLYVPALNGSYYLVLQIMASHQRMVVQPSPWLPGNECVTR

DMVVSEGLWLNQSLPFNITSSNTIFLFNCSPRLLVSPLNCTPSSLCHRYLESSGHVDTQR

ALKCESTLNLCCTFVAGGTPSAYKIRLHSSGCKAFRSILHLDVNKPVDQWQDGLEVQWAP

PLEPVCSAQPDCSSTSKCSLTATNGRSRCFCNKGYYWDQVLTTCVKNKRNTKASLSLKVS

IGVISFFVLAVVISIITVKRACRLSEQEKLIKAREGMLKSNDKKSARMFHLKEVKKATNG

FSKDRILGSGGFGEVYKGELGDGTIVAVKSAKVGNIKSTEQVLNEVGILSQVNHKNLVRL

LGYCMEAELPLMLYEYISNGTLSEHLHGKFSTFLEWKSRLRIALQTAEALGYLHSAAHTP

IYHRDVKSTNILLDDEFNAKVSDFGLSRLALPGLSHVSTCAQGTLGYLDPEYYKNYQLTD

KSDVYSYGVVMLELLTSQKAIDFSRGEDDVNLVIYVSVRASNGAIMEVVDQRLLRDKEPS

GNVVGSVKLFLDLALSCLREKKGDRPTMKDVVQELQCIIQNSDQEVLHN

>RcWAKL21

MSECVDHTINGTSSGGYTCRCTTGYDGNPYLSDGCQDINECLLDPNPCKKGKCINLPGTH

TCTCDSGYRLNENKTCVKGGKGKSTVLKVSLGISISMFVLLVGIFWLYCGMKRRKFKQLK

EMYFKENGGEKLREKLASHKGSVETAARIFTSAELKQATNNYHRDKILGEGAYGTVYKGV

LADNKVVAIKKSKIMAKSQTDQFVNEVLVLSQINHRNVVKLLGCCLETEMPLLVYEYVSN

GTLYDHVHKRNGKGPLSLRLRLKIAAETAESLAYLHYSTSMQIVHRDVKATNILLDEKFT

AKVSDFGASKLVPEDQDQLSTLVQGTMGYLDPEYLQSNTLTEKSDVYSFGVVLVELLTSR

RALSLDKPEAERSLANVFVCAVEDGLLKHILDEEVVKDGHFETVEKVADLAKRCLSMKGR

ERPTMREVARELEGLQILPKHSGGGKPDSSPKQTDYLLASPSNAYVVDVKGEGDVGSITT

SIEYDQSMQNQAQVLRPDDAGR

>RcWAKL22

MYQICNLFSPLMNICVLIILWQLKSSSAVDSQYKYCSVPKTCGNVPIKYPFYLDGQQESY

CGYPGFQLYCNGNGHTLLSSGFGSATINTIDYENHTLHLSHDGLFDHAGDCLGYLENFTS

PNGPYELAPNQKEFVLLYYCNSSLVDESFSEYKLGCFDDTRTRTTVLALTKDDPQYGNAL

DKCGKERETVEGVYANVTGTAAGIREGLKAGFVVNWSARNCSLCENSGGKCGTSVGKLTC

FCPDKLHDFFCPEEVGAVAGVALLVVIVWCSRGKLSTYRIIFFWKLQNQKRRTVEAFLRN

YGGLQVKRYSYLEVKKMTNSFKRKLGQGGYGGVYKGKLNDGRLVAVKVLNRPKGDGEDFM

NEVAAISTTSHVNIVSLLGFCFEGSKRALIYEFMPNGSLEKFIFDAKTPQKDHHLGWDML

ETISLGIAQGLEYLHRGCSARILHFDIKPHNILLDENFWPKISDFGLAKICDRKESIVSM

LVARGTAGYIAPEVFCRNFGGVSHKSDVYSYGMMLSEMAGGRKNINVEVEDTSEIYFPHW

IYKRLELDQELGLQSIMNGEDEVRARKMIIVSLWCIQTDPSNRPSMKQVIEMLEGNVESL

QIPPKPYLSSSPKSLALSSPTLVSIQ

>RcWAKL23

MFFFPTFINLYVLIVCLLSETSFAVDSHYQNCSVPKSCGGQNISFPFYIPGLQEDYCGFP

GFQLSCNDNEEGGYPTLQLAGNEYLVHNINYQNRTLVVSNAALSNSNKDVCIPLVQNITF

PTVNYALVPNQKEIVLLYCNSSLVDESFLEYKIGCFEQSSRTTTTTSILALPRDDDQLFD

DVSDKCGRKVVAVAPVEESVSNVGNELGVADALRRGFMLKWLASDCSRCRDSGGKCGFDF

KTYHFSCFCPDRPHAVSCKGAGGLMILVLVVVCCFRRKLSDKSTFFWMNKNQSHQIVPEF

LMDYGPLQVQRYSYLDVKKMTNSFEEKLGQGGYGGVYKGKLNNGFLVAVKILNRSKGNGE

EFMNEVAAISRTSHVNIVSLLGFCFEGSERALIYEFMPNGSLEKFIFDANNPRKDHHLGW

EALDRIALGIARGLEYLHRGCNTRILHFDIKPHNILLNEDFAPKISDFGLAKICNGKESI

VSMMGARGTAGYIAPEVFCRNFGGISHKSDVYSYGMMLSEMVGGRRKIGVEVEDTSEIYF

PQWIYKRLELDQELGLQRIMNEEDKGRARKMIIVSLWCIQTDPSSRPAMKQVIEMLEGSI

GSLQIPPKPYLYSPPKSLAEPAHPSSTLVSLQ

>RcWAKL24

MPPSLAACVFFFFSVLGLISSVHSHLCSPHHQSLCPTFTSTPPFPFSFSPGCGHPSFQIK

CYANHSIITINNLAFSLLHYEPNSTSLLLSPYITNSTIKAKSLSTSNCSSPHFLSIPSRS

INLSSSPFRFSDGSCNRLSVLKPCSPPNLPNCSHCPWECKLIKSPVKLLPGCGSSRSSVP

NQGCQDDVLGYLDNFLQLGIELEWDEAQDSYFSSCRDCEANSGICGFNSSDPSRQFLCLH

TKAQLSPPWISKHNPHRVAILCSVLAVTCLMVMASVSLAIIRSRRRLHSSATEEDPTTLF

LQRHRSASLLPPVFTYEELESSTNKFDTKRKIGDGGFGSVYLGQLYDSRVVAVKYLHKPH

HSAASGKAFSNKCFCNEILILSSIDHPNLVKLHGYCSDPRGLLLVYDYVPNGTLADHLHG

PKSLYRKGSMTWQVRVDIALQTAMVMEYLHFSVVPPVVHRDITSSNIFVERDMRIKVGDF

GLSRLLVFPETTSSSSGYVWTGPQGTPGYLDPDYHRSFRLTDKSDVYSFGVVLLELISGQ

KAVDQRRDKRELALADMMVSKIQMGLLHQVVDPVLIVDGNVTDGVDVAAELAFRCVAAEK

DDRPDAREVVQELRRIRGRIRGISRASSSNVIGADVAKS

>RcWAKL25

MHVMASHTMLIMQLISLAIVGVLVVTITTETITSAEAAAQSLPGCKTHCGNLMIPYPFGI

GDGCYLRPEFNITCDHSTTPPSANFTNYSIRIAHISLAEGELRIMQDVGVECYDTQGRET

DYNWPSLQLPPPYTISDTKNKFFDIGCGSVAIFQGDRTHPGPDEDKSTAGYTMVLCDDLL

GKVLTNSCNGVGCSQFPIPSGLHNFTIILSPIADNTGTWLTRYPCSYSFIVEVAMFTFSP

DTSFDLLNTTSQLPLIVNWGIGDEPCDEKSQNYACKAENSKCVNQSIINGPSGYICQCLP

GYEGNPYLEDGCQGVTVGFLVFFIGISWTSWGIKKRNFIKLKERYFKENGGLLLQKQLFH

HGGSVETTRIFTAEELEKATNNYHESRVLGEGGYGTVYKGILLDNKVVAIKKSKIAAPAQ

SDQFVNEVIVLSQINHRNIVRLLGCCLETETPLLIYEFITNGTLYEHIHKKRSLLSLELR

MKIAAETAGALAYLHSSTSMPIIHRDVKAMNILLDDNYTAKVADFGASRLIPLGQTELET

LVLGTFGYLDPEYLQSNQLTEKSDVYSFGVVLLELITSKVALCTNRCLASIFISAMEEGW

LDQILDDNIVNDGNMEMVKLVANLAKRCLSVKGQERPTMKEVAMELEGMRMMAKHPWRKN

ADFCPEENESLLSSLDANTHVVDIRGDGGSGLLAVQVYNYQV

>RcWAKL26

MALRLSLVAVLLLFSAASTTTTTAAAAQALPADCPDKCGNLTIPYPFGMKKGCYLRKEFF

INCKNTTQGPTAYLMEGNIIVTNISLDDGELQILANVAEDCYNAEGNRTSSFRSWLELPL

PYTISDSKNKFYAVGCDTYATMHGLLWEDEFITGCVSVCYTRGSVDKKSCSGAGCCQTNI

PSGLYNLTVRLNSYFKHSYVLDFNPCSYAFIAEQGKFNFSPTSFEQLNGTEGLPMVLNWA

IGNERDACDGAQKRKDLACRGNYSMCINRPINESGGYLCRCLPDYEGNPYHPDGCQDIDE

CKTSNPCQNGKCSNSPPGNYSCSCHKGYKHDGMNDRICIKKTLKDGSHAYCVSAGFLVSF

IGIAWLWWGNKKRKFIKLKEKYFQENGGLLLQQQLASHGGAVDTTKIFSAEELQEATNNF

HESRVLGKGGYGIVYKGILPDNQVIAIKKSKGCVGSQSEQFVNEVIILSQINHRNVVKLL

GCCLETEVPILVYEFISHGTLHEHIHKKRSSLSFELRLKIAAEAAGALAHLHSSISTPII

HRDVKAANILLDDKYTTKVSDFGASRLVASGEAGIQTVVLGTVGYLDPEYLHSNQLTEKS

DVYSFGVLLAELLTSKVAVSKDEFLASIFVASMEKDCLNQMLDDEIVNEGTIEMVKNVAE

LARRCLRVKGEERPTMREVAMELEGMIMTKHPWGSADHYFPEETEHLLGSPNTSKDYSVN

VDGGGGPGTASGSSMQIDMSMSSYADGR

>RcWAKL27

MALRLSLVAVLLLLLVASTTTTTAAAAQALPGCSDKCGNLTIPYPFGMEEGCYLREEFFI

NCSNTTQPPTAYLRASNIIVTNISLDEGELQISNNVAEACYNEQGNVTSSKDYSLDLPFP

YTISDTKNKFYTVGCDTYSILQGFLGEDEFDTGCISICNGLDIVDQYSCSGAGCCQTNIP

RGLKNTTASLYTINGHEDIWEFNPCSYAFIAEQGQFNFSAASVKKQDKNYRLPAVLNWAI

GNDTDSCAEAQKRKGFACTANSRCANAPINGSVGTCASAYLVMKETPTIQMVAKCTALNP

CNGLNEVCINSLGNYSCLCRNGYKNNGPNGKCIKDNSSDRLLLIILSLGLFVILLVGSLW

MYWGLKKRKFIKLKEKYFTENGGLLLKQKLTSQGGSVETTKLFTAEELEKATNNYHESRI

LGEGGYGTVYRGILPDNQVVAIKKSKVGAPTQTDQFVNEVIVLSQVNHRNVVRLLGCCLE

TEVPLLVYEFITQGTLFEHIHKKKGKGSSLSWELRLKIASETAGALAYLHSSTSTPIIHR

DVKTMNILLDDNYTAKVSDFGASRFIPIDQTQLATMVQGTFGYLDPGYFHSNQLTEKSDV

YSFGVVLAELLTSKVAFSFARAEAERCLAHFFVCSVEDGHLNQILYDGIIKEGDIDQRVI

ENVAHLARRCLRVKGEERPTMREVAMELEGMIMTKHPRGSADHYFPEETTHLLGSPNTSK

DYVVNFDGDGGPDIK

>RcWAKL28

MTGCMSICDNLDSVEQSCSGVGCCLTDIPRGLENYTVRLDSYYNHTYILDFNPCSYAFIV

EEGKFTFSPNTSFNDLSNTEELPMILNWEIGEGPCDEAQKKDDYACKANSKCVNRTISDE

LSGYICHCQPGYQGNPYLPDGCQDIDECKVSNPCTNGRCANSPGSYSCLCDKGYRSNGTN

DQSCIKEIPKNQPETTLLLIVSLSITGGFLVLFVGISWICSGIKKRKFMKLKEKYFKENG

GLLLLQKLANHGGSVETTKIFTTEELEKATNNYHESRVLGEGGYGTVYKGVLLDNKVVAI

KKSKGGTSIQSEQFVNEVVVLSQINHRNVVKLLGCCLETAVPLLVYEFVTHGTLYEHIHR

KRSLLSFELRMKIAVETAGALAYLHSSISTPIIHRDVKAANILLDDNYTAKVSDFGASRL

VPSGQTEIQTLVLGTYGYLDPEYLQSNQLTEKSDVYSFGVVLVELLTSKVAFSNDRCLAS

IFVSSMDEDCLKQILDGAIVNEGNIETAENVASIAKRCLRVKGEERPTMKEVAMELEGMT

IMAKHPWGKNAMFCPEENEYLLGSPSDSNVMDVRGGDRDSSGLTTGMTTGYDSMQIQMLM

PRGDGR

>RcWAKL29

MSIMALQIPLAAVLILLSAASTATTTTAAQALPGCSDKCGNLTVPYPFGMNSDCYLHKEF

FINCSHTTRPPTPYLMNGNLIVTNISVDGELHLLNYVGRDCYNKEDNRTERSTEPELWLV

PPYTISDTKNKFYAVGCDTYAIMRGFRGEDEFITGCMSICNSLASVDKNSCSGSGCCQTN

IPSGLNNLTMSLSSYNNHLDVWDFNPCSYAFIVQQDQFAFSFSSFELLNDTERLPMVLNW

DIGNDTDSCQACGANSMCKNRSIIGSVGYLCECLPGYEGNPYHPEGCQDIDECKANPCEN

GQCLNIPPPGNYSCSCYEGYKHDGMNDKICIKDDSKRDSKIVLLLMISLGVSVGFLVLFL

GISWISWGMKKREFIKLKEKYFKENGGLLLLQQLASHGSSMKTTKIFTAEELEKATNNYH

ESGVLGEGGYGTVYKGILPDDTMVAIKKSKGAALTQSDQFVNEVIVLSQINHRNVVKLLG

CCLETEAPLLVYEFITHGTLYEHIHKKRSSLPFELRMKIATQSAEALSHLHSSISTPIIH

RDVKTSNILLDDDYTAKVSDFGASRLVPSGQTDIQTLVLGTFGYLDPEYLQSNQLTEKSD

VYSFGVVLVELLTSKVPVSKDRCLTSIFLASMEEGWLNQILDDGMVNEENIETVKKVANL

AKRCLRVNGEERPTMKEVVKELEEMSVMAKHPWGFNANFCKEETEYLLGSFNSDAYVVGN

GEGDCSSSGLTSGTTTAYDGMQIEQLMPHDGGR

>RcWAKL30

MVLNWAIGDEPDPCDVAQIRQDFVCKQNSKCLNPTNRNGYICRCLEGYEGNPYHPDGCQD

IDECKDPNLCNHGECKNLAGSYTCLCSKGYKIDSMNDKSCIKENPKNNSKMTLLLIISLS

VSIGLLIFFVGISWICCGIKRRQYTKLKEKYFKENGGLSLLQQLASHGGTVETTKIFSTE

ELEKATNNYHESRILGEGGYGTVYKGILPDDKVVAIKKSKGGAPTQSDQFVNKVIVLSQI

NHRNLVKLLGCCLETEVPLLVYEFITHGTLYEHIHKKRSSLSFELRMKIAVETVEALAYL

HSSTSNPIIHRDVKAENILLDDAYTAKISDFGASRLIPSGQTEIQTLVLGTFGYLDPEYL

QSNQLTEKSDIYNFGVVLVELLTSKMALSKDRCLTSIFISSVDEDCLNPILDDDIVNEGN

IESVKNVASLAKRCLRVKGEERPTMKEVAMELEGMRITAKHPWGTNVGFCPEENEYLLGL

LDSDAYVLDVRGGGGSSSGLTTGTGYDSMQIQMLMPYGDGR

>RcWAKL31

MSIMALQFPLAAVLILLSAATTTTTAAAVQALPGCSDKCGDLTIPYPFGMNESCYLGDMF

RINCSQGAQPDQPPTAYLGDTNIIVTSISIDVGELRILSHRGEDCYNETGNLTYRNRPHF

WMGPPYTISDTKNKFYAVGCDTYAIMRGFRGEEEFITGCVSVCNSFSSVDKNSCSGAGCC

QTSIPSGLHNRTVSLDSYFNHSYVWDFNPCSYAFIAEQGQFNFSSSSFEQLNDTERLPMV

LNWAIGNDTDPCHEAQKREDFACTANSICKNRPINGSVGYLCECSPGYEGNPYHPDGCKD

IDECKALPCENGQCFNKPFPGNYSCSCYKGYKNDGMNDKICIKDDSKRHSKIVLLLMISL

GVSMGFLVLFPWICWGMKKREFIKLKEKYFKENGGLLFLQQLASHGSSMKTTKIFTAEEL

EKATNNYHESRVLGEGGYGTVYKGILADDIVVAIKKSKGGALTQSDQFVNEVIVLSQINH

RNVVKLLGCCLETEVPLLVYEFITHGTLYEHIHKKGSSLPFELRMKIAAQSAEALSHLHS

SISTPIIHRDVKAANILLDDKYTAKVSDFGASRLVSSGEAGIRTVVLGTIGYLDPEYLQS

NQLTEKSDVYSFGVLLAELLTSKVAVSKDKFLASIFVASMEKDCLNQVLDDEIVNEGNIE

MAKNVAKLARRCLRVKGEERPTMKEVAMELEGLIMTKHPWGSADHNFPEETKHLLGSPNT

SKDYSVNVDGGGGPGTASGSSMQIDMSMSSYADGR

>RcWAKL32

MALVHGKMLLMKLTSVILAAATILLAAAAANKPLPPPQAKPNCPDRCGNLTIPYPFGIGD

GCYIPLKGQEKQFELACDNSTNPPSLNWTYSTFRVTDFILAEGQLQVMNPISKDCYNENG

SNVEYKVPGLNYYPPFSISGRKNNFVAVGCDTSAIFRGFRGEQVFFTGCMSLCPNISTVD

QDSCSGVGCCLQNKIPEGLKNLSVILNSYYNHMDIWAFNPCSYAFVVQDGYFNFSGKRSF

EQLKNMEQIPMIINWQIGNETCEGAKKNAVDYACKEYSEYIDECLAEINPCGNGTCQNSP

ESYSCKCNKGFKNDGPQKCIARPTANNTPLKISLGICIGFFVLLVIIFGLYREYKRRQFN

QMKKKYFEANGGPKLQLQLKQLASQKESLATQIFTAEQLKKATKDYDENEKIGEGGYGIV

YKGVLDDKREVAIKMSKMTAQVKSDQFINEVIVLSQISHRNVVRLLGCCLETETPMLVYE

FVGNGTLYDHIHKKKGQPLSFAQRLKIAAETATALSYLHHSTTMQIVHRDVKATNILLDE

KLTAKVSDFGASKLVPDDKTQLSTLVQGTMGYLDPEYLQSNTLTEKSDVYSFGVVLVELL

TSENAVRFDKPEAERNLANVFVSIIEEKGLRALGPILDDEIVKDGNWEIIEKVANLAKRC

LSVKGDERPTMKQVERELDEILRTLAKQPGGKKLYASSSKETDTSLESHSNDYNVEISNE

GDGGSTGIISSAEYDASMQNQAQKPSGYGR

>RcWAKL33

MLGVVVPILVVVVLAAATSSSTTSAQALPNCPDRCGDVKISYPFGTVKGCYLRDEFFINC

TSAEGNASVAKLTSPPSSSPTSPLTDLIVTDISLDNAELRILSSVYQDCGDRKETLNKWL

RLPSPFAFSTKNIFFAVGCATFAKYRGYQYRPSGRILGPDDDQAYNKSGFAVTQCDDSFG

TTCSGNGCSQSSIPSGLQNFTVESLLSISINGYIPNQGKWYSPYLGCNYGFLAEEGNFSF

SPNISFAQLQKYRRLLVPTTTVINWQVGDEPCEEAQKSKGSYACKGNSTCVNWSNVNEGG

YGGWHLGYICRCLPGYQGNPYLADGCQDINECQSSDRCRTEELCVNLPGQYKCDPNQLKT

SLRFLISFGIFASVFVIVVGGLGSIYWEMKKREYMILKEKYFEKNGGLLLQQLANHHGVE

MAKIFTAEELEKATNNYHESRVLGEGGCGIVYKGILQNDRTVAIKKPKVGNSSHSTTPTH

CTPSQQFVNEVIILSQINHINVVRLLGCCLETSVPILVYEFITNGTLFKHLHGDKRGKRS

SPPLFWDLRLKIATETAGALAYLHSSTSTPILHRDVKTMNILLDENYTAKVADFGASQLI

RPLDEKQMTTFVQGTLGYLDPEYFHSDEFTEKSDVYSFGVVLVELLTSKKAFSFARPDAE

RNLSKLFVSSVEDGRLKEILDNDMVDEANINDTMVLENVAYLAKRCLRVKGDERPTMKEV

AMELECLYKGLSSIYDMTHKHYFICQDETDEDLLGSSTSNTADVAADVL

>RcWAKL34

MALHGRPLIMQLISLVMVAIEAASDQALPPQALPGCPGRCGNLTIPYPFGIGDGCYLRQR

FNITCNESAHPPTALLADGSIIATNISLDEGELQFWNTIAHDCYDKQGNQTGSTSNSPSL

QLNPPFTISGTKNNLIAVGCDTSAIFKGNFQYPYDEARYILTCMSICYNLDSAVNGSCSF

GSSIGCCQTGLPNGLKNFTLTLGSFSEYKDVIWNFKPCSYAFIVEEGEFTFFPNTSFQEL

SKNQQLPVILNWEIQDGGCAEAQKRHDYACKANSRCVNRTIDIRTEPSGYYCQCLPGFEG

NPYLPDGCRGVSLGFLVLLVVIIWIHWEMNKRRIIKLKEKHFKENGGLMLQQQLARHGGR

VDTTRIFTSEELEKATNNYHESRILGEGGYGTVYKGTLPDNTMVAIKKSKVAARTQHDQF

VNEVILLSQINHRNVVRLLGCCFETEVPLLVYEYITHGTLFEHLFNNKNGKRSSLSWELR

LKIASETAGALAYLHSSTSTPIIHRDVKATNILLDENYTAKESDFGASRFIPLDQTQVAT

LVQGTMGYLDPEYMQSNTLTEKSDVYSFGVVLAELLTSKVAVSFARPESERSLAYLFVSS

IEKQCLIQILDADMVNEGNVEMLEQVANLAKICLRVKGKERPTMKEVAMELEGMRTTAKH

PWKNADFYPEETEYLLGSPNSKSYIEGKGEVSSSGSVSRYASMQNEMLKSYDDGR

>RcWAKL35

MQVSFAARNWLFSLSCLTLSDINECIASTDPCNNGTCKNSDGDYTCTCNFRNENPKSCVQ

NTPISKNKSLKVSLGVSLSFLGVLLVTFWIYCGLKRRKFKILKEKYFKDNGGFLLQQKLE

NFDGPQAAKIFTREELKKATNNFHDSLKIGEGGYGFVYKGTLSNGNEVAIKVSKSSAPMT

QSNQFINEVIVLSQINHRNAVKLLGCCLETQTPLLVYEFVSNGTLYEHIHKKNGKGPLSF

GLRMKIATETSASLAFLHYSTSMQIVHRDVKAANILLDENFTAKVSDFGASKLSNTLTEK

SDVYSFGVVLVELITSQVAISYQKPEAERSLAKIFVTSVEENRLDQILDHEIIKEGSFET

AEQVAHLAKSCLSLKGSDRPAMKEVAMKLEGVLQVMAKHPWGKADASPKETDYLQLASHS

KAYVVDVFFFF

>RcWAKL36

MIMFRTASLLFSVHTVSLLFLQLSSRLQTSWATSECTPSCGNIKISSPFRLQGDPGQCGN

KSYEISCEADGTSQSHQAILYLFSGKYYVQAINYNNYTIRLVDAGVHKTKGNHFSNPVYS

LSVFNFTSSSVDPYVIKYTSSYDPYFLSYPYRVYPEPFQYGGGDPLTVSLIFLSCTNQMN

HSDLFVETAPCIKNTGIHSSSDASLSTVYSYFMLGRLNDDGSPEDLSPAEWGLSCKITLM

ARVSPPPTPTDKYSKSCQGIYKQLAYGFQLSWVRYGCRENCGPHNICMLTHDNITRFECY

KGDGQFVEIIRSIIHKLVPYPVRAAFDYVMLGPYKLLTLFVVFYGGIFAAAKLSIGFPFV

IAILIYKWRRRHLSMYDNIEDFLQSNSNLMPVRYSYSEIKKMASGFKDKLGEGGFGTVYK

AKLCSGRLVAIKILSKSKTNGQDFINEVATIGRIHHVNVVRLIGFCVDRSNRALVYDFMS

NGSLDKYIFSQQGAISLSCEKIFEIAVGVARGIQYLHQGCDMRILHFDLKPHNILLDENF

TAKVSDFGLARLYPLDNSIVSLTAARGTIGYMAPELFYKNIGGVSYKADVYSFGMLMLEM

AGRRKNLNAAIDHSSEFSHIYFPTWVSDQLNQGKEIEIGDATEDEMKIIKKMIIVALWCI

QMKPIERPSMSKVVEMLEGEIESLQIPPKPFLYPQQMPVNDVEDNLSTTCASRMTQSTEI

NLSADANLVNLYLSISGCKGRETSL

>RcWAKL37

MPQIFLSPSPIFTVFSFFFVFLCSVNFTLSLSSNSNLPNCTNTFNCGTLHNLTYPFTGGD

RPSYCGPPEFHINCLNDSPELSIMSLTYRVLALDQAAQTLSLARTDLWNENCTSDLANSS

ISSGFFSFSDADNVDITIFYGCNSSSIVPKPENWFHCNINLTSNDAYYLIGPVPLDPIMS

TFKCFIGITVPILKLTAAKLVANRSLFKQAIMGGFNANYTNPYLRQCNKCLNVNGECGFD

SDSNRPVCICGGRLCDPAGKNNQIPIGLAVGGAILFGIFVGFYAYFVIQRKKKKRAAEAQ

SKEIPTPLTSKSTVTPSTNLSQSQSIPSYPSFNSKSRSDLGKASTYFGVQVFSYAELEEA

TENFNPAKELGDGGFGTVYYGKLHDGRVVAVKRLYENNFKRVEQFMNEVEILTRLEHQNL

VKLYGCTSRRSRELLLVYEYISNGTVADHLHGKRMESGFLSWPVRLSIAIETADALAFLH

RNDVIHRDVKTNNILLDDDFCVKVADFGLSRLFPNDQTHVSTAPQGTPGYVDPEYYQCYQ

LTDKSDVYSFGVVLIELISSLQAVDTNRHRHDINLANMAINKIQNHLVNELVDPLLGYDK

DYSVRIMATAVAELAFRCLQQEKDMRPTMEEVLEGLKAIQNEDLGAQENGEAEAVVLDIG

ADEVGLLRNLPPPLSPDSVVTDKLILKRWDRTM

>RcWAKL38

MELVNPLGGCCQKSITIKGLTNYAMSYGSRKEVIRNRNSSILAMVPPTCAYALVVEADTF

EFSSLDLQQGIHNRDAVPMVLNWVVGNYNTCQEAQSSSSTPSSNITYACRAKNSECHDST

SGPGYLCKCREGYQGNPYILDGCQGIFVAFGVLITIQAVILVVLIRLAKLHVNYFTDIDE

CERKRELINPCQLSKMHQQSSKCLLHLSRWIQRKWQEKRNWLQHQYLQLILAFGICISIL

VLLGVRFRICWASKKRKLVQLKMEFLKQNGGILSRQLLSGERALEMIKIFSAEELKKATN

SYNEGVVLGKGGNRRVYKGLLRGKKVVAIKKSKSITPMLSKLLGCCLETEVPILVYEFIT

KGTLSSHLHSSSIRLSWQIRLKIAKEVAAALAYLHSETSIPIIHRDVKSANVLLDDNFTA

KISDFGASKLTPIGQTQITTLVQGTYGYFDPEYFCSGILTEKSDVYGYGVVLAELVTGKE

ALFWNSSGTRINLAFYFVSSVKEVRLVEILDDQVARR

>RcWAKL39

MSSARLYLLVALLLLFFFNFHGSKANFLASTNCPIYRCDNELDFHYPFWKIEDSTAHQHC

GYPGFGLTCSDSGEPILTLPNDSFIVKDINFPTSTITLVDIDVVNQTCPRARHSMSVGTL

PLDYSPLDVNLSFYFNCTSFPDPVVPPITCLGSHGTKKSYVITEGEEPYGFEWSEYCAEN

VVVTVKKTEEITRSIGELIGAFGLAMNNGFVLNWTMDKECGSCETNGGFCGYNNTAPEIL

CFCKDGSIGTTSNGLCKKLGTGILVFVILFRGISLISENDNGDAEAFIQNIGPLAVKRYI

FSDVRKMTNSFKDKLGQGGYGDVYEGKLLNGCRVAVKVLKASKGNGEDFVNEVASISRTS

HVNVVTLLGYCIAGKKTALIYEFMPNGSLEKFIYKDSNLLTITPHLELEKLFQIAIGIAR

GLEYLHRGCNTRILHFDIKPHNILLDENFFPKISDFGLSKLCTRKESIISMLDARGTIGY

MAPEVFSRNFGRVSAKSDVYSYAMMILEMVGGRKNVDARVSHTSEIYFPDWVYEHLEQGS

NFGLLSAATEEEKELAKKMFLVGLWCIQTKPSDRPSMSKVIEMLEGSTEALQIPPKPVLS

SPVRSPAESSTLSVVSVLD

>RcWAKL40

MFKPKPNHLFRRAPFFFIFYLFRLPSLHNEDDPQYTQCKQPFNCGNLTDISYPFWGGPNR

PQECGRLGFELTNCEDETQLPHIVIEKLDFHVSNINSQDLLHTMTIARSDLWDNPCTDLL

VNTTLDYNRFSYVQTVRNLTLYYGCLPQNEPVLNNFTCKIDGTTKERNISYYVDDSLSRV

DVPGGPTCSTKIIVPTFWEGFDVMPDNATEEVEKVLKQGFQVEYRADWDLCRLCMNSNGT

CGSNATTDSFLCLCGDRPSNSTTCPITPIVHGTKFNWKTKVFIGVAATIAGSCILLFVIL

FRWTSLIRENDNEDAAEAFIRNIGPLAVQRYKFSDVRKMTNSFKDKLGQGGYGDVYEGKL

LNGCRVAVKVLKESKGNGEDFVNEVASISRTSHVNVVTLLGYCFEGKKKALIYEFMPNGS

LEKFIYKDSNLLTTTPHLELEKLFQIAIGIARGLEYLHRGCNTRILHFDIKPHNILLDED

FCPKISDFGLSKLCTRKESIISMLEARGTIGYIAPEVFSRNFGRVSAKSDVYSYGMMILE

MVGGRKKVDARVSHTSEIYFPDWVYEHLEQGSNFGLLNAATEEEKELARKMILVGLWCIQ

TKPSDRPSMSKVIEMLEGSTEALQIPPKPVLSSPVRSPPESSTVSVVSVLD

>RcWAKL41

MSSARLYLLAALLSLLLFNFHGSKAHFVPSTNCPIYNCGKDLKFHYPFWKIEDSTAGQYC

GYPGFGLNCSESGEPILSLPNNDSYHVKDINFIDSTITLVDIDVFDQQCPRARHSISVGT

LPLDYSPLDVNLSFYFNCTSFPDPVVPPIICLGIWGTKMSYVFTEGEQSDGFEWSESCEE

NVVVTVMKSKKLYIDKLIGAFGGLMNQGFVLNWTTAKECASCESGGGLCGYNPTKADEFL

CFCKYSDGLCEKKGTRFNWRLKVVIGVGTGILVFVILFRGISLIRENDNEDAEALTQNIG

PLAVKRYKFSDVTKMTNSFKDKIGQGGYGDVYEGKLLNGCRVAVKVLKASKGNGEVFVNE

VASISRTSH

>RcWAKL42

MSSPISYLLAALLSLLLFNFHGSKAHFVPSTNCPIYNCGNDLKFHYPFWKIEDSTAGQYC

GYPGFGLNCSESGEPILSLPNNDSYHVKDINFIDSTITLVDIDVFDQQCPRARHSISVGT

LPLDYSPLDVNLSFYFNCTSFPDPVVPPIICLGIWGTKMSYVFTEGEQSDGFEWSESCEE

NVVVTVMKSKELHIDKLTGAFGGVMKQGFVLNWTTAKECASCESGGGLCGYNPTKADEFL

CFCKYSDGLCEKKGPKLSRRLKVVLGLCSVIGTIAVMCFIFFIWRRRNRNRYGPSSYASR

IIYSKSYSRSDTEKGSAYTGVHVFTYNELEQATNYFDSSKELGSGGFGRVYYGKVRDGRA

VAVKRLYETNCRRVEQFLNEIEILARLRHRNLVLLYGCTSRHSRELLLVYEYVPNGTLAD

HLHGGKAKPGALPWHTRINIAVETGSALSYLHASEIIHRDVKTTNILLDNNFCVKVADFG

LSRLFPTDVTHVSTAPQGTPGYVDPDYNQCYQLTMKSDVYSFGVVLVELISSLPAVDITR

HRQEINLSNMAINKIQKHALHELVNPSLGYESDFKVRKMINAVAELAFRCLQNETDMRPS

MVDVENELKRIQSDDFSKEKAEEIDISADDVVLLKSGPLPPSPDSVTINWVSMSSTPNDS

TA

>RcWAKL43

MNPFHLCVFSIIFTTIVSLAFSVDSSYYEACKPQTCGNGPNISYPFWLSDQQESYCGYPS

FKLACKDKNPTLTISDDDYIIKEIFYSNHSFLVDNGAVYEDECPLPLHSFRLDRTPFNYS

SNHVDFSLFYNCSQEPVNNMLTYSITCESNSTLHSFATIHKEVLQYMNRTLLDSCESSVV

LPLDYHEAGGVNTWLEMNYTEILKMGFVLNWTAQNCSNCETSGGRCGFDNNEFVCFCSDR

PHEKTCDDGSKFDWKWKVVVGICSVIGTIAVMCVIFFIWRRRNRNRYGPSSYASRSIYSK

SYSRSDTEKGSAYLGVHVFTYKELEQATNYFDSSKEIGDGGFGTVYYGKVRDGRSVAVKR

LYETNYKRVEQFMNEIEILARLRHQNLVLLYGCTSRHSRELLLVYEYVPNGTLADHLHGG

KAKPGALPWHTRINIAVETASGLSYLHASEIIHRDVKTTNILLDNNFCVKVADFGLSRLF

PTDVTHISTAPQGTPGYVDPDYNQCYQLTAKSDVYSFGVVLVELISSLPAVDITRHRHEI

NLSNMAINKIQKHALHELVDPSLGYESDFKVRKMINAVAELAFQCLQSETEMRPSMVDVE

NELKRIQSDDFSKEKAEEIDISADDVVPLKSGPLPPSPDSVTINWVSKSSTPNDSTG

>RcWAKL44

MMLKRVWLCTCVIAICFVISQEAKAEKLCQPASCGDLHDIRYPFRLKINGSDQNCLSSPA

ELTCDEHNRTTLNLYNGTYYVRAINYDNSTIQVVDPGLLRDSCPFRPLYLLTGDNFSHTD

IYQPTGAYDFVSFLDCLQPVKSRDYAKVNCSSSLSTTSYSYIMVGYRVGDLPPSCYMVTA

SLAMLQQPWGHRSFSLTRQQLERGFDLSWGKYICEKHCQASDAFCSFSNYRWECYDNCYS

PFTTIYCFQDYLKRACSMVIRTVIGIIVFLGFWSYKLYSKMFVKDDAVEEFLHSYKNQMP

RRYSYLDIRKMTTDFKDKLGQGGFGSVYKGELSNGHLVAVKMLSGSKGNGQDFINEVATI

GRIHHVNVVQLIGFCSDGLKRALVYDFMPNGSLDNYIFPKKKKALSLSWNRMQEIALGVA

HAIEYLHQGCHVQILHFEFLILNRTIFFLMRTSLQKFRTLGLPGYIAPELFYRTIGSVSY

KADVYSFGMLLLEMAGRRKNLNLDAKSSSQIYFPSWIFGQLEKGLCIEIEDACESDKKIA

KKMIIVALWCIQLTPVDRPSMSKVVEMLEGEVQVLQMPPKPFYCPQDLPKADLPGESDFE

EVSTQQHSAIELPSFQSFGNVIE

>RcWAKL45

MQWLVQHMIFILLLYKWRICTASLSPPKIAKENCTSRCGGVSIPYPFGIGPKSHCYFDEW

YELECNLSDPVAKPFLKGLQLEVLTIFVENSTLQVTSPITYFSCKGKQSRPAANLTGSPF

QYSVFNSFVAVSCGFLVSVLSGSNHTLGGCTSTCGSDRSRGLCFVGDNCCEIVISTDLIA

NFSAFRQEQDEVRTNATDCEDCAFLVYDKWFDNNVSDSDGLDLTAIKGMEVVPVELEWSL

SLAKNNSLIKSFEALDRFPEVRRPNDPTPSCIVSFDDSSFRWYQCSCPAGFQGNPYLLRP

CQDIDECKDTNPCVGSDLNTTNIWNISDAKCQNTIGGHACYSNRTGQTCELFGENTKARC

FYTSRHHSQLKPILSGLGASIGLLVFLSAAWLVYKVAKKWKSTKRKEMFYKQNGGLLLEQ

QLSSSEINVERIKVFKSKELQSSTDNFNVDRIIGQGGQGTVYKGMLTDGRIVAVKKSKMI

DNANLSEFINEVVILSQINHRNIVQLLGCCLETEVPLLVYEFIPNGNLFQYIHGQTKEFP

LTWEIRLRIATEIAGALSYLHTSASFPIYHRDIKSTNILLDDKYRAKIADFGTSRLVAID

QTHLTTNVHGTFGYLDPEYYQSSQFTEKSDVYSFGVVLVELLTGKKPITRSAEDEMYKSL

ATYFIISLQEDSLFDILDARVVNEGSKDGIMVVATLAKRCLNLNGRKRPTMREITAELEA

VQLSEKLANAHQQSCERFEFGQDYRIEQGDDVVSSSTMSTWEGAPPPSSVELPLL

>AtWAK1

MKVQEGLFLVAIFFSLACTQLVKGQHQPGENCQNKCGNITIEYPFGISSGCYYPGNESFS

ITCKEDRPHVLSDIEVANFNHSGQLQVLLNRSSTCYDEQGKKTEEDSSFTLENLSLSANN

KLTAVGCNALSLLDTFGMQNYSTACLSLCDSPPEADGECNGRGCCRVDVSAPLDSYTFET

TSGRIKHMTSFHDFSPCTYAFLVEDDKFNFSSTEDLLNLRNVMRFPVLLDWSVGNQTCEQ

VGSTSICGGNSTCLDSTPRNGYICRCNEGFDGNPYLSAGCQDVNECTTSSTIHRHNCSDP

KTCRNKVGGFYCKCQSGYRLDTTTMSCKRKEFAWTTILLVTTIGFLVILLGVACIQQRMK

HLKDTKLREQFFEQNGGGMLTQRLSGAGPSNVDVKIFTEDGMKKATNGYAESRILGQGGQ

GTVYKGILPDNSIVAIKKARLGDSSQVEQFINEVLVLSQINHRNVVKLLGCCLETEVPLL

VYEFITNGTLFDHLHGSMIDSSLTWEHRLKIAIEVAGTLAYLHSSASIPIIHRDIKTANI

LLDVNLTAKVADFGASRLIPMDKEELETMVQGTLGYLDPEYYNTGLLNEKSDVYSFGVVL

MELLSGQKALCFKRPQSSKHLVSYFATATKENRLDEIIGGEVMNEDNLKEIQEAARIAAE

CTRLMGEERPRMKEVAAKLEALRVEKTKHKWSDQYPEENEHLIGGHILSAQGETSSSIGY

DSIKNVAILDIETGR

>AtWAK2

MKVQEGLFVVAVFYLAYTQLVKGQPRKECQTRCGNVAVEYPFGTSPGCYYPGDESFNLTC

NEQEKLFFGNMPVINMSLSGQLRVRLVRSRVCYDSQGKQTDYIAQRTTLGNFTLSELNRF

TVVGCNSYAFLRTSGVEKYSTGCISICDSATTKNGSCSGEGCCQIPVPRGYSFVRVKPHS

FHNHPTVHLFNPCTYAFLVEDGMFDFHALEDLNNLRNVTTFPVVLDWSIGDKTCKQVEYR

GVCGGNSTCFDSTGGTGYNCKCLEGFEGNPYLPNGCQDINECISSRHNCSEHSTCENTKG

SFNCNCPSGYRKDSLNSCTRKVRPEYFRWTQIFLGTTIGFSVIMLGISCLQQKIKHRKNT

ELRQKFFEQNGGGMLIQRVSGAGPSNVDVKIFTEKGMKEATNGYHESRILGQGGQGTVYK

GILPDNSIVAIKKARLGNRSQVEQFINEVLVLSQINHRNVVKVLGCCLETEVPLLVYEFI

NSGTLFDHLHGSLYDSSLTWEHRLRIATEVAGSLAYLHSSASIPIIHRDIKTANILLDKN

LTAKVADFGASRLIPMDKEQLTTIVQGTLGYLDPEYYNTGLLNEKSDVYSFGVVLMELLS

GQKALCFERPHCPKNLVSCFASATKNNRFHEIIDGQVMNEDNQREIQEAARIAAECTRLM

GEERPRMKEVAAELEALRVKTTKYKWSDQYRETGEIEHLLGVQILSAQGETSSSIGYDSI

RNVTTLDIEAGR

>AtWAKL10

MSSNCSCSLLSLFSLLLIIDLTVASSCPKTCGGIDIPYPFGIGTGCYLEKWYEIICVNNS

VPFLSIINREVVSISFSDMYRRFFNVGYGSIRIRNPIASKGCSSGGQEFGSLLNMTGYPF

YLGDNNMLIAVGCNNTASLTNVEPSIVGCESTCSTNQDIPINDYLGVLYCNARYGDSEYC

KNISIMNDTSCNGIGCCKASLPARYQQIIGVEIDDSNTESKGCKVAFITDEEYFLSNGSD

PERLHANGYDTVDLRWFIHTANHSFIGSLGCKSIDEYTILRRDNREYGIGCLCDYNSTTT

GYATCSCASGFEGNPYIPGECKDINECVRGIDGNPVCTAGKCVNLLGGYTCEYTNHRPLV

IGLSTSFSTLVFIGGIYWLYKFIRRQRRLNQKKKFFKRNGGLLLQQQLTTTEGNVDSTRV

FNSRELEKATENFSLTRILGEGGQGTVYKGMLVDGRIVAVKKSKVVDEDKLEEFINEVVI

LSQINHRNIVKLLGCCLETDVPILVYEFIPNGNLFEHLHDDSDDYTMTTWEVRLRIAVDI

AGALSYLHSAASSPIYHRDIKSTNIMLDEKHRAKVSDFGTSRTVTVDHTHLTTVVSGTVG

YMDPEYFQSSQFTDKSDVYSFGVVLAELITGEKSVSFLRSQEYRTLATYFTLAMKENRLS

DIIDARIRDGCKLNQVTAAAKIARKCLNMKGRKRPSMRQVSMELEKIRSYSEDMQPYEYA

SENEEEKKETLVDVNVESRNYVSVTAASSQYSIATTSSSRSDVEPLFPR

>AtWAKL22

MKRRRLFFSVLLSILTLFINGPLITTAQSPPSSSTSCNRICGGIEIPFPFGIGRRDCFLN

DWYEVVCNSTTSGKSLAPFLYKINRELVSITLRSSIDSSYGVVHIKSPVTSSGCSQRPVK

PLPLNLTGKGSPFFITDSNRLVSVGCDNRALITDIESQITGCESSCDGDKSRLDKICGGY

TCCQAKIPADRPQVIGVDLESSGGNTTQGGNCKVAFLTNETYSPANVTEPEQFYTNGFTV

IELGWYFDTSDSRLTNPVGCVNLTETGIYTSAPSCVCEYGNFSGFGYSNCYCNQIGYRGN

PYLPGGCIDIDECEEGKGLSSCGELTCVNVPGSWRCELNGVGKIKPLFPGLVLGFPLLFL

VLGIWGLIKFVKKRRKIIRKRMFFKRNGGLLLKQQLTTRGGNVQSSKIFSSKELEKATDN

FNMNRVLGQGGQGTVYKGMLVDGRIVAVKRSKVLDEDKVEEFINEVGVLSQINHRNIVKL

MGCCLETEVPILVYEHIPNGDLFKRLHHDSDDYTMTWDVRLRISVEIAGALAYLHSAAST

PVYHRDVKTTNILLDEKYRAKVSDFGTSRSINVDQTHLTTLVAGTFGYLDPEYFQTSQFT

DKSDVYSFGVVLVELITGEKPFSVMRPEENRGLVSHFNEAMKQNRVLDIVDSRIKEGCTL

EQVLAVAKLARRCLSLKGKKRPNMREVSVELERIRSSPEDLELHIEEEDEEECAMEINMD

DSWSVDMTAPASLFDLSPKLDVEPLVPQRTW

>GhWAK7A

MGLIRSVVLIVQQVILLNAISAVKATAAVAYQANSSCQSRCGEVSIPYPFGTGGDCNVSK

HFFITCNTSFTPSKAFLTTSSIEILDISLNGQLRILADGSYDCYNNSGATRNFTYWLQLG

KFFINNTRNKFTAIGCDTYARVEGFSGQRYATGCLSLCNNINDVSNGSCSGIGCCQTSIP

KGVKSYNITIESYENHTDVLPENPCSYAFVAENDNYTFSTSDLRGFDFKDKLFPVTLDWT

IGKTSCKEAKMDTKSFACKKNSKCIDSEHNSGYICKCFEGYEGNPYLPNGCQDINECVTM

SPCNGTATCINLNGTYDCLCPPDYKGDGKKNGTGCSLPNKDQSKRSLLIDVALGIGVGFL

GILLGIVLLCWMLKQRQISKLKRVNFQQNGGILLREQLSKRQGYREEVKVFTVEELEKAT

NNYHESRILGQGGQGTVYKGILADNRIVAIKKSIIGDPSQVERFINEIMVLYQINHRNVV

KLLGCCLETQVPLLVYEYITNGTLFHHLHDDDAALDLLWETRLRIATETAEALSYLHSAV

SIPIIHRDIKLANILLDNNYNAKVSDFGASRLIPSDEAQITTIVQGTFGYLDPEYMHTSL

LTEKSDVYSFGVVLMELLTGQKVVCFKRPEEKRVLAMYFVSLMKEDNLLDILDPRVLTDK

NVEQLKEVAALASRCVRMKREERPTMKEVAHELAGLQAMPKHPWSKSKLQGEESEYLLGD

MCSTYTDGATSSSMGYDSINNKITFELEGAR

>OsIRBB4 Xa4

FTIICKYSRPYYRGAEIVNISVEAGEMRVYSPVVSQCYNSSNTTDSDGFEFLRLNITNTP

FLVAPERNEFTAIGCATLAWLWGRDDGSYLTGCISTCASLATAAKDRDPCTGLGCCQVPS

IPANLSVLNISLGTGIANVAWEESPCSYAFVAEKHWYNFNRQDFERDGKSFEHRDEKMVV

PTVLDWAIRKNGSCPSTGQGAPACKSEHSECVNATNGKGYLCNCSRGYAGNPYRDDGCKN

INECKEPSITCYGGSTCQDTDGSYECKCQFGYRGDGRKNESQKGRCQPIIPAAIANAIAI

VCIVIVLLGLFWLPKRWKRRVFFDNNGGRLLKDMDIIVFTEKELNKITNKKRTKIGEGAF

GEVYKGNHNNQPVAVKYSIAKNMTQTHYKDVVESINQNVFQTVFRQSKVPPSTPGQNAVV

NEIKVQLQIRHPNIVRLIGCCMETEVPMLVFEFIPNGSLETVLHGIDRCSLSLQQRLDIA

IGSAEALAYMHWHGHHQIIHGDIKPGNILLGDNLMPKVSDFGSSELTLKVKRAGKWNVYA

DMNYIDPVYIKTGDFTDKSDVYSFGVVLLELITRKKAKYDDRSLPVEFVSHYEDEDTRRK

MYDQDMLPTEASHPHCMECLDRMADIVLRCLENEVGKRPTMAEVLEELKKLLPLLTTTPV

ELV

>OsWAK14

MSSSLLVAACAVSFVLLCSAATSPASAAVYGVGGGLLSIPSNDSLAHCPSRCGDVGIDYP

FGIAPGCFREGFELICRNTAKTPKLFLGDGTTEITDLGYRYVLAQIYFNITVRPGTDTYN

ISWVAPTEGITIDHYNTFYVIGCNFDATLFEYGTEDLIGSCMSRCDGEKAPIGGPCNGMG

CCFIELPRVLRGFQSTIILRSDGIPVAQTDPVHPGIMAFMSSDYYISNTSDLFLGWTNTS

NVEGTVLSFATIDQPSCERARMNNTSYACSPGSNCRNVSSGGYHCYCSGYEQGNPYLLDG

CTDYNPKYKEHCSTSCGDMKIPFPFGVEEGCFANERFRLNCTEGNLTVCELGEAQYHVTA

VSLDDGTLTVGNMMNDTNYEKEAIIVQTTDTGRDYSFSGPVEDRFDLSMEYAIVIRWAVT

NLTCEVAVQKNTTYACRSSHSYCLNVTHRKEFMGYRCKCSPGFEGNPYIEDGCTDINECL

LPNYCNGTCQNLLGNYTCTSCPHRKEFDPIKKKCVTSAKQRNLLLGIAIGIGCGLGSIVI

VLGAMILANKWRKGIQKRIRRAYFKKNQGLLLEQLISNESATNKTKIFSLEELEEATNNF

DGTRVLGRGGHGTVYKGILSDQRVVAIKKSKIVEQTEIDQFINEVVILSQIIHRNVVKIF

GCCLESEVPLLVYEFISNGTLHDHLHTDLSVRCSLSWDDRIRIAVEAAGALSYLHSAAAI

PIFHRDVKSSNILLDGSFTTKVSDFGASRSVSLDETHVVTIVQGTFGYLDPEYYHTGQLT

EKSDVYSFGVILVELLIRKKPIFINEAGAKQSLSHYFVEGLQEGSLMEIIDPQVVEEANK

EEIDGIASLTMACLKVKGVDRPTMKEVEMRLQFLKTKRLRKFQLLPGNDGEIEHLLSPNT

SNSYAQNIYTNAGDLTSEGIPGSGCYSLEQELSSSISLPR

>OsWAK25

MRGAARLLLPLVVLLLHAARGSAGSTGGGGNGSCTQSCGRMRVPYPFGFSRGCTVQLGCD

DASGTAWLGGTRGLGLLVSNVTPRAIVLTLPPNCSRPLNESLDALFTDNYAPTAQNALVV

SSCDPQAAARLSNCSIPPEAYLEKSCNSIRCVLPSTKANVDGTNVTDPFLNRSEMRRLGS

DCRGLVSASIYSNTAGPALQLTALELDWWVQGRCGCSSHAICDGFTPPSTQKEAFRCECQ

EGFEGDGYTAGAGCRRVPKCNPSKYLSGSCGKLVQIGLLVAGVFFGAMVMGITCLVYHLL

RRRSAALRSQKSTKRLLSEASCTVPFYTYREIDRATNGFAEDQRLGTGAYGTVYAGRLSN

NRLVAVKRIKQRDNAGLDRVMNEVKLVSSVSHRNLVRLLGCCIEHGQQILVYEFMPNGTL

AQHLQRERGPAVPWTVRLRIAVETAKAIAYLHSEVHPPIYHRDIKSSNILLDHEYNSKVA

DFGLSRMGMTSVDSSHISTAPQGTPGYVDPQYHQNFHLSDKSDVYSFGVVLVEIITAMKA

VDFSRVGSEVNLAQLAVDRIGKGSLDDIVDPYLDPHRDAWTLTSIHKVAELAFRCLAFHS

EMRPSMAEVADELEQIQVSGWAPSTDDATFMSTTSSLCSSAPSRCTDKSWGTAKSKRQAA

ANAVVKQETTKCAVADSPVSVQERWFSDRSSPSSNSLLRNSSLNMAAAAALSAVAPGQRP

ADCPSECGGVDIPYPFGVDNCSWPGPDD

>OsWAK91

MMTIIQPPAMAMAMAMALLLLLLLQLWSVEAQVAAPPPASCPDRCGDVSVPYPFGIRDGC

HLPGFRLTCDATHTPPRLMLGNGTLQVVDISLANSTVRALDLAGAVNFTYDVSKLAPSGS

GTWSSLGTVAGAGPYVVSEQRNRLVVTGCNVQATLAGENTNIIGGCSSFCPVSEMFTSVA

ATVPVVPGAGADNATDGGFICSGTSCCETPIAIGRPSYLVQFLSLDQNQELTGKLPVAVR

IAERGWFEGVAGELLNTSSDSAAALRTPVPVVLEWVVSPTLEAVLQGVTGQFADDRNWSC

PADAARSACRSSNSFCSNVTGNYRRGYVCRCRRGYGGNPYVAGGCQDIDECKLAGRCYGE

CTNTPGDYQCRCPRGARGDPRIPNGCVKTNLGLSVGIGVGSGAGLLVMGLGAAFLKRKVK

KQRARMLRQKFFKQNRGHLLQQLVSQKADIAERMIIPLSELEKATNNFDKSRELGGGGHG

TVYKGILSDLHVVAIKKSKEAVQREIDEFINEVAILSQINHRNVVKLFGCCLETEVPLLV

YEFISNGTLYHHLHVEGPMSLPWEDRLRIATETARALAYLHSAVSFPIIHRDIKSHNILL

DGSLTTKVSNFGASRCIPAEQTGITTVVQGTLGYLDPMYYYTGRLTEKSDVFSFGVVLIE

LLTRKKPYSYRSPDDESLVTHFTALLTQGNLGDILDPQVKEEGGEEVKEIAVLAVACAKL

KVEERPTMR

>OsWAK92

MQRQAGGFISMAWSMPPLALFAAVLALQQAIAAAAAAGDCPTTCGDVAVPFPFGIGAGCY

HLPGFNLTCDRSSDPPRLLLGDAAAFQVLNVSIVNATVRAARVGGINITYGGGNTSSADE

GRGAWRGLGDGGPFALSEDRNELVVVWGCDVVALLTDGGGSGNSSNVTISGCASFCPGTD

AGGQAIAAPAGSTMSLTEDRRCTGVGCCQMPISVGRDSYQVRLRRLNPSPPQPPPPQGAG

DPTVVLIAEQGWVAEASRSTRGYPLPVTFDETAVPVLLGWMIASTRVGADGEVPVNSTCP

ADAARSACKSSHSSCRNVSSSARAGYVCDCDAGFHGNPYLATGCQDINECERAEEHGCFG

ECINTAGSFLCRCPAGMQGNYTQRNGCFRPPLPARSSTGLSIGVGVSSAASLILIVIMAI

FIIRKQKRRRAKKIRQKYFKQNRGQLLQQLVAQRADIAERMIIPLGELKKATNNFDRARE

LGGGGHGTVYKGILSDLHVVAIKKSKIAVQREIDEFINEVAILSQINHRNVVKLFGCCLE

TEVPLLVYEFVSNGTLYSHLHVSGPRSLPWSDRLRIATETAKAIAYLHSSVSIPIIHRDI

KSTNILLDDTLTSKVSDFGASRCIPVDQTGVTTKVQGTLGYMDPAYYYTQRLTEKSDVYS

FGVILVELLTRKKPFSHLTPEGEGLVAHFVTSFTEGNLVGVLDLQIMEEADMKVVEVVAT

LAVTCVNLRGEDRPTMRQVEMALEGIQASRENVSGNLSAEKLGESNNVARDFMPSQEGRS

MTEGTRQYSLEEEFLLSSRYPR

>OsWAK112d

MLMLLLNIRRQVQTLYLRESSISVARVQMGHLNIMLAFLSFVLLGLAEVEGTVALSQILS

NSHLITPYREVTARKFERRSLLQDHSSDDRRSSNASLPSAATLANCPKRCGNLSFDYPFG

IGDGCFRHPDFSLTCNATTQPPKLLLHINESVEVIDNIEVVGKDIAEFFYFNFFMVAFNH

LIPIKAGVDVYNLTWKAPGISFTISEMMIITVVSCDLDVFLIGQDNTPKLLCMVACPNKE

IADMVYMQDCEGPGCCTVLSETPVQAVQLQFVRHETSNAGKISNLSMLWDRINITIGAPL

VWSIVDQTRCSRNMEDNFACVSNHSGCITSVFRDIGYACQCNSGYKGNPFILDGCKHDSG

YNPRPEKHNCARQCGTITVPFPFGLEEGCSARKRFQLNCSDKTNSVLKFNDYFQVTYINV

SEGLLGIKHNSSLEEQLFNIMMEMMTSDNEPDLFVDPLESVSVQWAVANLTCQEAQHNTS

GYACVSTSSSCLNVLSSMDGYVGYRCSCLPGYRGNPYILDGCEDIDECRETPGICKGVCK

NTVGNYSCTKCPDHTEYDILRMQCTPIRKKSFYLGIIIGLSSGFGMLLLGLSGIVLIRRW

KRHAQKRLQTKYFRKNQGLLLEQLISSDENASEKTKIFSLEELKKATNNFDTTRILGRGG

HGTVYKGILSNQHVVAIKKAKVIRECEINDFINEVSILSQINHRNIVKLFGCCLETEVPL

LVYDFIPNGSLFGLLHPDSSSTIYLSWGDCLRIAAEAAGALYYLHSAASISIFHRDVKSS

NILLDANYTAKVSDFGASRSVPIDQTHIITNVQGTFGYLDPEYYQTRQLNEKSDVYSFGV

VLLELLLRKQPIFTINSGMKQNLCSYFLSEIKTRPITDMVDAQVLEEANEEDIKEVASLA

EMCLKLKGEERPTMKKVEMTLQLLRTKTMNSSQVDPTIDQEIQTVLTEGASDPEIQPLVT

NLDVDRANAASQRFQISCYSLEQEFLSSASLPRDISMDGLIKTKVLQNTTQYSVD

>SlWAK1

MQHYQVALFSFQLPCFMLILTLATAQIIPSNTTSPPTNSTSPPTNATAPAPSPTNTITKA

ANITKPGCPKQCGNVTVPYPFGIGSGCALDPMFEIDCNVTTPFIGNIQIYDISDAEMRIS

NFINTKCYSQTGVLIQDIPSWITLGTKSPYTFSTLNRFIVVGCDDGAIVSGNNFANGCPS

LCTSTNDIVKGKCMGFGCCQITIPKGLKFFNTTMVTTRNHSLIWSFNPCGHSFLGEASRF

EFQGIEDLSDVNFANKIRNNVPIVLDWAIGNLSCVEARKSNDYACLNNSQCVDSDTSLGG

YRCSCNSGYIGNPYIGSGCQDIDECADPNTNSCEKICTNIPGSYNCSCPEGYTGDGRKNG

RGCIAPNSNSEFPWIKFSVGMGVGFMSLVIGTTWLYFFIKKRKLIKLREKFFQQNGGLLM

KQRMSSNEGGVEATKIFTAAELKKATNNYASDRILGRGGNGIVYKGILSDNRIVAIKKSK

FMDEEQVEQFINEVLILTQVNHRNVVRLFGCCLEAEVPLLVYEYISHGTLYEHIHNRNGA

PWLSWENRLRVASETASALAYLHSSAQMPIIHRDVKSANLLLDDVYIAKVADFGASRLIP

IDQTHLATMVQGTLGYLDPEYFRTSQLTEKSDVYSFGVVLAELLTGMKPISKDRNEEEKN

LAEYFVLSMRRNQLFQILDRRVVREGSLEQLQKVAELVKSCLSLHGEDRPTMKEVASELE

NLRKFTKNNPWANGNGHEENEDELSDLYTIPIESNTDIDNFSGQYSSNSYTNSSNFSGQY

SSGSTSNTNSPLMKNRRAI

>TaWAK6

MSRTFQLLLVLALVGIVRVSGSRAHKNVTHGPHSCSGVDVPYPFGIVEDGGGGDYRAGFH

VMCDAGEPVLHTTGGDGKPVKIGNFSIQAAEARVWLPVVWQCYDSSGKPSRSDYRNLEFN

KGGVYRISNAKNNLFVLGCKTTGYLASQPDQGSGESTSYAQFTGCLCYCNNSQSAVNGAC

SGVGCCHVDIPPDLTDNWVAFMSYDHTDKVNFSPCDYAFVAEKKHYTFNTTDLKRALRQN

TWGWEMPVVLDWAIRDSPTCKEARKKEGYACISSNSLCLNSTNGPGYICNCRRGYEGNLY

IVDGCTDINECEHLDHYSCKGVCTNRQGSYECTCPKHTHSADPYKEVCSPNFPTNAKIIV

GAIGGLLVMVIMVFFWLLIEEKRKMKEHFEKVGGPTLEKLNNIKLFKKEDIRKIQKSSNI

IGSGGFGKVYKGCIGDNNELVAVKEPINVNSANKGQFANEIIIQSPVIHRNIVKLVGCCL

QVEVPILVYEFVPNGSLHDILHNGSRMHLDMCKRLKIAAESAEGLAYMHSKTTTTILHGD

VKPANILLNDEFTPKISDFGISRLIVTDMQHTGNVIGDMSYMDPVLLQTGLLTKKSDVYS

FGVVLLELITRKKASHSDKNSLLRNFLDAYTKDKSVIELVDKELAEVDREILDNLGEMIM

QCLNLDVNQRPEMTDVAERLRDMVKRFNAQ

>ZmWAK

MSSLLLRVLLIQLAAVECLSAAIPGCLTQCGGVEIPYPFGVGTNCSRKGFRIKCINGSAG

EEIPVLLPTTRYQNIRVLNLSVSPLPEARVLLPVAWQCFNAAGGVTGIYSGDVDFNPEGV

YRISNTQNELFVLGCDTYAFTKGVRVHNVNARFPYRYFTGCITVSVDEKDPRDGACAGLG

CCRVDIPPGITDTSMTFSSTWTRANQTFCPCDYAFIVEKGNYTFKASDLVSHTPDNRLPL

DWWTMPLRLDWAIRDNNGDSMTSISCAQAPNEPDYGCRSKHSECTNSTNGPGYFCKCAHG

YDGNPYVQSDGECTNINECQDPKSHNCSSGSKCIDTDGGYYCQCNFFRRGQQCDPLIPMA

AVALLTTFAAVVLGCVAIVLLQTLNNRKRFNRNGGKLLNAQGITTYTKRELKKITNGYSK

RLGGGHFGNVYEGTIVDGRKVAVKCPLRTRVSSHRCHWKNLIRPRRVPLPQQRVEEDGSF

MNEIRFQFEVSRHKNLVQLLGCCLETDIPILVFEFVANGSLEDILHSAKKPCTLSLPERL

DIAIGSAEAIAYMHSLDNQKRVHGDIKPSNILLDDDLNPKVSDFGSSKLLAIHSYYVRAV

AADIGYMDPLYMKTEHFTLECDVYSFGVVLLELITRRRASWYEQDQQGNKILPIEFVKCF

KDHGSGCAMYDSRLDFSGEDTQSRCNKRCLDMIGMLAVRCLKEDKRERPTMAEVVEELKR

VKVLLLGTHI

>ZmWAK-RLK1

MVCGYQREETPAGGMTFACFCDGGQTTGRCGAGSKRNEGPIVGAVVAVAFLCLVILTCFL

ACRHGSLPFKSKNKPGTRIESFLQKNESSIHPKRYTYADVKRMTKSFAVKLGQGGFGAVY

KGSLHGRQVAVKMLKDTQGDGEEFMNEVASISRTSHVNVVTLLGFCLQGSKRALIYEYMP

NGSLERYAFTGDMNSENLLSWERLFDIAIGTARGLEYLHRGCNTRIVHFDIKPHNILLDQ

DFCPKISDFGLAKLCLNKESAISIVGARGTIGYIAPEVYSKQFGTISSKSDVYSYGMMVL

EMVGARDRNTSADSDHSSQYFPQWLYEHLDDYCVGASEINGETTELVRKMIVVGLWCIQV

IPTDRPTMTRVVEMLEGSTSNLELPPRVLLS
